# The spinach YY genome reveals sex chromosome evolution, domestication, and introgression history of the species

**DOI:** 10.1186/s13059-022-02633-x

**Published:** 2022-03-07

**Authors:** Xiaokai Ma, Li’ang Yu, Mahpara Fatima, William H. Wadlington, Amanda M. Hulse-Kemp, Xingtan Zhang, Shengcheng Zhang, Xindan Xu, Jingjing Wang, Huaxing Huang, Jing Lin, Ban Deng, Zhenyang Liao, Zhenhui Yang, Yanhong Ma, Haibao Tang, Allen Van Deynze, Ray Ming

**Affiliations:** 1 Center for Genomics and Biotechnology, Fujian Provincial Key Laboratory of Haixia Applied Plant Systems Biology, Key Laboratory of Genetics, Breeding and Multiple Utilization of Crops, Ministry of Agriculture, Fujian Agriculture and Forestry University, Fuzhou, 350002 China; 2 Department of Plant Biology, University of Illinois at Urbana-Champaign, Urbana, IL 61801 USA; 3 Department of Plant Sciences, University of California, Davis, CA 95616 USA; 4 USDA-ARS, Genomics and Bioinformatics Research Unit, North Carolina 27695 Raleigh, USA

**Keywords:** Spinach, Downy mildew resistance, Frost resistance, Domestication, Sex determination

## Abstract

**Background:**

Spinach (*Spinacia oleracea* L.) is a dioecious species with an XY sex chromosome system, but its Y chromosome has not been fully characterized. Our knowledge about the history of its domestication and improvement remains limited.

**Results:**

A high-quality YY genome of spinach is assembled into 952 Mb in six pseudo-chromosomes. By a combination of genetic mapping, Genome-Wide Association Studies, and genomic analysis, we characterize a 17.42-Mb sex determination region (SDR) on chromosome 1. The sex chromosomes of spinach evolved when an insertion containing sex determination genes occurred, followed by a large genomic inversion about 1.98 Mya. A subsequent burst of SDR-specific repeats (0.1–0.15 Mya) explains the large size of this SDR. We identify a Y-specific gene, *NRT1/PTR 6.4* which resides in this insertion, as a strong candidate for the sex determination or differentiation factor. Resequencing of 112 spinach genomes reveals a severe domestication bottleneck approximately 10.87 Kya, which dates the domestication of spinach 7000 years earlier than the archeological record. We demonstrate that a strong selection signal associated with internode elongation and leaf area expansion is associated with domestication of edibility traits in spinach. We find that several strong genomic introgressions from the wild species *Spinacia turkestanica* and *Spinacia tetrandra* harbor desirable alleles of genes related to downy mildew resistance, frost resistance, leaf morphology, and flowering-time shift, which likely contribute to spinach improvement.

**Conclusions:**

Analysis of the YY genome uncovers evolutionary forces shaping nascent sex chromosome evolution in spinach. Our findings provide novel insights about the domestication and improvement of spinach.

**Supplementary Information:**

The online version contains supplementary material available at 10.1186/s13059-022-02633-x.

## Background

Dioecy is rare in plants, occurring in ~6% of angiosperms species and ~10% of land plant species [1, 2]. Most sex chromosome sequences in angiosperm are at an early stage of evolution. Sequencing male and female genomes and defining sex determination regions (SDRs) in dioecious angiosperm species will contribute to uncover the evolutionary process leading to dioecy [2]. Several models have been proposed to explain the evolution of sex chromosomes from an autosome via male- and/or female-sterile mutations followed by recombination suppression of sex-linked region and the divergence of XY or ZW chromosomes or via single regulatory factor [2–5]. Sex-linked regions could potentially remain as small as the initial sex-determining mutations, or could subsequently evolve into large non-recombining regions at later stages of sex chromosome evolution [6, 7]. Despite advances in genomic technologies, it has been difficult so far to completely assemble sex chromosomes, particularly those in the later stages of sex chromosome evolution that have accumulated repetitive sequences, and undergone structural variations or gene degradation. However, plant sex chromosome systems (either ancient or young) at early stage bearing minimal variations between XY or ZW chromosomes can provide a unique opportunity and good model to study the initial formation of sex chromosome evolution [6–9].

Sex determination systems are polyphyletic in plants, and recent advances in genomic and molecular biology techniques have elucidated the sex chromosome evolution in several species across diverse lineages by sequencing their SDR and its X(Z) counterpart with different strategies. An 8.1-Mb hermaphrodite-specific region of the Y chromosome (HSY) in papaya (*Carica papaya*) was characterized using a BAC-BAC sequencing strategy [10]. In *Ficus*, a 2-Mb SDR containing 93.64% repetitive sequences was identified by splitting male- and female-specific reads for the assembly of two haplotypes [11]. In *Salix purpurea*, the SDR occupies a large portion of the W chromosome with ceased recombination extending ~ 6.8 Mb [12, 13]. It is noteworthy that extent of suppressed recombination (size of the SDR) does not correlate to the age of sex chromosomes [6, 7]. The smaller SDRs either anciently evolved in *Vitis* species (~150 kb size, 16.5 Mya age), *Populus trichocarpa* and *P. balsamifera* (~100 kb, 7.2 Mya), or recently evolved in *Fragaria* taxa (13 kb female-specific SDR, 1.1 Mya), and *Asparagus* (1Mb, 3 Mya), make good candidates to study the initial formation of sex chromosomes [5, 14–20]. Although assembling sex chromosomes can be complicated, sex determination genes have recently been identified in several plant species. The SDRs of kiwifruit, asparagus, and *Populus deltoides* each contain two male-specific candidate sex determination genes [18, 21, 22], while single genes causing male fertility and female abortion were identified in persimmon (*Diospyros spp.*), *Salix purpurea*, and *Populus tremula* [3, 5, 12].

Characterizing a SDR and its X or Z counterpart provides fundamental genomic information for understanding sex chromosome evolution [4]. In XY/ZW genotypes, highly repetitive sequences and mixtures of sex-specific reads from non-recombining SDRs have hindered the haplotype phasing of these SDRs and assembly of complete Y or W chromosomes. However, these problems can be overcome in species with less divergent sex chromosomes in early stages of evolution and a viable YY genotype. For instance, the viability of the YY genotype in *Asparagus* enabled the identification of two sex determination genes in the ~1 Mb SDR through the separate sequencing of YY and XX genomes via a combination of short- and long-read sequencing technologies with optical mapping [17].

*Spinacia oleracea* L. (2n=2×=12) is a dioecious species with some androdioecious populations that segregate viable YY genotypes [23], making it an ideal system to study sex determination and sex chromosome evolution. The abortion of carpels or stamens in unisexual flowers occurs at the initiation of primordia with no trace of carpels in male flowers or of stamens in female flowers. Previously, sex-linked markers were developed for mapping the spinach sex-determining locus to the largest linkage group [24–26]. Although high-density genetic maps defined an 18.4-Mb X counterpart of the SDR in spinach, the complete SDR was not revealed [27]. A published spinach draft genome anchored 47% of the assembly into six pseudomolecules, although the sex type of this genome was not described [28]. The viability of the supermale YY genotype in spinach [23] offers a rare opportunity to sequence a supermale genotype to enhance the quality of the assemblies of both the overall genome and the Y chromosome and acquire new knowledge of sex chromosome evolution.

Cultivated spinach was first recorded in ancient Persia approximately 2000 to 3000 years ago [29]. Its wild relatives *S. turkestanica* and *S. tetrandra* have been proposed as spinach ancestors and represent potential germplasm sources for spinach improvement [30]. Although several edibility traits such as enlarged leaves and elongated internodes have become domesticated, the genetic introgression of resistance to diseases such as downy mildew and abiotic stresses such as chilling and frost resistance from wild species remains a challenge for spinach breeding [31].

Viable supermale individuals generated from selfed XY androdioecious spinach [23] allowed us to generate a high-quality genome assembly of the YY genotype using PacBio sequencing and reference-guided Hi-C-scaffolding based on a female XX genome. These chromosome-scale assemblies of both genotypes enabled our analyses to define the SDR, assemble X and Y haplotypes in the SDR of the sex chromosomes, and compare their structural variations and evolutionary landscapes. Further, by combining analyses of genomic information with transcriptome profiles of female and male flowers at different developmental stages, we could propose candidate genes for and a model of sex determination or differentiation in spinach. Finally, we resequenced 112 genomes of spinach and its wild relatives to dissect its origin, gene flow, trait domestication, and improvement through genomic introgression.

## Results

### Genome analysis of supermale “Cornell-NO. 9”

**Fig. 1:**
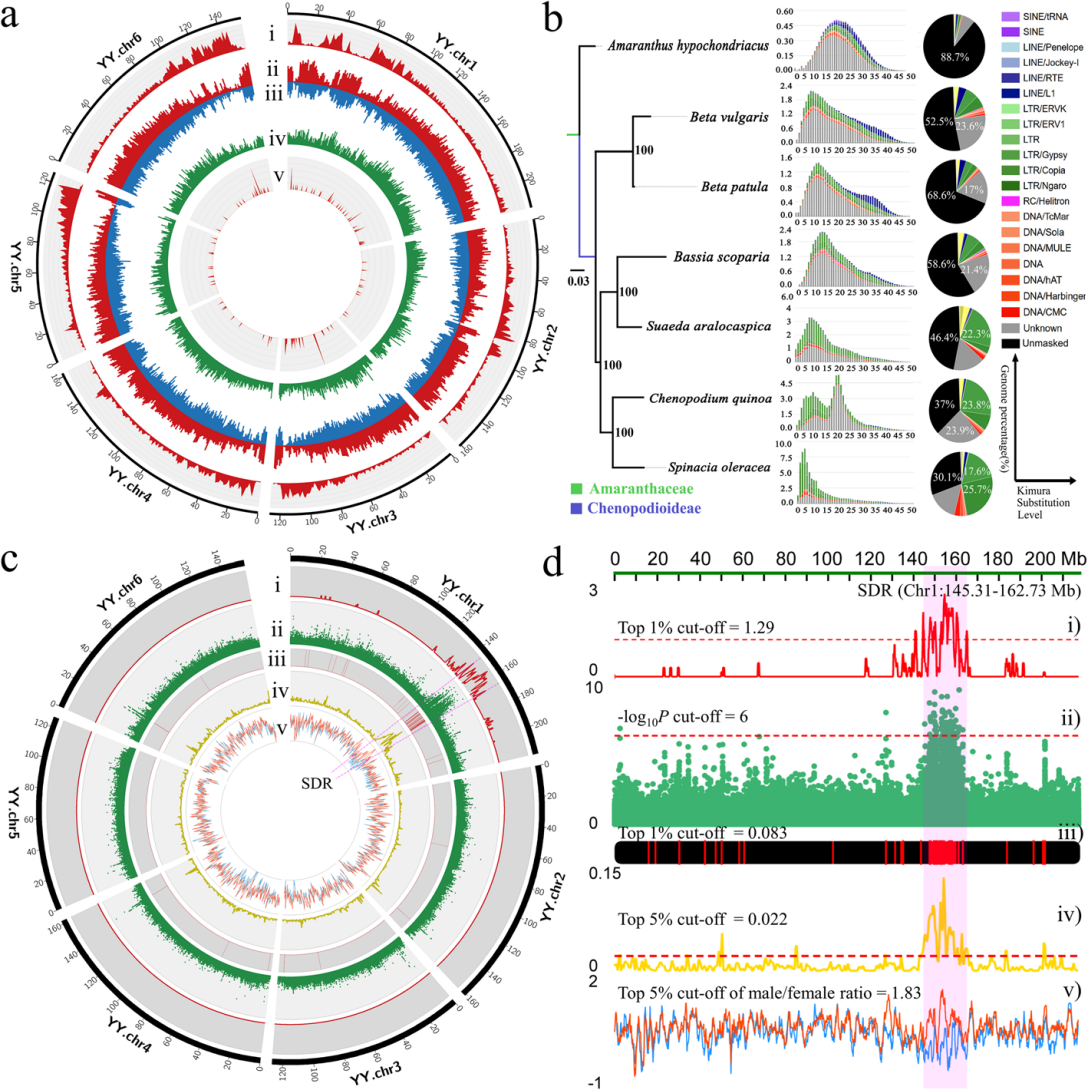
Genomic features and genome evolution of *Spinacia oleracea* “Cornell-NO. 9” (YY genome), and Sex determination region (SDR) definition. **a** Genomic features of “Cornell-NO. 9” (YY) of *S. oleracea*. (i) Gene density; (ii) LTR transposable elements (*Copia*); (iii) LTR transposable elements (*Gypsy*); (iv) DNA transposable elements; (v) Predicted miRNA density. **b** LTR burst patterns and fractions of different repeat elements among the genomes of *S. oleracea “*Cornell-NO. 9” (YY) and congener species within the Amaranthaceae family. **c** Identification of the sex chromosome and SDR (sex determination region) among six chromosomes of the YY genome. (i) Density distribution of co-segregation bins (contigs) (100-kb window); (ii) GWAS Manhattan plot of two sex phenotypes (26 females and 44 males); (iii) Density distribution of male-specific SNPs (20-kb window); (iv) genetic differentiation (*Fst*) between females and males (1000-kb window); (v) Tajima’s D of males (red line) and females (blue line) (200-kb window). **d** Close-up view of the identified SDR (sex determination region) along chromosome Y. Two clear boundaries of the sex determination region (SDR) (145.31–162.73 Mb, size = 17.42 Mb) were defined using the overlapping region of following evidence (from top to bottom): (i) Elevated density of sex co-segregation bins (contigs) with top 1% cutoff = 1.29; (ii) GWAS plot of two sex phenotypes with cutoff of −log_10_(P) = 6; (iii) Density distribution of male-specific SNPs with top 1% cutoff = 0.083; (iv) *Fst* between females and males with top 5% cutoff = 0.022; (v) Tajima’s D of males (red line) and females (blue line) with top 5% cutoff of male/female Tajima’s D ratios =1.83

Androdioecious XY individuals of spinach variety “Cornell-NO. 9” (PI 217425) were used to develop populations of segregating YY supermale individuals that were identified using DNA markers [23]. De novo assembly of the YY genome was performed by incorporating 66 Gb of PacBio long reads generated from a total of 135 single-molecule real-time (SMRT) cells sequenced on the PacBio RSII system (Additional file 1: Supplementary Notes; Additional file 3: Tables S1). The resulting YY contig assembly yielded 948 Mb of sequence at 94.76% completeness (Additional file 2: Fig. S1; Additional file 3: Table S2). In order to anchor and orient the contigs of the YY genome, a Hi-C map-based chromosome-scale genome assembly of the female XX genome of spinach cultivar “Viroflay” [32] was adopted as the reference to build a chromosome-scale YY genome assembly (Additional file 1: Supplementary Notes; Additional file 3: Table S2-S4). The YY contig assembly was grouped according to the XX pseudomolecules using a reference-guided strategy with Ragoo [33], then anchored by Hi-C physical mapping with 100× Hi-C data (Additional file 1: Supplementary Notes; Additional file 3: Table S3, S4). Finally, 950 Mb (99.79% anchored) of the YY genome was anchored into six pseudo-chromosomes and formed 952 Mb final chromosome assembly (Fig. 1a; Additional file 3: Table S4). This chromosome assembly was then validated by a well-organized pattern of contacts along the diagonals of each chromosome based on chromatin interaction data (Additional file 2: Fig. S2). Genome annotation resulted in 26,910 gene models with 90.25% BUSCO completeness (Additional file 1: Supplementary Notes; Additional file 3: Table S4).

The spinach YY genome contains a high proportion of repetitive sequences (74.00%), including 57.4% retrotransposons (20.3% *Ty1*/*Copia* and 14.98% *Ty3*/*Gypsy*) and 13.03% DNA transposons (Additional file 1: Supplementary Notes; Additional file 3: Table S5). Comparison of the repetitive element fractions in the spinach YY genome using Kimura substitution levels (KSL) among six genera within Amaranthaceae revealed their different proliferation patterns (Fig. 1b). Recent retrotransposon burst amplifications (KSL < 15) were detected in five genera, except for *Amaranthus*, and the most recent species-specific retrotransposon burst (KSL < 5) that found exclusively in *Spinacia* was caused by *Copia* and *Gypsy* LTR-RTs (Fig. 1b).

Collinearity analysis of the YY genome of “Cornell-NO. 9” and the XX genome of “Viroflay” [32] (Additional file 2: Fig. S3) revealed 18,326 (> 67.0%) orthologous gene pairs within 435 conserved syntenic blocks. Between these two assemblies, 7792 genes (14.3%) appeared to have been rearranged, with 146.76 Mb (4034 genes) XX and 133.79 Mb (3758 genes) of YY sequences affected.

### Identification of sex chromosomes and SDR

Two high-density F_1_ genetic maps were constructed with 79 resequenced F_1_ individuals from a cross between the spinach cultivars “Viroflay” and “Cornell-NO. 9” parental lines (Additional file 1: Supplementary Notes; Additional file 3: Table S6). Overall, 19,815 bins (369,524 SNPs after filtering, Additional file 1: Supplementary Notes; Additional file 2: Fig. S4a) and 5362 bins (41,876 SNPs) were anchored to six linkage groups (LGs) in the male and female maps with 428.99 cM and 412.78 cM genetic distance, respectively (Additional file 2: Fig. S5a,b). The female genetic map exhibited fewer SNPs, which corresponds to the higher homozygosity of female parents generated by self-pollination of monoecious lines, whereas the male parents were generated by cross-pollination that result in higher heterozygosity. Genotype screening retrieved 322 sex-co-segregation bins (contigs) (2690 SNPs) distributed with an average of 0.148 sex-co-segregation contigs in100-kb sliding window across the Y chromosome. The top 1% (cutoff value = 1.29) density of sex co-segregation contigs was aggregately distributed from Chr1: 141.1–165.7 Mb (Fig. 1c,d).

Similarly, a high −log_10_(*P*) score (cutoff of −log_10_ (*P*) = 6) of GWAS mapping derived from two sex phenotypes (26 resequenced female genomes and 44 male genomes) peaked at Chr1:145.0–162.7 Mb between the two sex phenotypes (Additional file 1: Supplementary Notes; Additional file 2: Fig. S4b; Additional file 3: Table S13). This region also contained a high density of male-specific SNPs (Chr1:143.6–163.5 Mb) from top 1% sliding windows (window size = 20 kb) of whole genome*,* the top 5% peak of genetic differentiation (*Fst*) (Chr1: 145.3–167.0 Mb) between the two sexes in a 1000-kb sliding window, and divergent Tajima’s *D* value (top 5% cutoff of male/female ratios in a 200-kb window) between two sexes at Chr1:141.4–163.6 Mb (Fig. 1c,d). These multiple lines of evidence and taking overlapped regions screened by respective cutoffs led to our identification of spinach chromosome 1 as the sex chromosome and 145.3–162.7 Mb is the approximate location for sex determination region (SDR). Finally, two boundaries of SDR were defined at Chr1:145.31–162.73 Mb (size = 17.42 Mb) based on genomic position of two terminal contigs of this region on Y chromosome (Fig. 1c,d).

### Comparative analysis of the spinach Y chromosome SDR and its X counterpart

**Fig. 2:**
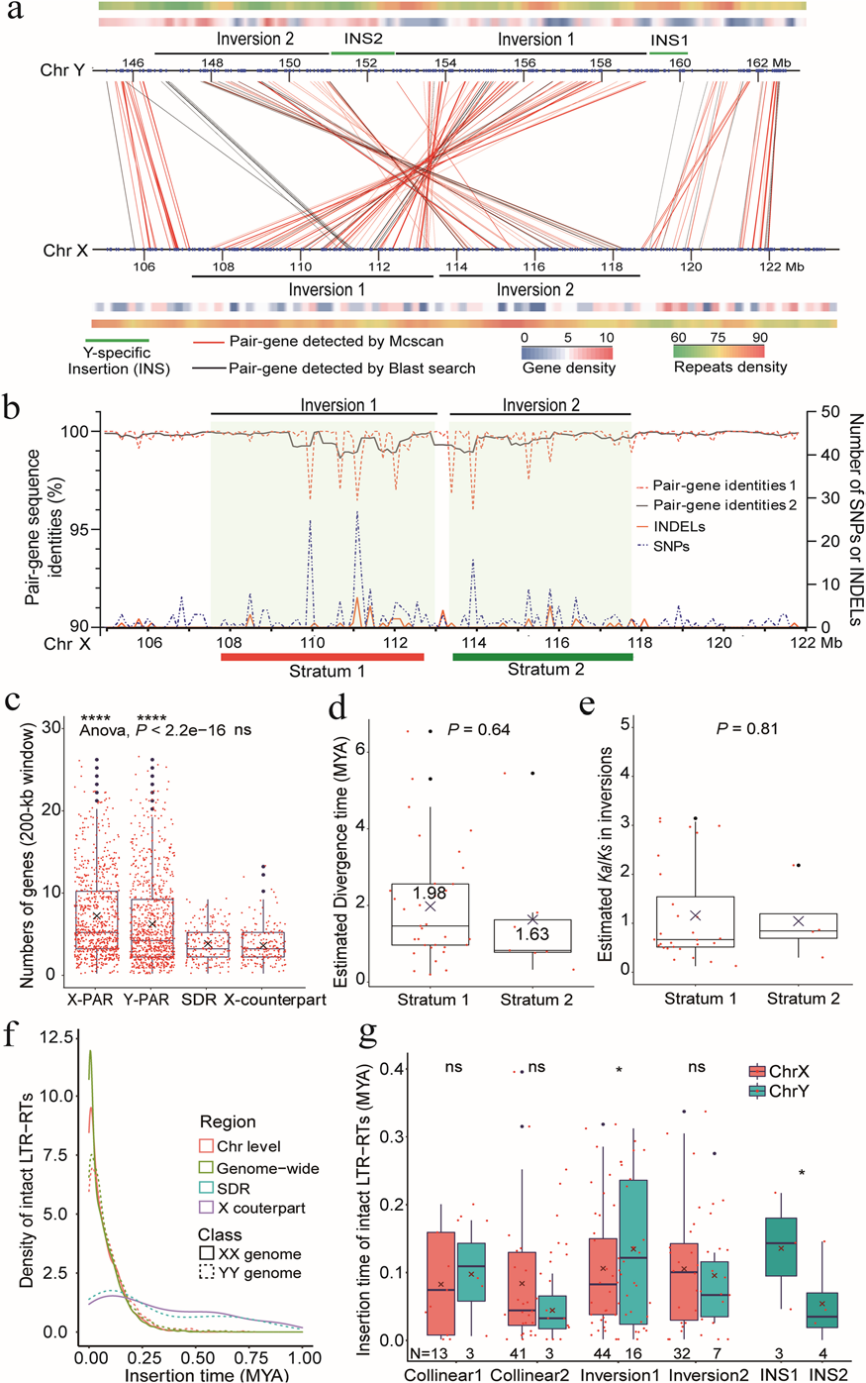
Genomic features of the Y-chromosome SDR and its X counterpart in spinach. **a** Genomic landscape of the SDR and its X counterpart. The red and black lines connecting gene pairs identified using MCscan and blast search, respectively. The region is featured by two Y-specific insertions (INS1 and INS2) and two inversions (Inversion 1 and Inversion 2). **b** Comparison of 126 conserved gene pairs from the SDR and its X counterpart. Sequence identities (identities 1 without sliding window, identities 2 with sliding window in 500kb window and 200kb step), numbers of SNPs, and INDELs were plotted between each gene pair. Inversion 1 and 2 were classified as two strata separated by INS2. **c** Comparison of gene densities among different regions. Gene densities in 200-kb window were plotted in four genomic regions, i.e., ChrX-PAR, ChrY-PAR, the SDR, and its X counterpart. Mean separation was performed using *t*-test and ANOVA (alpha = 0.05). **d** Gene pair divergence among different genomic regions. The SDR was classified into two strata based on two inversions. An estimated divergence time (T = *Ks*/*2r*, *r *= 2.8e−9) was plotted for each stratum with pairwise *t*-tests. **e** Similarly, *Ka*/*Ks* ratios for each gene pair from respective strata were plotted. **f** Comparison of LTR-RTs insertion times among different genomic regions. Density distributions of intact LTR-RTs insertion times in the SDR and its X counterpart, entire X and Y chromosomes, and whole YY and XX genomes were plotted. **g** Insertion times of LTR-RTs across the SDR and its X counterpart, including collinear regions, two inversions and two insertions

Based on the synteny between the YY and XX genomes, a 16.23-Mb (XX Chr1: 105.75–121.98 Mb) X counterpart of the SDR was mapped to chromosome 1 of the spinach XX genome. The SDR contains 307 annotated genes along with 278 annotated genes from the X counterpart (Fig. 2a; Additional file 2: Fig. S5c; Additional file 3: Tables S7,8). A total of 126 conserved gene pairs (56.31%, cs-score = 0.65) from synteny blocks within the SDR and X counterpart were detected using MCscan (Additional file 3: Table S7,8,9). Inversions and insertions were detected via microsynteny analysis using MCscan. We defined regions with inverted syntenic orders as inversions and those with no syntenic genes as insertions (Fig. 2a,b). The genomic landscape of the sex-linked region includes two inversions (Inversion 1 and Inversion 2) and two Y-specific insertions (INS1 and INS 2). The region was revealed through combined analyses of microsynteny between pairs of alleles, gene density, and repetitive sequence proliferation (Fig. 2a,b; Additional file 2: Fig. S5c; Additional file 3: Table S8,9). The two inversions were numbered based on their estimated time of divergence from earliest to latest, the larger inversion 1 occurred earlier, about 1.98 million years ago (Mya), and was designated as stratum 1, whereas the smaller inversion 2 occurred at 1.63 Mya, and was designated as dispersed stratum 2. Although estimates of their divergence times (*P* = 0.64) and the *Ka/Ks* ratio (*P* = 0.81) among paired genes did not differ significantly, their dispersed distribution from two sides separated by INS 2 warrants designation of separate strata (Fig. 2b–e).

Gene density across the SDR was significantly lower than that of the PARs (pseudoautosomal regions) (*P* < 2.2e−16, Fig. 2c), and two Y-specific regions were enriched with repetitive sequences (Fig. 2a). We confirmed the presence of these structural variations by estimating the mapping depth of PacBio reads associated with these regions. For each 8-kb window, junctions of inversions and insertions were covered by an average of 96.99 reads, while junctions of inversions or insertions and collinear regions were covered by an average of 79.33 reads (Additional file 3: Table S10, Additional file 2: Fig. S6). Thus, our definition of inverted and inserted regions in the SDR should be less prone to artifacts from assembly.

Genome-wide proliferation of intact LTR-RTs from both YY and XX genotypes peaked around 0.025 Mya (Figs. 1b and 2f; Additional file 1: Supplementary Notes). A total of 36 and 130 intact LTR-RTs were identified in the SDR and its X counterpart, respectively. Interestingly, both the peaks of LTR-RTs proliferation from SDR and its X counterpart were differentiated compared to respective chromosomal-level and genome-wide pattern (Fig. 2f). Intact retrotransposons from inversion 1 within SDR were dated earlier compared with X counterpart (Fig. 2g). Moreover, these LTR-RTs within SDR and X counterpart were dated later than the stratum 1 formation (Fig. 2d,f,g).

Invasions of sequences from the chloroplast genome, or nuclear integrants of plastid DNA (NUPTs), were distributed widely in whole spinach genomes, but the largest proportion of NUPTs in terms of both number (27.7%) and length (65.7%) was identified in the Y chromosome (Additional file 1: Supplementary Notes; Additional file 2: Fig. S7). The highest densities of NUPTs were detected within a 112-kb region on Y and a 129-kb region on X chromosome, but not in the SDR. Sex chromosome-specific miRNAs were enriched at intergenic regions of the Y chromosome (15) and X chromosome (9) (Additional file 1: Supplementary Notes; Additional file 2: Fig. S8).

### Sex-biased expressed patterns highlight candidate genes for sex determination or differentiation

Because genes with sex-biased expression might be related to sex determination or differentiation, we characterized the expression profile of female (F) and male (M) flowers from five stages (S1 through S5) and grouped them as “early stage” (S1-S2) and “late stage” (S3-S5) with 1246 and 2499 shared DEGs (differentially expressed genes), respectively (Additional file 1: Supplementary Notes; Additional file 2: Fig. S9, S10). The annotations of DEGs located on sex chromosomes were enriched for GO and KEGG terms including “plant hormone signal transduction” and “reproductive processes,” suggesting potential roles in sexual divergence (Additional file 1: Supplementary Notes; Additional file 2: Fig. S11e,f). In total, 73 plant hormone-related DEGs were identified, 13 of which displayed male-specific expression, and one, a brassinosteroid-related gene, showed female-specific expression (Additional file 2: Fig. S12a,c)*.* A total of 221 DEGs encoding TFs (transcription factors) were identified, most of which belong to eight TF families (Additional file 2: Fig. S12b,d) including four NAC, one bHLH, one LBD, and four MADS-box genes with male-specific expression, and one *SUP*-like C2H2 with female-specific expression.

**Fig. 3:**
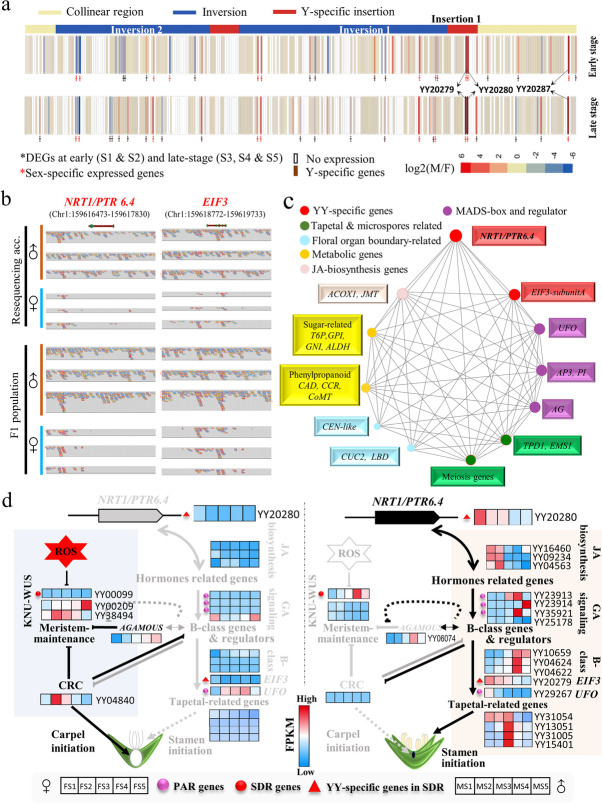
Proposed mechanism for spinach sex determination and differentiation. **a** Expression pattern of genes within the SDR at early and late flower development stages between male and female flowers. Two Y-specific genes (YY20280, YY20279) are located in Insertion 1 flanking the largest Inversion 1. **b** Alignments of male and female Illumina reads to the YY genome revealed the presence/absence variants (PAV) *NRT1*/*PTR 6.4* and *EIF3-subunit A* in male and female individuals, which confirmed them as Y-specific genes. **c** Visualization of Y-specific (*NRT1*/*PTR6.4* and *EIF3*) genes in their first-degree gene network at stage 1 (S1). Gene clusters putatively related to different functions are indicated with different colors. The sizes of the nodes reflect the number of edges connected to other nodes in the network. **d** Proposed pathway for single-factor sex determination in spinach. *NRT1/PTR6.4* can integrate two independent pathways to promote stamen initiation and suppress carpel development. The proteins encoded by the B-class (*APETELA3*, *PISTILLATA*) genes can function as intermediates in these pathways. These B-class genes might be activated by synergistic or independent action of hormones including gibberellins (GA) and jasmonates (JA). *NRT1* may transport GA and JA to induce stamen initiation, thus repressing *CRC* expression, or inhibit the *AG-KNU-WUS* interaction, resulting in meristem termination failure and carpel suppression in male flowers (right side). A reverse process might occur in the absence of *NRT1* gene and protein in female flowers (left side). In dioecious spinach, only male individuals with a Y-chromosome stably express *NRT1*. Black text refers to activation, while gray text refers to inhibition of the pathway

Among 307 genes in the SDR, 36 (10 at “early stage”, 13 at “late stage”, and 13 at “all stages”) showed differential expression (Additional file 2: Fig. S13a−c; Additional file 3: Table S11). As spinach sex determination occurs at the initiation of stamen or carpel primordia, the 23 (10 + 13) genes found at “early stages” and “all stages” are potential candidate genes for the control of sex differentiation or determination. Among 36 DEGs, mostly are in inversion 1 (eight sex-biased, four sex-specific expressed) and inversion 2 (10 sex-biased, four sex-specific expressed), while only three DEGs are in Y-specific insertion 1 (Fig. 3a; Additional file 3: Table S11). Apart from the SDR, 689 DEGs were located in PAR with five YY-specific genes (Additional file 2: Fig. S13d, e).

To further narrow down the candidate genes for sex determination or differentiation, YY-specific genes were identified using three methods (Additional file 2: Fig. S14; Additional file 3: Table S8). In Method (i), 82 genes from the SDR were identified using MCScan; (ii), 81 genes on the Y chromosome were identified using blast searches; and (iii) 149 genes in the YY genome were identified by retrieving Y-specific contigs using a k-mer-based method (Additional file 1: Supplementary Notes; Additional file 2: Fig. S14a). In total, nine YY-specific genes in the SDR and 15 in PARs were selected based on positive results in each of the three methods (Additional file 2: Fig. S14b). Among these, three genes in the SDR showed differential expression between sex types (Fig. 3a). YY20280 (*NRT1*/*PTR family 6.4*) was identified by all three methods and had the highest expression among Y-specific genes throughout male flower development and could thus be considered as a candidate gene for sex determination or differentiation. Two other potential candidates, YY20279 (*EIF3* subunit A-like) and YY20287 (rRNA-processing protein *FCF1*), showed significantly higher expression in male flowers (Additional file 3: Table S11). Further alignments of male and female Illumina reads to the YY genome confirmed the exclusive presence of *NRT1/PTR 6.4* and *EIF3* in Y-specific INS1 (insertion 1) flanking the oldest Inversion 1 (Figs. 2a and 3a,b).

### Core gene network correlated with *NRT1* and *EIF3* expression

To identify core genes that potentially cooperate with Y-specific candidates, a coexpression network was generated using WGCNA analysis. DEGs between male and female flowers at stage 1 (S1) were clustered into four modules (M1 through M4) (Additional file 2: Fig. S15a). Y-specific genes *NRT1* (YY20280) and *EIF3* (YY20279) were nested in a male-module (M2, *r* = 0.97, *P* = 0.002) and directly connected to 40 stamen development-related genes grouped by function as MADS-box and regulators *(AG, AP3, PI, UFO*), JA-biosynthesis (*ACOX1*, *JMT*), tapetum-related (*EMS*, *TPD1*, and genes involved in meiosis), sugar-related, phenylpropanoid pathway, and floral-organ boundaries (*CUC*, *LBD*, *CEN-like*) genes. All these genes exhibited significantly higher expression in male flowers at initiation (S1) (Fig. 3c; Additional file 2: Fig. S15e). In *Arabidopsis*, *GTR1*/*NPF2.10*, which encode transporters in the same family as *NRT1*, were coexpressed with JA-biosynthesis genes that regulate gibberellin-mediated stamen development [34]. In *Arabidopsis*, *ems1*/*tpd1* mutants result in male sporocytes that exhibit meiotic defects and thus fail to produce microspores and tapetum [35]. *EMS*/*TPD1* and many other meiosis-related genes that exhibit male-biased expression in spinach were directly connected to *NRT1* and *EIF3* in the coexpression network, further suggesting the involvement of Y-specific genes at initial stages of stamen development (Additional file 2: Fig. S15e). Moreover, genes related to meristem termination and gynoecium development (*CRC*, *HEC2*) [36] and ROS-related (reactive oxygen species) genes were nested in the female module at stage 1 (M3, *r* = 0.89, *P* = 0.02). Emerging evidence indicate that ROS homeostasis activates or represses WUSCHEL activity to balance stem cell identity and differentiation [37].

Based on direct links between Y-specific genes (*NRT1, EIF3)* in the coexpression network and genes involved in *Arabidopsis* flower organ specification [38, 39], we were able to define two separate pathways governing stamen and carpel identity (Fig. 3d; Additional file 3: Table S12), although downstream mechanisms of organ development might be similar in diverse plant species [38, 39]. The expression of several candidate genes was verified by qRT-PCR (Additional file 1: Supplementary Notes; Additional file 2: Fig. S16). When relative expression at stage 1 was compared, *NRT1* showed male-specific expression while *EIF3* showed non-significant but higher expression in male floral buds.

### Genetic diversity and domestication bottlenecks in spinach

**Fig. 4:**
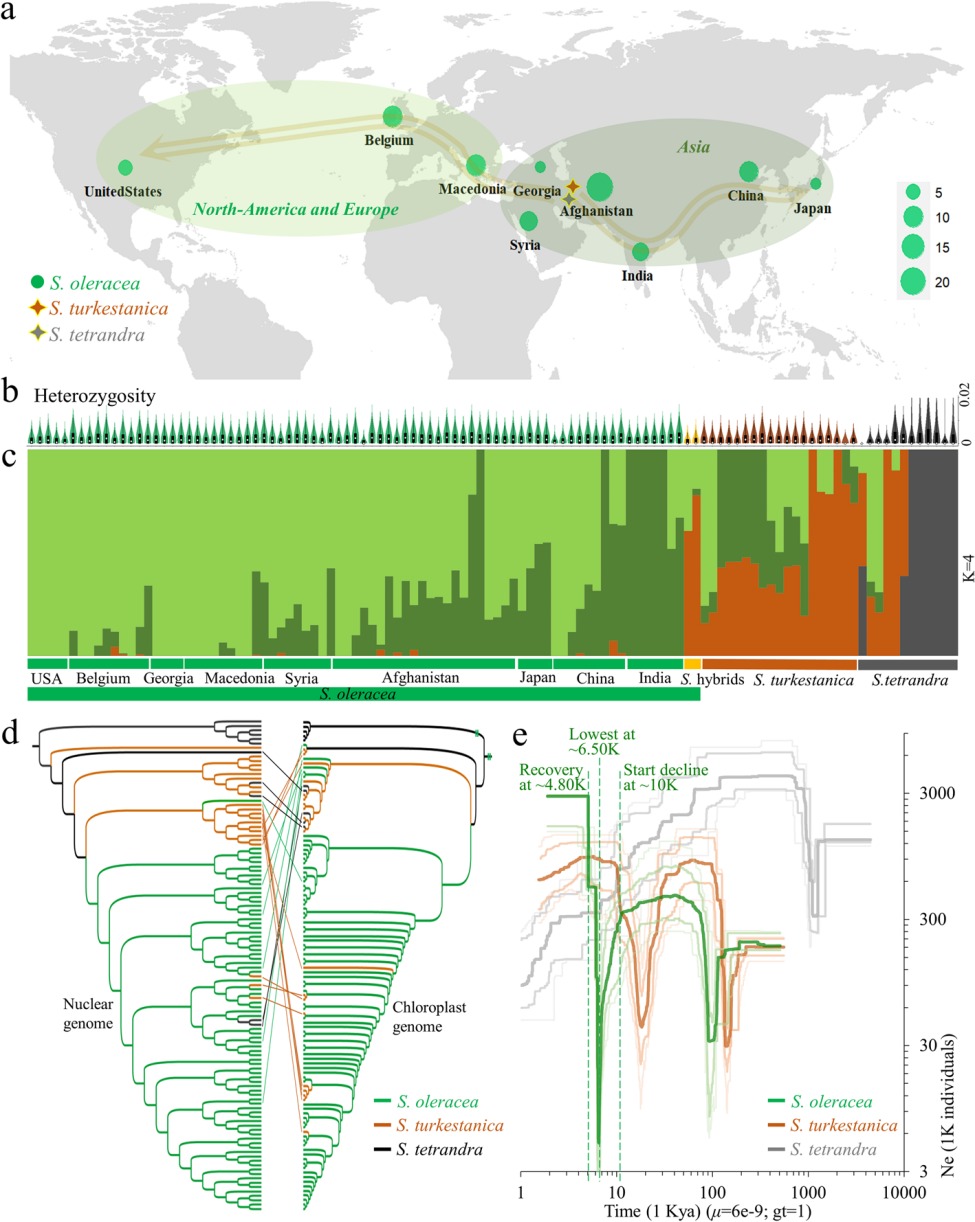
Population genomic analyses of 112 spinach accessions including 81 representative *S. oleracea* (including two hybrids), 11 *S. tetrandra*, and 20 *S. turkestanica* varieties. **a** The proposed origin and evolutionary route of *S. oleracea* varieties. After origination from the Middle East area, *S. oleracea* dispersed to nine geographical regions with two branches: Asia (Afghanistan, Syria, Georgia, India, China, and Japan), and Europe and North-America (Macedonia, Belgium, and the USA). **b** Heterozygosity rates. **c** Population structure analysis clustered 112 accessions into four subgroups (K = 4) and divided *S. oleracea* into nine subgroups. **d** Phylogenetic relationships among 112 accessions discovered using nuclear genome SNPs and whole chloroplast genomes, revealing a conflict between phylogenies based on the nuclear and chloroplastic genomes of *S. oleracea* and wild species. **e** Historical effective population sizes (*N*_*e*_) for the domesticated *S. oleracea*, compared to wild *S. turkestanica* and *S. tetrandra*, estimated using the nucleotide substitution rate *μ* = 6e−9 and generation time gt = 1. The *S. oleracea* population has undergone one recent *N*_*e*_ bottleneck ~10.87 Kya, reached its minimum value at ~6.5 Kya, and recovered well ~4.8 Kya, although the two wild populations did not experience this bottleneck. The *S. turkestanica* population has undergone two periods of ancient geologic upheaval during late Tarantian Age at 148–177 Kya and Holocene at 18.97–26.51 Kya. The *S. tetrandra* population experienced one *Ne* decline from the Chibanian to Calabrian ages at 1.035–1.321 Mya. The estimates are the medians (one thick line) from 200 bootstrap replicates with 2.5%, 12.5%, 87.5% and 97.5% confidence intervals (four thin lines)

From 112 resequenced genomes, including 81 *S. oleracea* (including two hybrids), 11 wild *S. tetrandra*, and 20 *S. turkestanica* (Fig. 4a–d; Additional file 3: Table S13), we identified 2,265,085 high-confidence variants (2.38 variants/kb) including 2,118,102 SNPs, 63,050 insertions, and 83,933 deletions (Additional file 2: Fig. S17a,b). Admixture, phylogenetic, and principal component analyses (PCA) clustered 112 accessions into four co-ancestry subgroups with optimal *K* = 4 (Fig. 4c,d; Additional file 2: Fig. S18,19). *S. oleracea* dispersed from the Middle East into nine geographical regions with one branch in Asia and another in Europe and North America (Fig. 4a). The nucleotide diversities (*π*) of *S. oleracea* (0.48 ± 0.32 × 10^−3^) and *S. turkestanica* (0.43 ± 0.29 × 10^−3^) were closer to each other but twice that of *S. tetrandra* (0.12 ± 0.10 × 10^−3^), which could be an artifact of a small sample size, not representing the scope and extent of genetic diversity in *S. tetrandra* (Additional file 2: Fig. S17c; Additional file 3: Table S13). Although Tajima’s *D* (1.52 ± 0.77) was highest in *S. oleracea* (Additional file 2: Fig. S17d), LD decay showed similar patterns across these species (Additional file 2: Fig. S20). The heterozygosity rate of *S. oleracea* (0.16 ±0.06%) was higher than that of *S. turkestanica* but lower than that of *S. tetrandra* (Fig. 4b; Additional file 2: Fig. S21).

Demographic history analyses showed that the *S. oleracea* population arose ~0.509 Mya and that its effective population size (*Ne*) began to decrease ~10.87 Kya, reached a minimum at ~6.5 Kya, and recovered well ~4.8 Kya (Fig. 4e). Neither of the wild species exhibited this relatively recent *Ne* decline, although the *S. turkestanica* population appeared to have undergone bottlenecks during the Late Tarantian Age ~148–177 Kya and the Greenlandian Age ~18.97–26.51 Kya, as did *S. tetrandra* during the Calabrian to Chibanian ages at 1.04–1.32 Mya due to the effects of Quaternary glaciations.

### Selective sweeps and domestication of edibility traits in spinach

**Fig. 5:**
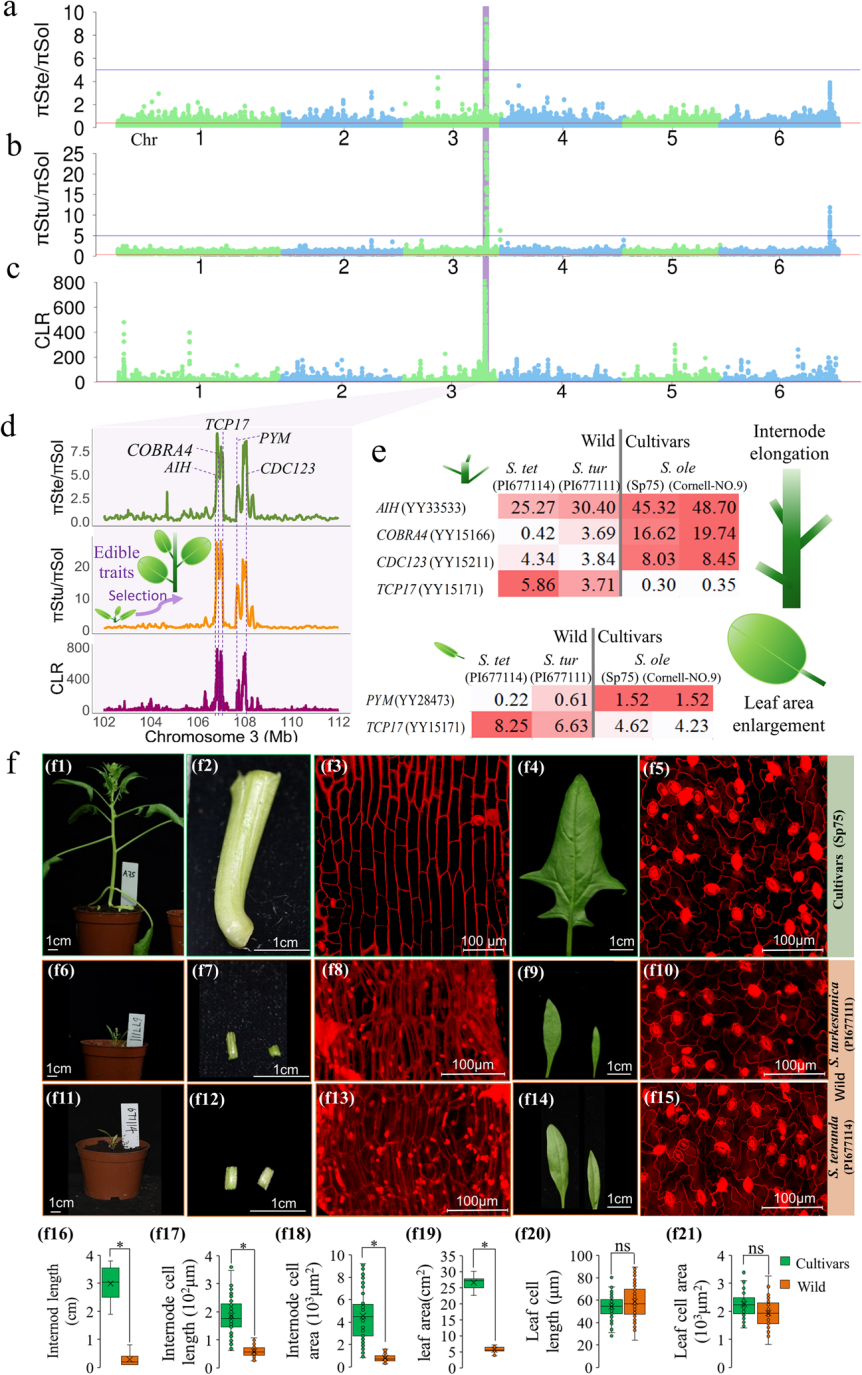
Selective sweeps in the *Spinacia* population. **a** The ratio of nucleotide diversity between wild *S. tetrandra* and cultivated *S. oleracea* (πSte/πSol) with 100-kb sliding window in 20-kb steps across the spinach YY genome. The blue solid line indicates the 1% cutoff outlier with significant selective sweep signals. **b** The ratio of nucleotide diversity between wild *S. turkestanica* and *S. oleracea* (πStu/πSol) in 100-kb sliding window in 20-kb steps. The blue solid line indicates the 1% cutoff outlier with significant selective sweep signals. **c** The composite likelihood ratio (CLR) with 20-kb grid size for the *S. oleracea* population. **d** A strong sweep signal at chromosome 3:106,112,216–108,343,948 bp harbors genes influencing several domesticated edibility traits including internode elongation and leaf area enlargement. **e** Expression (FPKM) of candidate genes for spinach domestication in internode tissue (top) and leaf tissue (bottom) compared between wild species (*S. turkestanica*: PI677111 and *S. tetranda*: PI677114) and cultivars (Sp75 and Cornell-9). **f** Comparison between cultivated *S. oleracea* (Sp75) and wild species (*S. turkestanica* PI677111 and *S. tetranda*: PI677114) phenotypes (f1, f6, f11), internode length (f2, f7, f12), internode cell length (f3, f8, f13), leaf size (f4, f9, f14), and leaf cell area (f5, f10, f15); comparison between cultivar *(*Sp75) and wild species for internode length measurement in cm (f16); internode cell length in μm (f17); internode cell area in μm^2^ (f18); leaf area in cm^2^ (f19); leaf cell length in μm (f20); and leaf cell area in μm^2^ (f21). Mean separation was performed using a *t*-test at *P* < 0.05. For wild species, average values of PI677111 and PI677114 were used for parameters in f16-f21 to draw comparison graphs

Using nucleotide diversity ratios between wild (πW) and cultivated (πC) species, together with composite likelihood ratio (CLR) statistics, we identified 284 high-confidence selective sweeps with an average of 9.17 kb (3.36–206.94 kb), representing 0.42% (2.61 Mb) of the genome and 0.632% (170) of annotated genes (Fig. 5a–c). The annotations of selected swept genes were enriched for reproductive and developmental processes, stimulus response, and catalytic activity, implying their possible roles in domestication (Additional file 2: Fig. S22a).

Plant height and leaf size are key edibility traits that have been improved during spinach domestication [31]. A strong sweep signal from Chr3:106,112,216–108,343,948 was enriched for annotations of genes involved in cell differentiation and polyamine metabolism whose homologs have been functionally characterized in *Arabidopsis* (Fig. 5a–c; Additional file 2: Fig. S22b). Three genes whose *Arabidopsis* homologs might be participating in either stem cell elongation via polyamine synthesis (*AIH*) [40] or cell expansion (*COBRA4*) [41] and cell division (*CDC123*) [42] showed cultivar-biased expression in the stem internode, while the *TCP17*, whose *Arabidopsis* homolog may negatively affect shoot morphogenesis [43], exhibited wild-biased expression in stem internode (Fig. 5d,e; Additional file 1: Supplementary Notes; Additional file 2: Fig. S23; Additional file 3: Table S14). *PYM*, whose *Arabidopsis* homolog may affect leaf primordium cell enlargement, showed cultivar-biased expression, while *TCP17*, which may negatively regulate leaf development, exhibited wild-biased expression in leaves [44, 45] (Fig. 5e; Additional file 2: Fig. S23). Moreover, morphological and cytological comparisons of *S. oleracea* cultivars to wild species showed relatively elongated internodes exhibiting longitudinal cell elongation and enlarged leaf area due to cell number proliferation, both of which might be regulated by these candidate genes (Additional file 1: Supplementary Notes; Fig. 5f).

### Genome-wide inter- and intraspecific introgression

Our chloroplast genome-based phylogeny constructed from 108 *de novo Spinacia* chloroplast assemblies differed from our nuclear genome variant-based phylogeny (Fig. 4d, Additional file 1: Supplementary Notes). Some accessions were more closely related to the wild species than to the rest of *S. oleracea* and vice versa. These conflicts between the nuclear and chloroplast phylogenies indicate a broad range of gene flow and hybridization events during spinach evolution and domestication. This observation was further supported by network analysis using SplitsTree [46] (Additional file 1: Supplementary Notes; Additional file 2: Fig. S24).

**Fig. 6:**
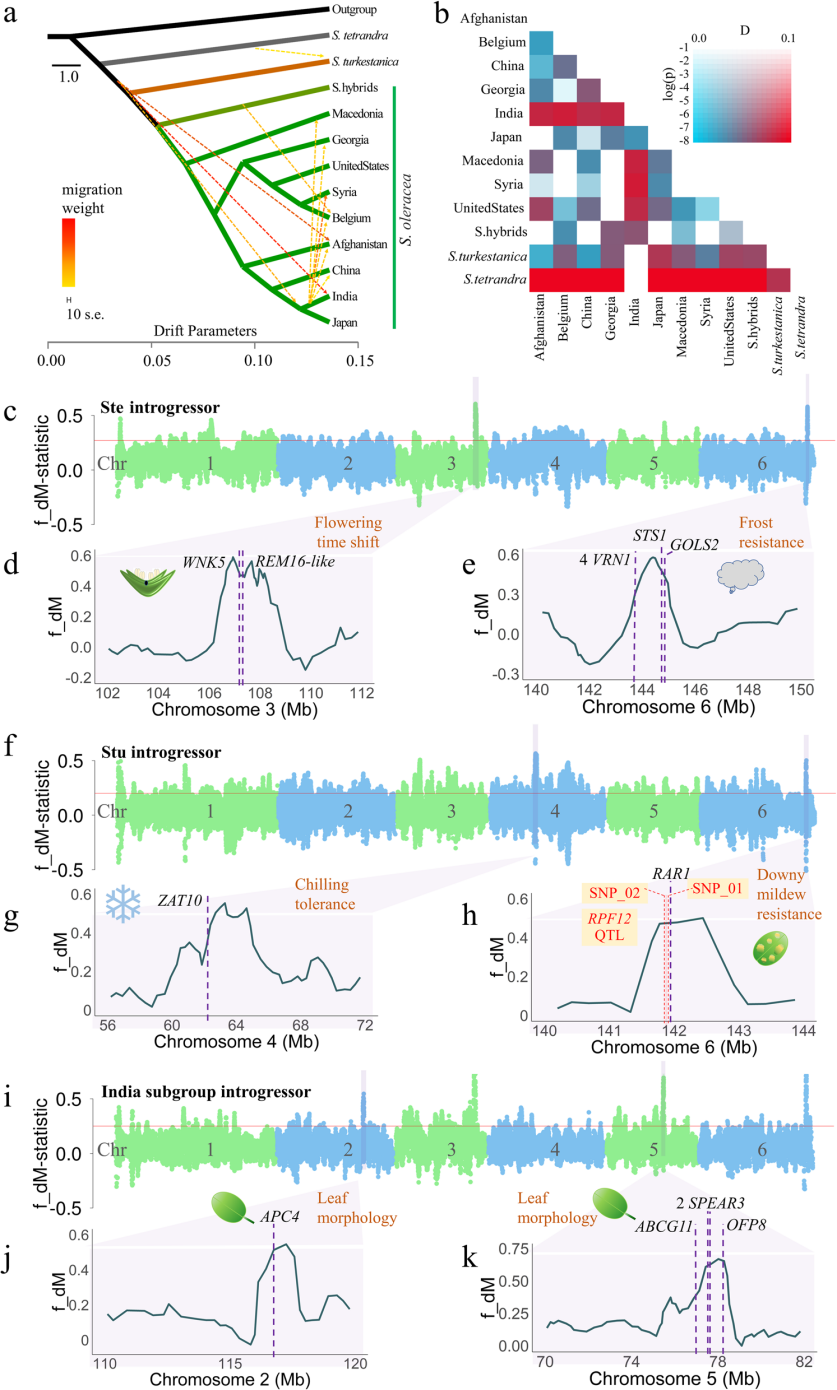
Genome-wide introgression in the *Spinacia* genome. **a** TreeMix analysis of nine geographical subpopulations of *S. oleracea* and two wild species *S. tetrandra* and *S. turkestanica*. **b** Patterson’s *D* statistic measurements of admixture among populations of *S. oleracea* in nine regions, hybrids and two wild species. **c** A modified *f(d)*-statistic (*fd_M*) with 1-kb window in 200-bp steps is plotted along the YY genome with *S. tetrandra* as the introgression donor. Each dot represents a 1-kb window, and the red horizontal line represents top 5% cutoff (the same below). The two strongest signals of introgression on spinach chromosomes 3 and 6 are plotted with *fd_M* to display gene introgression related to the flowering time shift (**d**) and frost resistance (**e**) respectively. **f** The *fd_M* statistics plotted along the YY genome with *S. turkestanica* as the introgression donor. The two strongest signals of introgression on chromosomes 4 and 6 are plotted with *fd_M* to show the introgressed genes related to chilling tolerance (**g**) and downy mildew resistance (**h**), respectively. The known *RPF12* QTL for downy mildew resistance was plotted with two boundaries SNPs (SNP_01 and SNP_02) located 23 kb from the *RAR1* candidate for downy mildew resistance. **i** The *fd_M* statistics plotted along the YY genome with the India subgroup of *S. oleracea* as the introgression donor. The two strongest signals of introgression on chromosome 2 and 5 are plotted with *fd_M* in **j** and **k**, respectively

TreeMix analysis was performed to detect potential gene flow among nine geographical regions of *S. oleracea* and wild species (Fig. 6a)*.* Eleven migration events were detected with optimal *m* value, including one from *S. tetrandra* to *S. turkestanica*, three from *S. turkestanica* to *S. oleracea*, and seven among cultivation regions (six from India to other regions) (Fig. 6a; Additional file 2: Fig. S25). The results indicated that spinach has undergone broad inter- and intraspecific introgression.

Patterson’s *D* statistics in ABBA-BABA tests showed strong potential introgression signals from two wild species and the India subgroup (Fig. 6b; Additional file 3: Table S15). The modified ƒ(d) statistics *fd_M* detected 26.17 Mb, 109.956 Mb, and 11.86 Mb fragments that might have been introgressed into *S. oleracea* from *S. tetrandra*, *S. turkestanica,* and the India subgroup, respectively (Fig. 6c–k; Additional file 3: Table S16). By exploiting information from six genomic regions with strong introgression signals (Fig. 6c–k), we quantified and distinguished introgression from ILS (incomplete lineage sorting) using the QuIBL (quantifying introgression via branch lengths) method. The inferred probability of the model with non-ILS topologies has significantly lower BIC scores (with a strict delta BIC (dBIC) cutoff > 30) than does the model with ILS topologies, indicating that the shared evolutionary histories of these six regions of the *Spinacia* genome were due to introgression rather than ILS (Additional file 2: Fig. S26). The annotations of the genes in these regions were enriched in functions such as response to vernalization, oomycetes, and reproductive process, which implied potential adaptive functions or resistance introduced by genomic introgression due to hybridization (Additional file 2: Fig. S27).

For example, introgressed fragments on chromosome 3 (106,095,210–108,631,646 bp) from wild *S. tetrandra* include the genes *WNK5* (YY15162) and *REM16-like* (YY28472), whose *Arabidopsis* homologs might be regulating flowering time by modulating the photoperiod pathway [47] or directly activating promoters *SOC1* and *FT* (Fig. 6c,d) [48].

Interspecific hybridization is also responsible for the increase in spinach chilling tolerance or frost resistance [31]. The annotation of the region containing a strong introgression signal on chromosome 6 (143,219,244–145,427,146 bp) from wild *S. tetrandra* donors was enriched for gene ontology terms related to cold and frost resistance, including *STS1* (YY33589) [49] and *GOLS2* (YY09096) [50], whose *Arabidopsis* homologs might be involved in the biosynthesis of raffinose family oligosaccharides (RFOs), which act as cryoprotectants in frost-hardy plants (Fig. 6c,e; Additional file 3: Table S16; Additional file 2: Fig. S27b). Additionally, *Arabidopsis* homologs genes related to sensitivity to freezing (*CP12-2*) and vernalization (four duplicated *VRN1* paralogs) were also included in this region (Additional file 3: Table S16). Moreover, the *ZAT10-like* (YY37988) on chromosome 4 was introgressed from *S. turkestanica,* whose *Arabidopsis* homolog might enhance tolerance to osmotic stress and also contribute to cold tolerance (Fig. 6f,g) [51].

Interspecific introgression from wild species has also contributed to pathogen resistance in spinach [31]. By searching sequences of a newly identified QTL locus *RPF12* that contributes resistance to *Peronospora farinosa* races 9–15 in spinach [52], we confirmed its location on chromosome 6 (141920113–141995017), exactly overlapping a strong signal on chromosome 6 (141185447-143292111) of an introgression from wild *S. turkestanica* (Fig. 6f,h; Additional file 3: Table S16). A *RAR1* homolog (YY27267) located 23 kb downstream of this QTL might be the causal gene for downy mildew resistance in modern spinach. *RAR1* genes encode a cysteine- and histidine-rich domain-containing protein specifically required for downy mildew resistance, conferred by multiple R genes recognizing oomycete pathogens [53]. Moreover, homologs of other fungal pathogen resistance genes such as *HIR4, PHOS32,* and *RGA3* that were identified within or near the region introgressed from either *S. turkestanica* or *S. tetrandra* might also coordinate with *RAR1* in resistance to downy mildew or other pathogens (Additional file 3: Table S16) in spinach.

Edibility traits such as larger, flat, and thicker leaves might have been improved during the evolutionary history of spinach by intraspecific introgression [31]. Chromosome 5 contains a strong signal (76,455,899-78,871,174) introgressed from India group enriched for annotations of genes involved in regulation of cellular metabolism and contains homologs of genes (*OFP8, SPEAR3*, *ABCG11*) related to leaf morphogenesis (Fig. 6i,k; Additional file 3: Table S16; Additional file 2: Fig. S27e). In *Arabidopsis*, the expression of homologs of *OFP8* (*YY19308*) result in flat, thick, blue-green leaves [54]. The *Arabidopsis* homolog *SPEAR3* (*YY19401, YY19406*) is a transcriptional regulator whose overexpression leads to serrated leaves [55]. The ATP binding cassette (ABC) transporter homolog *ABCG11* (YY37993) participates in the expanding leaf vascular system and epidermis [56]. Spinach chromosome 2 (115,436,812–118,274,583) contains a homolog of the gene *APC4* (YY37240) and its *Arabidopsis* ortholog increases mature leaf size by altering vein patterning (Fig. 6i,j; Additional file 3: Table S16) [57].

## Discussion

In order to better understand sex chromosome evolution and the genomic architecture of the sex-determining region (SDR) on the Y chromosome in spinach, we sequenced and assembled the genome of supermale YY spinach individuals (derived from an androdioecious population) using PacBio long reads with Hi-C technology, and obtained accurate X and Y haplotypes by comparison to the reference female XX genome assembly. Although the contig assembly was fragmented due to highly repetitive sequences (74.00%), this YY genome assembly improved anchor rate of sequences to the chromosomes to 99.79%, substantially higher than published draft genomes [28]. Further, GWAS of X and Y haplotypes from natural populations, genomic analyses of female and male populations, and F_1_ linkage maps together delimited a ~17.42 Mb SDR on spinach chromosome 1 (Y chromosome).

Genomic insertions, particularly the retrotransposon burst, contributed significantly to expansion of the SDR compared to its X counterpart [10, 12]. There is no large size expansion of spinach SDR (17.42 Mb) comparing with its X counterpart (16.23 Mb) (Fig. 2a,b). This is likely due to incomplete assembly of the SDR, as some fragmented Y-specific sequences may not have been anchored due to the highly repetitive nature of this genome and the reliance on the X counterpart to identify SDR contigs (Fig. 2a,b; Additional file 3: Table S5). Besides the substantial genome-wide accumulation of LTR-RTs, the LTR-RT bursts that occurred exclusively in the SDR and the occurrence of the inversion 1 expedites the extension of recombination suppression within a short evolutionary period (Fig. 2f,g). The inversion 1 which dated around 1.98 Mya, together with pronounced variations and mutations been accumulated, implies its role in early stages of sex chromosome evolution (Fig. 2a,b). The smaller inversion 2 in stratum 2 occurred at about the same time compared with inversion 1, which is unusual, and has first been detected in the spinach sex chromosome. The different *Ka*/*Ks* ratios from two strata were the products of different time points of two inversions and compounded by variable gene functions under different selection pressures (Fig. 2e). The large SDR in spinach is thus likely the product of suppressed recombination caused by inversions and insertions, as well as expansion due to a retrotransposon burst.

Given the independent origins of dioecy across numerous lineages in angiosperms, different sex determination genes regulate male and female sterility in unrelated dioecious species [2, 3, 10, 11, 14, 17, 21]. One possible consequence of sex chromosome evolution is the genes with male-specific function that evolved and accumulated in SDR of Y chromosome [2, 4]. This scenario is supported by two Y-specific *NRT1*/*PTR* 6.4 and *EIF3-subunitA* genes flanking the oldest inversion (possibly related to initiation of sex chromosome evolution) as strong candidates for sex determination or differentiation (Figs. 2a; 3a,b). Expression profiling of male floral buds indicates that YY-specific *NRT1*/*PTR 6.4* and *EIF3-subunitA* exhibit transcript expression synchronized with that of genes related to hormones, stamen identity, and fertility (Fig. 3c; Additional file 2: Fig. S15e; Additional file 3: Table S12). *NRT1.1*/*NPF6.3, AIT3*/*NPF4.*1, and *AtGTR1*/*NPF2.10*, which belong to the same transporter family, have reported roles in the transport of auxin [58], ABA/GA (abscisic acid and gibberellic acid) [34], and GA/JA (gibberellic acid and jasmonic acid) [34], respectively. Further, a *GTR1* (NRT1 family member) knockdown mutant has defective stamens due to lack of JA and GA transport [34]. In spinach, gibberellin regulates B-class genes *AP3* and *PI* that encode main masculinizing factors, and their silencing causes male spinach to transform stamens into carpels [59, 60]. In the present study, GA-signaling genes showed sex-biased expression from stage 2 to stage 3, while JA-biosynthesis and B-class genes were coexpressed with Y-specific *NRT1* and *EIF3* in stage 1 (S1). This result suggests that both JA and GA might be transported by *NRT1* either synergistically or through independent pathways regulating spinach stamen identity. The *Arabidopsis eIF3e-Tp* mutant with reduced *AP3* and *PI* expression and defective male gametogenesis similar to those of the ufo mutant [61] also implies an upstream function for YY-specific *EIF3.* However, the non-significant relative expression of *EIF3* at stage 1 of female and male spinach flowers makes it a less likely candidate than *NRT1* for the sex determination gene (Additional file 2: Fig. S16). Further, differential regulation of the floral stem cell termination pathway *AG-KNU-WUS* (opposed by B-class genes [62]) and strong negative correlation between the expression of *NRT1*/*PI* and the meristem termination and gynoecium development-related gene *CRC* suggest that B-class genes might function as an important intermediate during androecium/gynoecium differentiation. In rice, the *CRC* homolog *DROOPING LEAF* mutation causes loss or complete transformation of carpels into stamens by spatial expansion of function of the *AP3* ortholog *SPW1* in 4th whorl [63]. Okazaki et al. reported that increased dosage of a single sex-determining factor results in a shift towards maleness by regulating the proportion of pistillate and staminate flowers [64]. Our data presented herein provides some evidence for such a single-factor model, in which the presence/absence of Y-specific *NRT1* gene expression might regulate two independent pathways like *NRT1-JA*/*GA-PI* and *NRT1-PI-CRC*/*KNU*/*WUS* for stamen and carpel initiation, respectively (Fig. 3d).

The population structure and diversity of spinach germplasm have been explored using transcriptomic data [28]. However, little is known about spinach evolution and domestication at the genomic scale. Our analyses of 112 resequenced genomes have now shed some light on the genetic diversity and domestication history of spinach. The heterozygosity rate of *S. oleracea* between two wild relatives indicated genetic improvement after selective fixation during domestication [65]. Like maize, *S. oleracea* underwent a population bottleneck ~10.87 Kya [66], and thus its domestication started 7000 years earlier than indicated in archeological record at ~3 Kya [29] (Fig. 4e). However, the time of the *Ne* recovery at ~0.48 Kya indicates a protracted 4300-years pre-domestication, as took place in African rice [67]. Our whole-genome identification of domestication signatures revealed a strong selection signal at chromosome 3 that contains several genes associated with cell elongation, division, and shoot morphology that could affect domestication-related traits such as leaf area and plant length and indicate the role of humans in selection for edibility traits in spinach [31].

Worldwide expansion of spinach cultivation accompanied by disease and environmental pressures has led breeders to broaden the genetic base of this crop by introgressive hybridization from wild sibling species to create modern spinach cultivars [31]. Hybridization history, gene flow, and inter- and intraspecific introgression in spinach have been detected by testing conflicts between the phylogenies of its nuclear and chloroplast genomes, using TreeMix analyses, and performing ABBA-BABA tests with a large collection of cultivars and wild species (Figs. 4d; 6a–k).

Downy mildew (DM) is the most destructive disease affecting commercial production of spinach worldwide [31]. Genome-wide scans for introgression identified a strong signal on chromosome 6 located near a likely DM resistance gene *RAR1* from wild *S. turkestanica* that could be the causal factor conferring downy mildew resistance in modern spinach cultivars [52, 53]. In addition, several introgression signals associated with flowering time regulators *WNK5* and *REM16-like* [47, 48], frost or chilling tolerance (*STS1*, *ZAT10*, and four duplicated *VRN1* paralogs) [68] came either from two wild relatives or germplasm from the India subgroup. Further, leaf morphogenesis regulators that regulate the development of leaf edges (*SPEAR3*) [55], flat/thick leaf blades (*OFP8*) [54], expanding vasculature (*ABCG11*) [56], leaf area (e.g., *PYM*) [45], or leaf vein patterns (*APC4*) [57] were identified from chromosomes 2 through 6, respectively, suggesting the importance of genomic introgression for the domestication and improvement of traits in spinach such as acclimation, edibility, and delayed bolting. These candidate genes of domestication and improvement could be potential targets for molecular breeding and gene editing in spinach (Additional file 2: Fig. S28).

## Conclusion

Our study affords the first high-quality chromosome-scale spinach YY genome assembly derived from long-read sequencing, along with the phased Y chromosome and a ~17.42 Mb SDR with two genomic insertions and inversions been defined. This resource paved the avenue towards understanding evolutionary landscapes of spinach sex chromosomes and will lay the foundation for studying sex determination mechanism across angiosperms. A Y-specific candidate *NRT1/PTR 6.4* which might control stamen initiation/carpel suppression was proposed as a single sex determination factor. Further, comprehensive population genomic analyses based on resequencing genomes provide insights into spinach domestication, introgression, and genetic basis of important agronomic traits. The high-quality reference genome and population genomic resources generated in this study are of great value for future biological studies and will undoubtedly facilitate spinach improvement.

## Methods

### Part 1. Genome sequencing, assembly, and annotation

#### Genome assembly and annotation

Supermale YY individuals were obtained from the USDA androdioecious XY “Cornell-NO. 9” (PI 217425) accession [23]. *De novo* assembly of the YY genome was performed as described in Additional file 1: Supplementary Notes. Briefly, we obtained ~67× coverage of subreads from the PacBio RSII platform and ~43× coverage of short reads from the Illumina HiSeq X Ten platform. The initial YY contig assembly was performed with PacBio long reads using CANU [69] and further polished with short reads using Pilon [70]. Hi-C libraries were created to correct polished contig sequences. The paired-end Hi-C reads were uniquely mapped to the draft assembly and mis-joined contigs were corrected by detecting abrupt long-range contact patterns using 3D-DNA [71]. The initial contig assembly of the spinach cultivar “Viroflay” genome (XX) [32] was adopted and optimized with Hi-C data using the ALL-Hi-C pipeline [72] to generate a chromosome-scale reference for anchoring the YY genome. Because direct grouping of the YY Hi-C contigs would generate large artifactual chimeras due to noisy Hi-C signals caused by short Hi-C reads ambiguously mapped to repetitive sequences, we first grouped the Hi-C-corrected YY contigs according to the complete chromosome-level XX assembly using Ragoo [33] and then linked them into chromosomes using the ALLHiC pipeline (Additional file 1: Supplementary Notes).

Gene annotations for both the YY and XX genome assemblies were performed using the GETA pipeline, https://github.com/chenlianfu/geta/ (GPL-3.0 License), by integrating information for homologous proteins, RNA-seq assembled transcripts, and the results of ab initio gene predictors (Additional file 1: Supplementary Notes).

#### Collinearity analysis of the spinach YY and XX genomes

MCScan v3.23, https://github.com/tanghaibao/jcvi/wiki/MCscan-(Python-version)/ (BSD-2-Clause License), was used to detect collinearity blocks between gene models in the YY and XX genomes with a C-score cutoff of 0.65. We defined homology blocks that did not belong to the syntenic backbone as regions containing potential rearrangements.

#### Comparative analysis of the evolution of repeat elements among six genera in the Amaranthaceae

To estimate genome-wide LTR burst patterns in the five Amaranthaceae congeners (*Beta*, *Bassia*, *Suaeda*, *Chenopodium*, and *Amaranthus*) and in the YY and XX genomes *Spinacia*, we annotated repeat elements using a pipeline described in Additional file 1: Supplementary Notes. We further calculated and visualized the Kimura substitution rate distribution among repeat classes and their percentages of genome size using createRepeatLandscape.pl scripts, https://github.com/rmhubley/RepeatMasker/blob/master/util/createRepeatLandscape.pl/ (Open Software License v. 2.1). Finally, we present the repeats landscape of each genome based on a Maximum Likelihood tree of the six genera generated using single-copy orthologous genes in RaxML-ng, https://github.com/amkozlov/raxml-ng (AGPL-3.0 License), with an optimal JTT+I+G+F substitution model chosen by ProTest v3.2, https://github.com/ddarriba/prottest3 (GPL-2.0 License).

### Part 2. Sex chromosome analyses

#### Identification of the spinach sex determination region (SDR)

Genetic maps of F_1_ “Viroflay” × “Cornell-NO. 9” were constructed with bin markers derived from resequencing SNPs using the YY genome as a reference, as described in previous studies [27]. Initially, SNPs were filtered according to the criteria MAF > 0.05, minDP=3, maxDP=35, and minQ > 20 in VCFtools [73] (Additional file 1: Supplementary Notes; and Additional file 2: Fig. S4a). Co-segregating SNPs were merged into consensus bins based on majority rules and manual correction. Bin markers with all heterozygous alleles for female parents and all homozygous alleles for male parents were chosen to build the genetic map of “Viroflay” (female), which is essentially an inverted homozygous and heterozygous pattern for the genetic map of “Cornell-NO.9” (male). Both genetic maps were built using Lep-Map3 maximum likelihood algorithm [74].

To obtain high-quality variants for defining the sex determination region (SDR), we applied repeat-masked YY genome for reads mapping, and retained the unique mapped reads for variants calling in GATK pipeline [75] (Additional file 1: Supplementary Notes). To filter low-quality variants for identifying SDR, we applied a combined criteria including read depth (minDP, maxDP), minimum quality (minQ), quality by depth (QD), and genotype quality (GQ) (see details in Additional file 1: Supplementary Notes; and Additional file 2: Fig. S4a) to generate 177,414 high-quality SNPs. The contigs contain at least four sex co-segregation SNPs were selected as the sex co-segregation contigs. To exclude potential false positive and artifacts of genetic maps, the natural population using resequenced genomes were used to generate 4,844,193 high-quality SNPs after filtering (see filtering details in Additional file 1: Supplementary Notes; and Additional file 2: Fig. S4b). A genome-wide association study (GWAS) of 26 female and 44 male accessions (Additional file 3: Table S13 lists sexual phenotypes) was performed to detect regions associated with the two sex phenotypes using the EMMAX (efficient mixed-model association (EMMA) eXpedited) method [76]. EMMAX was conducted with parameters *d* = 10, *v* = verbose mode to generate a kinship matrix, and association analysis was implemented with population structure as the covariate. Besides, sites associated with high genetic differentiation (*Fst*) score between female and male resequenced genomes, and regions along with diverged Tajima’s *D* value between two sexes was calculated in VCFtools [73]

The boundaries of SDR in spinach were defined by taking overlapped regions derived from independent clues which include sex co-segregation contigs, GWAS mapping, male-specific SNPs, and *Fst* and Tajima’s *D* values between two sexes. We screened the region associated with the top 1% density of sex co-segregation contigs in each 100-kb window, the top score in GWAS (−log_10_(*P*) ≥ 6), the top 1% density of male-specific SNPs in a 20-kb window, the top 5% *Fst* statistics in each 1000-kb window, and the top 5% male/female ratio of Tajima’s *D* value in a 200-kb window. The overlapped regions derived from these cutoffs were retrieved as cross-validation to identify borders of SDR. The terminals of two contigs flanking this region were defined as two boundaries of SDR.

#### Genomic analysis of the SDR and its X counterpart

Mummer 4.0 pipeline, https://github.com/mummer4/mummer/ (The Artistic License 2.0), was used to process XX and YY genomic sequences (minimum length for match = 2000 bp) to characterize genomic variations between them. We defined the X counterpart of the SDR based on a microsynteny analysis generated by Mummer. Genome-wide syntenic genes and the presence of structural variations (SVs) within the SDR such as INDELs or inversions were further identified using MCscan pipelines (cs-score ≥ 0.65). We further statistically characterized the mapping depth of PacBio long reads from each 6-kb window across insertions, inversions, and the edges of these regions flanking neighboring collinear regions to confirm assembly quality of regions associated with these structural variants (SVs). Gene within the SDR and its X counterpart were retrieved to identify X/Y gene pairs and sex-specific genes. We also characterized the distribution of genes, repetitive sequences, and chromosomal rearrangements within these two sex-linked regions.

#### Identification of X- and Y-linked gene pairs, sex-specific genes, and Y-specific candidate sex determinant genes

To resolve discrepancies in gene annotation between the X and Y genomes, we identified gene pairs and sex-specific genes by combining MCscan and blastN analyses. Gene models from female- and male-specific annotations were used to build synteny blocks in MCScan. Genes in synteny blocks on either the X or Y genome with no corresponding partner were further screened by testing for presence/absence variations (PAVs) on the other genome using blastN with identities > 90 and coverage > 20. Those genes from X counterpart with no match in the Y-SDR were classified as X-specific genes, and Y-encoded gene models missing from the X counterpart were classified as Y-specific genes. Hits from blastN searches with collinear location of their counterparts were classified as X- and Y-linked gene pairs. Hits that had no collinear location but were present in the SDR or X counterpart were classified as non-sex-specific genes.

To further narrow down candidates for Y-encoded sex determinants after detecting Y-specific blocks using MCscan, we further integrated two additional methods. (1) We first performed blastN searches against the XX genome using both sequences and gene models from the Y chromosome as the queries with identities > 90 and coverage > 20. The intersecting genes from both searches were then treated as Y chromosome-specific genes. (2) We further performed K-mer analysis (Additional file 1: Supplementary Notes) to retrieve the Y-specific contigs from the contig assembly of the YY genome assembled from 43× YY Illumina reads with the XX chromosome assembly as the reference genome.

#### Estimation of the divergence of X- and Y-linked gene pairs between the SDR and its X counterpart

To estimate the degree of divergence of gene pairs across the SDR, we chose X- and Y-linked gene pairs with conserved structure and compared gene identities via the numbers of mutations between those X- and Y-linked genes. Also, we dated the divergence time of the X and Y chromosome as in previous studies [4, 10] using a molecular clock (*r* = 2.8e−9) based on fossil records from the Amaranthaceae family [77]. Substitution of X- and Y-linked gene pairs in the SDR (cutoffs: > 85% identities, coverage > 60%) were analyzed using the easy_KaKs pipeline (https://github.com/tangerzhang/FAFU-cgb/blob/master/easy_KaKs) and the divergence time of the sex chromosomes was further calculated as *T = Ks/2r* with a substitution rate *r* = 2.8e−9.

#### Estimation of LTR-RT insertion times in the spinach genome

We performed annotation of the LTR-RTs (Additional file 1: Supplementary Notes) using the LTR_retriever pipeline [78] to study the divergence time of LTR-RTs using the formula *T* = *K*/*2r*, with the substitution rate *r* = 2.8e−9. Further, we compared LTR-RT insertion times for two genomic inversions, two insertions, and collinear regions between the SDR and its X counterpart, to estimate LTR-RTs burst times in the SDR and its X counterpart, the sex chromosome, and the entire YY and XX genomes.

### Part 3. Transcriptome analysis

#### Analyses of the transcriptomes of female and male flowers at five stages using RNA-seq

Three biological replicates of male (M) and female (F) flowers at five different developmental stages (S1–S5) were collected [79] (Additional file 1: Supplementary Notes; Additional file 2: Fig. S9,10a). Total RNA was extracted from samples using a RNeasy Plant Mini Kit (QIAGEN China Co., Ltd.), and libraries were constructed using an Ultra RNA Library Prep Kit (#E7770L, New England Biolabs, Ipswich, MA). High-throughput sequencing of indexed libraries to obtain 150-nt paired-end reads was performed on the Illumina HiSeq 2500 system. After removing low-quality reads using Trimmomatic [80], clean reads were mapped to the reference YY genome using STAR aligner [81]. Mapping reads referring to each transcript were assembled and FPKM values were calculated using StringTie [82]. For DEG analysis, expression data was used to calculate table counts with the script “prepDE.py” and DEG were calculated using DESeq2 in the R Bioconductor package [83] using the parameters log2FC > 1 for genes with increased transcript abundance and log2FC <− 1 for genes with decreased transcript abundance and a threshold *P*-value ≤ 0.05. Comparisons were made at each corresponding stage (S1 through S4) between the two sex types (F, female; M, male) (i.e., FS1 compared to MS1).

#### Construction of coexpression network linking to candidate sex determinants

Data sets of DEGs at stage 1 (S1) and stages 1 through 5 (S1–S5) were chosen individually and subjected to analysis using the WGCNA package in R [84]. WGCNA network construction and module detection were conducted using an unsigned type of topological overlap matrix (TOM), with parameters soft power = 5, minModuleSize = 30, and mergeCutHeight = 0.25. Co-expressed genes in the male-module of stage 1 (MS1) related to the formation of male floral organs and also directly linked with Y-specific candidate sex determinants were visualized using the VisANT program [85]. The final network was illustrated using the igraph package [86].

### Part 4. Resequencing and population genomics analysis

#### Sample collection, sequencing, and variants calling

Genomic DNA of 112 accessions (Additional file 3: Table S13) from three *Spinacia* species was extracted from leaf tissue using a Qiagen DNeasy Plant Mini Kit. Libraries were constructed for 150-bp paired-end sequencing using the NEBNext® Ultra DNA Library Prep Kit and sequenced using the Illumina HiSeq 2500 platform. After trimming raw reads using Trimmomatic [80], clean reads were mapped to the YY genome using Bowtie2 [87]. Variant calling was performed using the GATK pipeline with the HaplotypeCaller model [75]. A total of 2,265,085 SNPs and InDels remained after filtering out variants with DP < 2 or DP > 60, minQ < 20, > 20% maximum missing rate, and minor allele frequency (MAF) < 5%. The remaining variants were further annotated and classified as SNPs, Indels, other synonymous or nonsynonymous variants, intronic variants, and those located in the upstream or downstream regions of genes or in intergenic regions using SnpEff v3.6c [88].

#### Analyses of genomic diversity, PCA, phylogeny, and population structure

The SNP densities, nucleotide diversity (*π*), and Tajima’s *D* were calculated in 50-kb sliding window with 10-kb steps in VCFtools [73] using the filtered set of 2,265,085 variants. Linkage disequilibrium (LD) decay was calculated using PopLDdecay, https://github.com/BGI-shenzhen/PopLDdecay/ (MIT License). We excluded the sex chromosome for downstream analyses and used GCTA [89] to perform a principal component analysis (PCA). We used VCFtools and PLINK [90] to convert the VCF file into Plink binary files, then used the top two principal components to assign the 112 spinach accessions to PCA clusters. A total of 4,976,299 SNPs that were either bi-allelic or polymorphic were selected to reconstruct a phylogeny of these accessions using SNPhylo [91]. ADMIXTUR [92] was used to infer ancestral population stratification with the optimal population size chosen from *K* = 1 through 10 as that with least error after resampling for cross-validation.

#### Estimation of demography history

The site frequency spectra (SFS) of cultivated *S. oleracea* compared with two wild species were estimated using ANGSD [93]. We used the Expectation Maximization (EM) algorithm to compute a maximum likelihood estimate of the folded SFS from filtered BAM files, then used its output to estimate population demographic history by Stairway plots [94] with 200 bootstrap iterations. Because of the variation in the molecular substitution rate within the Amaranthaceae family [77], we used a range of molecular clocks (*μ* = 4e−9, *μ* = 6e−9, or *μ* = 8e−9) as mutation rates. Because *S. oleracea* is an annual plant, we used generation time of one year (gt = 1).

#### Detection of domestication selection

Selective sweeps were detected according to the ratio of genetic diversity between wild and cultivated (πW/πC) species, excluding the highly admixed accessions, in 100-kb sliding window with 20-kb steps. The top 1% of πW/πC statistics including 2-kb flanking regions were defined as the candidate sweep regions. Further, SweeD [95] was also used to detect the absolute selective sweeps using a grid size of 20 kb. The CLR (composite likelihood ratio) statistic was used as the criteria in SweeD analysis to detect significant deviations from the neutral site frequency spectrum (SFS). The top 1% of both statistics, πW/πC and CLR, with 2-kb flanking regions were regarded as the candidate sweep regions, which were then merged if outlier regions overlapped at a distance of 4 kb. Genes overlapping the swept regions were treated as genes putatively under selection.

#### De novo assembly of the chloroplast genome testing conflicts of cyto-nuclear phylogeny

A total of 108 resequencing samples from 112 resequenced accessions of three species were chosen for de novo assembly of each chloroplast genome (Additional file 1: Supplementary Notes). The phylogenetic relationships among 108 *Spinacia* accessions were constructed based on 108 chloroplast genomes using IQ-tree [96] with 10,000 bootstrap replicates. All of the sequences were aligned using HomBlocks [97] and then were pruned using BMGE tools [98]. The best substitution model K3Pu + F + I was chosen according to BIC criteria using IQ-tree. The chloroplast phylogenetic tree was then compared with the phylogenetic tree constructed using nuclear genome to detect conflicts between the evolution of the chloroplast and nuclear genomes using the ggtree package in R version 3.6.3, https://www.r-project.org/ (GNU General Public License).

#### Detection of gene flow and migration events among spinach cultivars and wild species

Gene flow and migration events among *S. oleracea* cultivar groups and its wild sibling species *S. turkestanica* and *S. tetrandra* were modeled in TreeMix v.1.12 [99]. Admixture trees were built using the two *S. tetrandra* accessions as the outgroup. We allowed m = 0 to 20 migration events. The optimal number of migration events was estimated using log-likelihood tests.

#### Detection of genome-wide introgression in spinach

Patterson’s *D* statistic [100] was used to examine whether each of nine geographical subgroups of *S. oleracea* shared more alleles with the wild species *S. turkestanica* and *S.tetrandra* than with other subgroups. The *D* statistic (ABBA/BABA) was used to examine introgression site patterns with a tree topology for the four groups as [[[P1, P2], P3], O] in ANGSD [93]. Two accessions of *S. tetrandra* were used as the outgroups (O) to test whether two subgroups, P1 and P2, shared more alleles with a candidate introgression donor P3 than with O. *D* statistics for all trios of subgroups and wild species were calculated, and the standard error was calculated using a weighted block jackknife [100]. *D* statistics significantly differing from zero indicate introgression between P1 and P3 (*D* < 0) or between P2 and P3 (*D* > 0). Further, we used a modified *ƒ*(d) statistics (*fd_M*) [101, 102] to locate genome-wide introgressed loci using a 1-kb sliding window with 200-bp steps. The frequencies of the derived ABBA and BABA allele at each site in each P1, P2, P3, and O, where P1 and P2 represent nine cultivation regions of *S. oleracea*, were compared with allele distributions in putative donors (two wild species and cultivation regions) (*P*3), respectively. Windows with positive 95^th^ percentile outliers for modified *ƒ*(d) were chosen, and merged if overlapping, as the final introgressed regions.

To distinguish introgression from incomplete lineage sorting (ILS), QuIBL (quantifying introgression via branch lengths) [103] was used to verify the regions with strong potential introgression signals detected by *fd_M* statistics. QuIBL uses BIC (Bayesian information criterion) scores to evaluate the probability of a model with non-ILS topologies compared to that of a model with ILS topologies for each triplet of trees (with a strict cutoff of delta BIC, dBIC = BIC1st - BIC2st > 30 to indicate an extreme difference between the probabilities of the two models). The tree topologies of each potential introgression region were first generated using population variant data in RaxML-ng, https://github.com/amkozlov/raxml-ng (AGPL-3.0 License), with 1000 bootstrap simulations and an optimal PMB+G4 substitution model chosen using Modeltest-ng, https://github.com/ddarriba/modeltest (GPL-3.0 License). QuIBL was then performed with tree topologies of each potential introgression regions using an Expectation Maximization (EM) algorithm with the following parameters: numdistributions=2, likelihoodthresh=0.01, numsteps=50; radascentscalar=0.5.

## Supplementary Information

**Additional file 1.** Supplementary Notes [104–122].

**Additional file 2.** Supplementary figures. **Figure S1-S28**.

**Additional file 3.** Supplementary tables. **Table S1**. Sequencing information of “Cornell-NO.9” (YY) genome. **Table S2**. Comparison of contig assemblies among three genomes of *Spinacia* accessions. **Table S3**. Statistics of Hi-C mapping of “Cornell-NO.9” (YY) genome. **Table S4**. Assembly and annotation evaluation in *S. oleracea* cultivar “Cornell-NO.9” (YY) and “Viroflay” (XX) genomes. **Table S5**. TE annotation of *S. oleracea* cultivar “Cornell-NO. 9” (YY) and “Viroflay” (XX) genomes. **Table S6**. Summary of sequencing statistics and sex phenotypes (F: female; M: male) in F1 population. **Table S7**. Summary of genes identified from SDR (MSY) and X-counterpart. **Table S8**. Gene information of sex-determination region (SDR) of Y chromosome. **Table S9**. Gene information of X-counterpart of SDR in X chromosome. **Table S10**. Statistics summary of Pacbio reads mapping onto junction regions of structure variations within SDR. **Table S11**. SDR genes having sex-biased expression during flower development. **Table S12**. Expression of genes that are mentioned in proposed sex-determination/differentiation pathway. **Table S13**. Accessions of spinach *S. oleracea* and wild sibling species used for resequencing and population genomics as well as sex-chromosome analyses. **Table S14**. Details of genes in a swept region with strong selection signals on Chr3:106112216-108343948. **Table S15**. D statistic values of ABBA-BABA tests for different geographical regions of *S. oleracea* and wild species. **Table S16**. Introgression loci with strong signals from *S. tetrandra*, *S. turkestanica* and India cultivars respectively. **Table S17**. Primers used in qRT-PCR for validating the expression patterns of key candidate genes found in this study [123–132].

**Additional file 4.** Review history.

## Data Availability

The PacBio reads, Hi-C reads, Illumina reads of resequencing genomes and transcriptome data have been deposited in the NCBI database under BioProject number PRJNA724923 [133]. The genome assembly and gene annotation have been deposited in the Genome Warehouse (GWH) database with accession NOs. GWHBGBP00000000 and GWHBHEW00000000 at the BIG Data Center under BioProject number PRJCA004899 [134]. The published “Viroflay” (XX) PacBio genome assembly version *Spinacia oleracea* Spov3, used in this paper, is available at Phytozome Database [32, 135].
