# Supplementary material for: The spinach YY genome reveals sex chromosome evolution, domestication, and introgression history of the species
Source: Genome Biol. 2022 Mar 7;23:75. doi: 10.1186/s13059-022-02633-x (PMC8902716; doi:10.1186/s13059-022-02633-x)
Supplement: Supplementary file 1 — Additional file 1. Supplementary Notes [104–122]. [file 13059_2022_2633_MOESM1_ESM.pdf]

The spinach YY genome reveals sex chromosome evolution, domestication and introgression history of the species

Supplementary Notes

Part1. Genome sequencing, assembly, and annotation

Pacbio sequencing. Genomic DNA from supermale leaves was extracted using the CTAB method. Genomic DNA was fragmented by g-TUBE centrifuged, and treated with end-repair, adapter ligation, and exonuclease digestion (Pacific Biosciences). DNA fragments (~20 kb) were selected by BluePippin electrophoresis (Sage Sciences). DNA libraries were sequenced on the PacBio RS II platform (Pacific Biosciences) with the P6–C4 chemistry. A total of 135 SMRT cells were sequenced for Cornell-NO.9 (YY) which generated an approximate 66 Gb raw data (**Additional file 3: Table S1**).

Illumina sequencing. The genomic DNA of Cornell-NO. 9 (YY) was extracted using the CTAB method and sequenced in Illumina HiSeq X-ten platform with 150-bp reads length and 300-500 insert size with a final ~43Gb data.

Estimation of genome size of Cornell-NO. 9 (YY) individual. The genome size of the Cornell-NO. 9 (YY) was estimated by Illumina short reads for *K*-mer analysis using published PERL scripts Estimate_genome_size.pl (https://bioinformatics.uconn.edu/genome-size-estimation-tutorial/). Genome size could be determined by the total number of *K*-mers divided by the peak value of the *K*-mer distribution. The size of the haploid genome of Cornell-NO. 9 was estimated to be 849.1 Mb (**Additional file 2: Fig. S1**).

Contig assembly. Initially, entire Pacbio subreads were self-corrected using CANU version 1.7[69] with parameters correctedErrorRate=0.105 and corOutCoverage=80. Each round of assemblies was inspected through evaluation of N50s, assembled genome size, as well as complete/duplicated BUSCO ratio (**Additional file 3: Table S2**). The total length of the final contig assembly for Cornell-NO. 9 was 948 Mb sequences with N50 of 26 kb. The genome validity was further polished by Illumina short reads using Pilon program [70].

Hi-C sequencing. Fresh leaves of spinach plants were collected to fix with formaldehyde, and lysed. The cross-linked DNA was digested with *HindIII* enzyme over 12hr. Sticky ends were biotinylated and proximity-ligated to enrich the chimeric junctions, and then physically sheared to a size of 500-700 bp. The chimeric fragments of cross-linked long-distance physical interactions were constructed into Hi-C libraries for paired-end sequencing. A total of 270 million 150-bp paired-end reads (~100 ×coverage) were generated on Illumina HiSeq X ten platform. The Hi-C sequencing was assessed using HiC-Pro[107] and showed high-quality data (> 80% validate reads) (**Additional file 3: Table S3**).

Hi-C scaffolding and chromosome assembly. To order and anchor the contigs of XX genome continuity, the published contig assembly of the cultivar 'Viroflay' XX (female) genome by PacBio reads [32] **(Additional file 3: Table S4**) was adopted, re-anchored, and scaffolded based on initial grouping of a linkage map [32] using 100 × coverage of Hi-C reads to generate the chromosomal-scale XX assembly (**Additional file 3: Table S3**). For the YY genome, Hi-C reads were uniquely mapped to the contig assemblies, and reads within 500 bp regions of *HindIII* restriction sites were retained for further analysis. Mis-joined contigs were corrected by detecting abrupt long-range contact patterns using the 3D-DNA pipeline [71]. Since directly using HiC grouping will generate chimeric large groups due to

noisy Hi-C signals, which caused by short Hi-C reads are prone to unambiguous mapping against repetitive sequences. The Hi-C corrected contigs of YY assembly were then firstly grouped using a reference-guided strategy based on XX chromosomal assembly groups using RaGOO [33], and further linked into six pseudo-chromosomes using 100 × coverage of Hi-C reads by the ALLHiC pipeline [72]. The accuracy of Hi-C based chromosome construction was evaluated by chromatin contact heatmap for both XX and YY genomes (**Additional file 2: Fig. S2**).

Validation of genome assembly. The genome completeness was assessed based on 1,375 conserved plant genes in BUSCO program [108] with default parameters. BUSCO reported 94.76% and 96.58% of completeness for YY and XX genomes, respectively (**Additional file 3: Table S2, S4**). Genome-wide analysis of chromatin interactions further validated the Hi-C scaffolding of the two genomes. The presence of prominent blocks along the diagonal indicates high-quality assembly, as contacts between nearby regions were generally more frequent than contacts between remote regions. Otherwise, mistakenly assembled genome usually exhibits incongruities such as contact-enriched blocks located away from the diagonal. The Hi-C heatmap revealed a well-organized interaction contact pattern along the diagonals within each pseudo-chromosome and confirm the quality of both assemblies from XX and YY genomes (**Additional file 2: Fig. S2**).

Gene annotation. The same annotation pipeline was used for both YY and XX genomes. Genome annotations were based on *ab initio* gene predictions, transcript evidence, and homologous protein evidence, all of which could be implemented in the GETA pipeline, https://github.com/chenlianfu/geta (GPL-3.0 License), an automatic genome-wide annotation tool (GWAT). GETA predicted gene models based on three types of evidences, including *ab initio* gene prediction, homologous proteins, and transcriptome data. To achieve this, GETA incorporates Augustus [109], Trimmomatic [80], HiSAT2 [110] and Genewise [111, 112]. After genome mask, RNA-seq data were first trimmed by Trimmomatic and further aligned against the reference genome using HiSAT2 [80,110]. To predict the reliable introns and optimize transcripts, GETA pipeline calculated coverage thresholds based on the sequencing depth for each alignment regions and filtered transcripts below the coverage cutoff. The remaining high-quality transcripts were subject to ORF prediction in TransDecoder, https://github.com/TransDecoder/TransDecoder/ (The Broad Institute, Inc. 2012). Based on the intron and exon structures predicted above, the gene models were then iteratively trained using augustus until best score obtained. Homologous proteins, including *Beta vulgaris* subsp. *Vulgaris, Oryza sativa Japonica, Arabidopsis thaliana, Chenopodium quinoa, Spinacia oleracea, Solanum lycopersicum, Gossypium raimondii, Brassica napus* downloaded from Phytozome v.12 (https://phytozome.jgi.doe.gov/pz/portal.html/) were used as subject to genewise for further protein identification. Pfam database was introduced to screen high-quality gene models and a final dataset of gene prediction including CDS, protein sequences and a GFF3 file locating gene position, was released based on evidences aforementioned using a PERL script (CombineGeneModeler) implemented in GETA.

The annotation results of YY genome were filtered if augustus support <70%, transcripts expression level (FPKM<=3) as well reads coverage of transcripts <=70% with at least one pfam domain and one blast hit. The annotation results of XX genome were filtered if augustus support <80%, transcripts expression level (FPKM<=3) as well reads coverage of transcripts <=80%, with at least one pfam domain and one blast hit. After filtering putative transposon-derived gene models, gene annotation was further assessed using BUSCO program [108, 113] with embryophyta_odb10 as testing database. A total of 26,910 and 27,759 gene models were annotated in YY and XX, respectively. BUSCO evaluation of the

gene annotations showed 90.25% and 96.95% completeness in YY and XX genomes, respectively (**Additional file 3: Table S2, S4**).

Functional annotation. The functional annotation was performed for both YY and XX genomes using blast against the NCBI NR database (ftp://ftp.ncbi.nih.gov/blast/db). The GO (Gene Ontology) and KEGG (Kyoto Encyclopedia of Genes and Genomes) annotations were performed in EggNOG-mapper [114] using the virNOG subclass embedded in eggNOG database (http://eggnog5.embl.de).

Repeat elements annotation. Initially, we customized a *de novo* repeat library of the genome using RepeatModeler (http://www.repeatmasker.org/RepeatModeler/), which can automatically execute two *de novo* repeat finding programs, including RECON (version 1.08, http://eddylab.org/software/recon/) and RepeatScout v1.0.5 [115]. The consensus TE sequences generated above were imported into RepeatMasker v4.05 (http://www.repeatmasker.org) to identify and cluster repetitive elements. Unknown TEs were further classified using TEclass v2.1.3 [116]. To identify tandem repeats within the genomes, the Tandem Repeat Finder (TRF) package v4.07 [117] was executed with the modified parameters of “1 1 2 80 5 200 2000 –d -h” to find high order repeats.

Identification of nuclear integrants of plastid DNAs (NUPTs). We used the blastN search to identify nuclear integrants of plastid DNAs (NUPTs) from chloroplast genome. The chloroplast genome of *S. oleracea* (NCBI accession ID:NC_002202.1) was used as the query and the YY and XX genome sequences were used as the subject sequences. The top blastN hits of chloroplast sequences were filtered if identity < 80%, aligned length < 200bp, and e-value > 1e-3 and totally 426 (722,873 bp) and 389 (823,776 bp) NUPTs insertions were chosen as the final NUPTs in YY and XX genomes, respectively (**Additional file 2: Fig. S7**)

miRNA annotation. Publicly available plant miRNAs were downloaded from miRBase (http://www.mirbase.org/; latest access on July 5, 2018) and mapped to both genomes using Bowtie [118] with at most 3 mismatches allowed. Alignments were filtered using a PERL script (filter_alignments.pl) implemented in miRDP1.3 package [119]. We further applied miRDeep-P program to identify and filter miRNAs, a total of 114 and 109 known miRNAs were predicted in YY and XX genomes, respectively (**Additional file 2: Fig. S8**).

Part2. Sex-chromosome analysis

Variants calling of F_1 population and natural re-sequenced population. Due to highly repetitive spinach YY genome, to obtain high-quality SNPs for defining the sex determination region (SDR), we applied repeat-masked YY genome for reads mapping. After trimming raw reads using Trimmomatic [80], clean reads were mapped to the repeat-masked YY genome using Bowtie2 [87]. The multi-mapped reads were filtered out and only unique mapped reads were retained. Then, the standard GATK pipeline with the HaplotypeCaller model [75] was applied for variants calling and generated raw VCF files.

In F_1 population, the genomes used for SDR definition (40 female and 39 male F_1 samples, **Additional file 3: Table S6**) were sequenced at depth ranging from 8.52× - 16.06× (with an average of 12.4× depth not including patents). According to the density distribution of mean read mapping depth (DP) per site of female and male samples (**Additional file 2: Fig. S4a**), only sites with missing data< 20%, minor allele frequency (MAF) > 0.05, mean mapping depth (DP) per site in between 3-35, depth by quality (DQ) > 2, genotype quality (GQ) > 10, and mapping quality (minQ) > 10 were kept. After filtering, 177,414 high-quality SNPs set were generated for downstream sex co-segratation markers analyses.

In natural re-sequenced population, the genomes used for SDR definition (26 female and 44 male accessions, **Additional file 3: Table S13**) were sequenced at depth ranging from

4.1× - 7.7× (with an average of 6.06× depth). According to the density distribution of mean read mapping depth (DP) per site of female and male accessions (**Additional file 2: Fig. S4b**), we applied only sites with missing data < 25%, minor allele frequency (MAF) > 0.05, mean mapping depth (DP) per site in between 2-20, minimum alleles = 2, maximum alleles = 2, and mapping quality (minQ) > 20 as cutoffs to remove low-quality SNPs. After filtering, 4,844,193 high-quality SNPs set were generated for downstream GWAS mapping, calculations of male-specific SNPs, *Fst* statistics, and Tajima's D value between female and male accessions.

Estimation of LTR-RTs insertion time. The time-course of LTR-RTs can be estimated by molecular clock based on the divergence of flanking LTRs of the same retrotransposon. Thus, we performed the precise annotation of LTR-RTs by LTR_retriever pipeline, https://github.com/oushujun/LTR_retriever/blob/master/LTR_retriever (GPL-3.0 License) to study the divergence of LTR-RTs. Each intact retrotransposon includes two long terminal repeats (LTRs), two target site duplications (TSDs), and coding regions encoded by protease, reverse transcriptase, integrase, and ribonuclease H domains was chosen for analysis. The insertion time (T) of those LTR retrotransposons was calculated using the formula $T=K/2r$, where K is the distance and r was set to 2.8e-9 as substitutions per site per year, as fossil evidence from Amaranthaceae family [77]. Moreover, we made an LTR-RTs insertion time comparison among MSY and its X counterpart, the sex chromosome, and whole-genome level from both male (YY) and female (XX) genomes. Also, the potential link between LTR-RTs burst from sex-linked region and genomic rearrangement was explored by comparing LTR-RTs insertion time from two genomic inversions, two insertions, and collinear regions across the SDR and X counterpart.

K-mers analysis of YY-specific contigs. K-mers of both YY and XX genomes were generated using k-mer counter DSK [120] with default parameters. YY contigs were decomposed into their constituent k-mers and queried each XX k-mers to get the proportion of k-mers of each YY contig shared with XX assembly. After that, the k-mer counts from the YY raw reads were used to estimate depth-of-coverage of each contig. The YY-specific contigs were then extracted using the parameters with a proportion of XX k-mers (P< 40%) and reads coverage of YY-contigs (10<coverage <200).

Part3. RNA-seq and transcriptome analysis for female and male flowers

Plant Materials. Spinach female II9A0012 (XX) and male II9A0075 (XY) accessions seeds (**Additional file 2: Fig. S9**) were grown in growth room with temperature set at 23°C, humidity 65%, and a 16 h photoperiod. Before the initiation of flowering, female plants were covered to avoid pollen contamination.

Flower developmental stages as mentioned in [79] were used to classify the stages for RNA-seq sampling tissues **(Additional file 2: Fig. S10a)**: **stage1** (flower bud size= 0.2-0.5 mm): two opposite sepal primordia established in the periphery of the meristem (male, female), **stage2** (flower bud size= 0.5-1mm): Two extra sepals form within the first whorl in space between initial sepal primordia; sepals expands laterally around the outer whorl and distally to surround the floral meristem (female), **stage3**: sepal primordia grow, four stamen primordia form in the periphery of the central dome; sepal covers the central dome and dome begin to differentiate into an ovary (female), **stage4**: stamen primordia develop to distinct anthers; central region of ovary form pistil and ovule differentiate in the ovary (female), **stage5**: anthers mature; ovule mature, stigma extends out of sepal closure (female). Those samples were collected and kept in liquid nitrogen.

Transcriptome assembly and identification of DEGs comparing male and female flowers at different developmental stages. A total of 19.7–28.9 million raw reads (female samples) and 17.9–30.5 million raw reads (male samples) per library at different developmental stages were obtained. After adapter sequences removal, clean reads were mapped to YY genome using STAR aligner. The uniquely mapped reads accounted for 91.41–93.44% of clean reads of male flowers and 92.13–93.06% of female flowers samples. To avoid potential variations in gene expression from the various genetic backgrounds, we normalized and calculated expression values of genes in FPKM when compared male with female flowers at each corresponding developmental stage. The female flowers were deemed as a reference when compared against the male ones. As the sex-biased genes exhibited higher expression in one than the other sex type, and always function downstream of sex-determining gene. Hence, the identification of sex-biased genes will be helpful to reveal the sex-differentiation/determination mechanism. Herein, a total of 2,014 differentially expressed genes (DEGs) between FS1 vs MS1, 3,025 genes between FS2 vs MS2, 5,059 genes between FS3 vs MS3, 6,066 genes between FS4 vs MS4, and 9,017 genes between FS5 vs MS5 were identified (**Additional file 2: Fig. S10)**.

GO and KEGG enrichments for different DEGs. GO enrichment analysis were performed in Blast2Go v4.119 [121]. KEGG enrichment analysis was performed in KOBAS v3.0 (http://kobas.cbi.pku.edu.cn/anno_iden.php), and the online tool Omicshare (www.omicshare.com/tools). We used the DEGs of each comparison as the tested gene sets and the entire gene model of YY assembly as the reference. The significance of enrichments was valued using Fisher's exact test. We performed the GO and KEGG enrichment for DEGs in the early and late-stage as well as DEGs linked to sex chromosomes between the male and female flowers. The results are as follows:

1) GO and KEGG enrichment of Early and Late-stage DEGs. KEGG analysis of 1246 DEGs in 'Early stage' showed the top enriched terms are pentose and glucuronate interconversions, tryptophan, and glutathione metabolism, cutin, subrerin, and wax biosynthesis, DNA replication, plant hormone signal transduction, alpha-Linolenic acid, and phenylpropanoid biosynthesis. GO enrichment showed the top enriched terms are the regulation of cell death, response to oxidative stress, cell wall organization or biogenesis, regulation of anatomical morphologies, and biological regulation DNA confirmation **(Additional file 2: Fig. S11a, c)**. However, the 2499 DEGs in 'Late stage' exhibited the enrichment of starch and sucrose metabolism, Fatty acid related pathways, Biosynthesis of secondary metabolites, and metabolic pathways genes through KEGG. Through functional GO enrichment analysis, we found the terms including single-organism transport, response to auxin, polysaccharide metabolic processes, developmental process involved in reproduction, carbohydrate catabolic pathways, and biological regulations were significantly enriched (P <0.05). As late stage (from anther differentiation to maturation) correspond to stamen and carpel development, most of the genes are enriched in metabolic pathways **(Additional file 2: Fig. S11b, d)**.

2) GO and KEGG enrichment of DEGs in sex chromosome. Moreover, KEGG analysis of differentially expressed genes linked to sex chromosome exhibited the enrichment of pathways including starch and sucrose metabolism, phenylpropanoid biosynthesis, ascorbate and aldarate metabolism, plant pathogen interaction, Plant hormone signal transduction, biosynthesis of secondary metabolites, and fatty acids-related pathways. Through GO enrichment analysis of these DEGs, developmental process involved in reproduction, cellular aromatic compounds metabolic process, regulation of DNA metabolic process, regulation of anatomical structure morphologies, reproductive process, and DNA recombination **(Additional file 2: Fig. S11e, f)**.

qPCR validation for key candidate genes in sex-determination model. To validate the expression patterns of key candidate genes used in sex-determination model, qRT-PCR analysis was performed on the same RNA samples used for library construction. The first-strand cDNA from 1μg of total RNA was synthesized by PrimeScript™ RT Reagent Kit and reaction was diluted to a final volume of 80 μl. TB Green™ Premix Ex Taq™ II kit (TaKaRa) was used to perform qRT-PCR with the 20 μl volume of final reaction; 1μl of cDNA, 1μM of each primer and10μl of TB Green™ PCR master mix. Amplification program was 95 °C for 3min; 40 cycles at 95 °C for 10 s and 50 °C for 30 s followed by disassociation stage as instructed by user's manual. The resulting qRT-PCR data was analyzed using the formula $2^{-\Delta\Delta Ct}$. *GAPDH* gene was adopted as control [122]. All the genes were repeated with three biological samples. The primers used in this analysis were provided in **Additional file 3: Table S17**. The Y-specific sex determinant candidate genes (*NRT1/ PTR* (YY20280); *EIF3* (YY20279)); Gibbrellin signaling genes (*DELLA* (YY25178); *GID1* (YY23913, YY23914, YY35921)); meristem termination and gynoecium related genes (*WUS* (YY00099), *CRC* (YY04840)) were selected for quantitative RT-PCR analysis at different flower stages for both sex types to verify the RNA-seq data **(Additional file 2: Fig. S16)**. All the selected genes showed consistent expression with RNA-seq.

Part4. Resequencing and Population genomics

De novo assembly of chloroplast genome. The filtered Illumina reads of 108 resequencing accessions from each of the three species, *S. oleracea*, *S. turkestanica*, and *S.tetrandra* were assembled separately using NOVOPlasty v.4.1 [123] with the following parameters: Genome Range = 120000-180000, Type = chloro, Kmer=39, using published *S. oleracea* complete chloroplast sequence (GenBank accession NO. *NC002202*) as the seed. The assembled chloroplast genomes were viewed and edited using Bandage [124], and then annotated using online annotation tools GeSeq (https://chlorobox.mpimp-golm.mpg.de/geseq.html/). OGDRAW (http://ogdraw.mpimp-golm.mpg.de/) [125] was used to visualize the physical map and annotations.

SplitsTree analyses of reticulate evolution. To test if board hybridization events occurred during spinach evolution and domestication. We reconstructed a phylogenetic network using Splits Tree (http://splitstree.org/) to identify the signals of reticulate evolution among 112 *Spinacia* accessions of three species (*S. oleracea*, *S. turkestanica*, and *S. tetrandra*) **(Additional file 2: Fig. S24)**. The result shows a wide range of reticulate evolution patterns, therefore indicating frequent introgression events among cultivars and two wild species companying with their evolution and domestication history.

Plant material, RNA-seq analysis. Two wild type accessions (*S. tetranda*: PI677114 and *S. turkestanica*: PI677111) with extreme phenotypes (lower plant height and less leaf area when compared to cultivars) were chosen after screening of 20 wild type accessions. The 1st internode (from bottom to up) and the early-stage leaf samples (from middle to top section of plant) were collected when plant attain the full height. Similar sampling was done for *S. oleracea* accessions (Sp75 and Cornell-NO. 9). Two replicates for leaf, and one replicate for internode samples of each wild type accessions (*S. tetranda*: PI677114 and *S. turkestanica*: PI677111) were collected. For cultivated accessions, a replicate of two cultivars (Sp75 and Cornell-NO. 9) for leaf and internode samples were collected for RNA extraction. RNA-sequencing and expression data analysis was performed with the same method mentioned in **Main text, Methods Part 3 (Analyses of the transcriptomes of female and male flowers at five stages using RNA-seq).**

Morphological and cytological measurements of leaf and internode traits. The leaf and internode were measured using both morphological and cytological methods. The morphological measurement of leaf area and internode length was performed using caliper directly for both cultivated (Sp75) and wild-type spinach (*S. tetranda*: PI677114 and *S. turkestanica*: PI677111). To observe the cell size of leaf and internode, epidermal layer of leaf and the 1st internode (from bottom to up) of both cultivars (Sp75) and wild species (*S. tetranda*: PI677114 and *S. turkestanica*: PI677111) were treated with Propidium Iodide Ready FlowTM Reagent and kept in dark for 20 minutes to ensure staining. Samples were mounted on a cover glass slide and imaged using a Leica TCS SP8 microscope (Leica, Wetzlar, Germany). As parameters measured (like internode length, internode cell length, internode cell area, leaf area, leaf cell length and leaf cell area) were similar for both wild type accessions, average values of *S. tetranda*: PI677114 and *S. turkestanica*: PI677111 were used when compared with values of cultivated Sp75 (**Main text; Fig. 5f: f16-21**). Statistical significance of each comparison was measured using *t*-test.

The comparison of internode length and leaf size between cultivated *S. oleracea* (Sp75) **(Main text; Fig. 5f: f1-f5)** and wild (average value of *S. turkestanica* and S. *tetranda)* **(Main text; Fig. 5f: f6-f15)** showed significant difference (both *P*-value< 0.05) for internode length (f16) and leaf area (f19). In addition, both the cell length and cell area of internode epidermis showed substantial differences (**Main text; Fig. 5f: f17, 18**, both *P*-value < 0.05), but the cell length and cell area of leaf epidermis exhibited minor differences (**Main text; Fig. 5f: f20, 21**, both *P*-value > 0.05) between cultivars (**f3, f5**) and wild species (**f8, f10, f13, f15**).

qPCR validation for key candidate genes in selective sweeps and introgression region. To validate the expression patterns of key candidate genes found in selective sweeps analysis, qRT-PCR analysis was performed on the RNA samples of cultivars (Sp75 and Cornell-NO. 9) and wild species (*S. tetranda*: PI677114 and *S. turkestanica*: PI677111). The protocol of qRT-PCR is adopted from validations for key genes involved in sex differentiationor determination model. The primers used in this analysis are provided in **Additional file 3: Table S17**. The candidate genes *COBRA4*(YY15166), *CDC123* (YY15211), *TCP17*(YY15171) might controlling internode elongation and *PYM* (YY28473), *TCP17*(YY15171) might controlling leaf area enlargement were selected for qRT-PCR analysis for both cultivars and wild species to verify the RNA-seq data. The qRT-PCR results confirmed *COBRA4/ CDC123* with high expression in cultivar internode and *TCP17* had higher expression in wild internode; *PYM* genes with high expression in cultivar leaves and *TCP17* had higher expression in wild leaves **(Additional file 2: Fig. S23)**.
