# Supplementary material for: The spinach YY genome reveals sex chromosome evolution, domestication, and introgression history of the species
Source: Genome Biol. 2022 Mar 7;23:75. doi: 10.1186/s13059-022-02633-x (PMC8902716; doi:10.1186/s13059-022-02633-x)
Supplement: Supplementary file 2 — Additional file 2. Supplementary figures. Figure S1-S28. [file 13059_2022_2633_MOESM2_ESM.pdf]

Supplementary figures

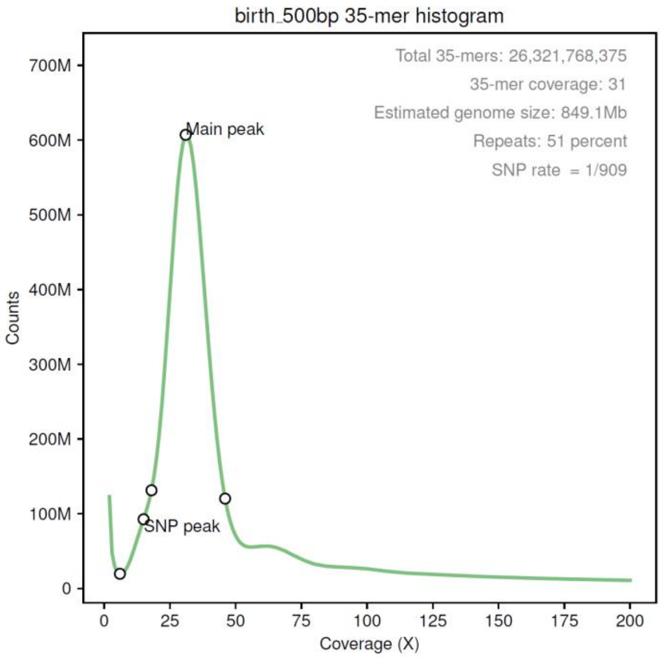

Figure S1. The 35-mer depth distribution of the Illumina reads of YY genome. A total of 26,321,768,375 30-mers with coverage of 31 × were obtained. Spinach genome size was estimated to be 849.1 Mb based on the formula: Total number of *K-mers* /Position of peak depth = 26,321,768,375 / 31 = 849089302 bp

a

b

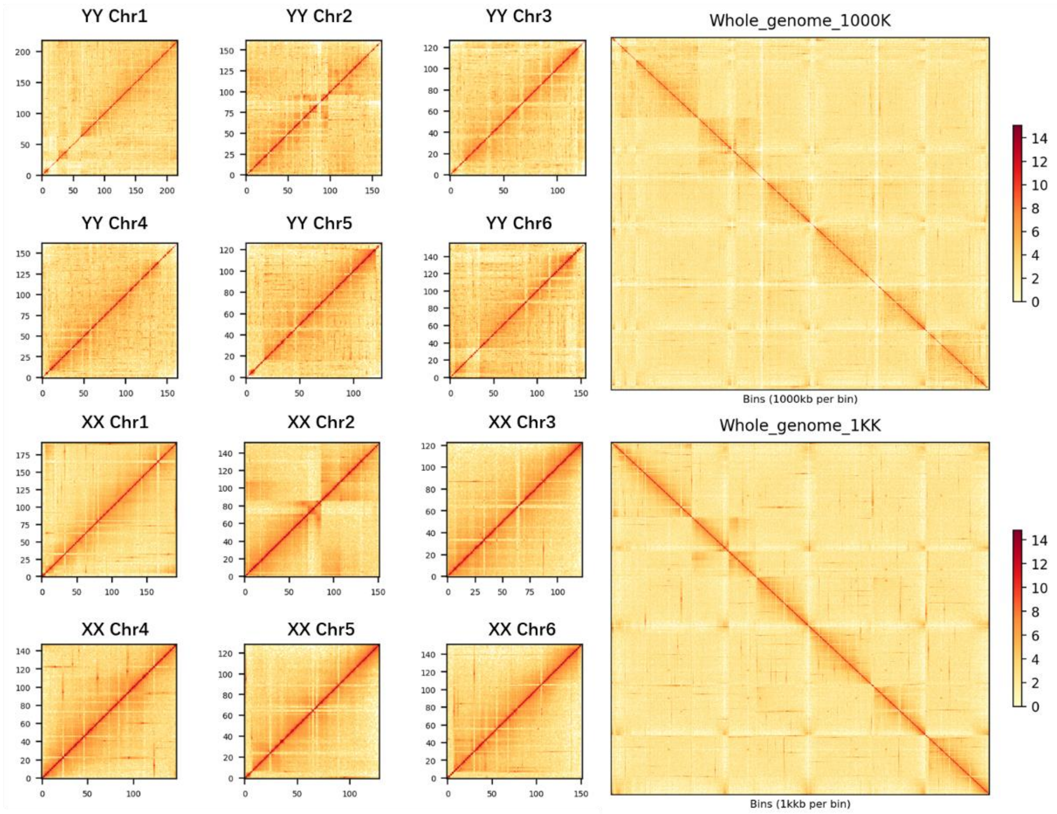

Figure S2. Genome-wide analysis of chromatin interactions at 1-Mb resolution in the YY (a) and XX (b) genome. The chromosomal assemblies were evaluated based on the heatmap analysis of chromatin interactions, which revealed a well-organized contact pattern of interactions along the diagonals within each chromosome of both YY and XX genomes

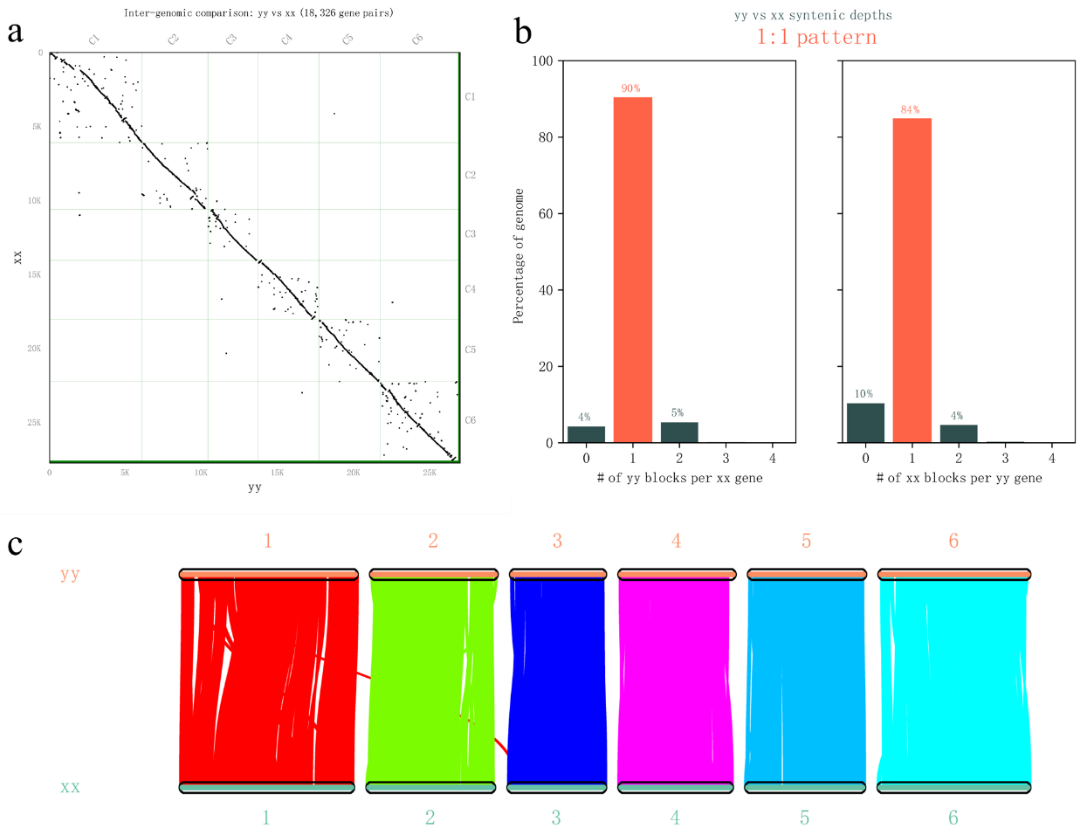

Figure S3. Syntenic analysis of YY and XX genome. a, Dot plot of YY and XX gene models with 18326 unique gene pairs. **b,** Syntenic depths between YY and XX genomes. **c,** Macro-syntenies between six homologous chromosomes of YY and XX genomes.

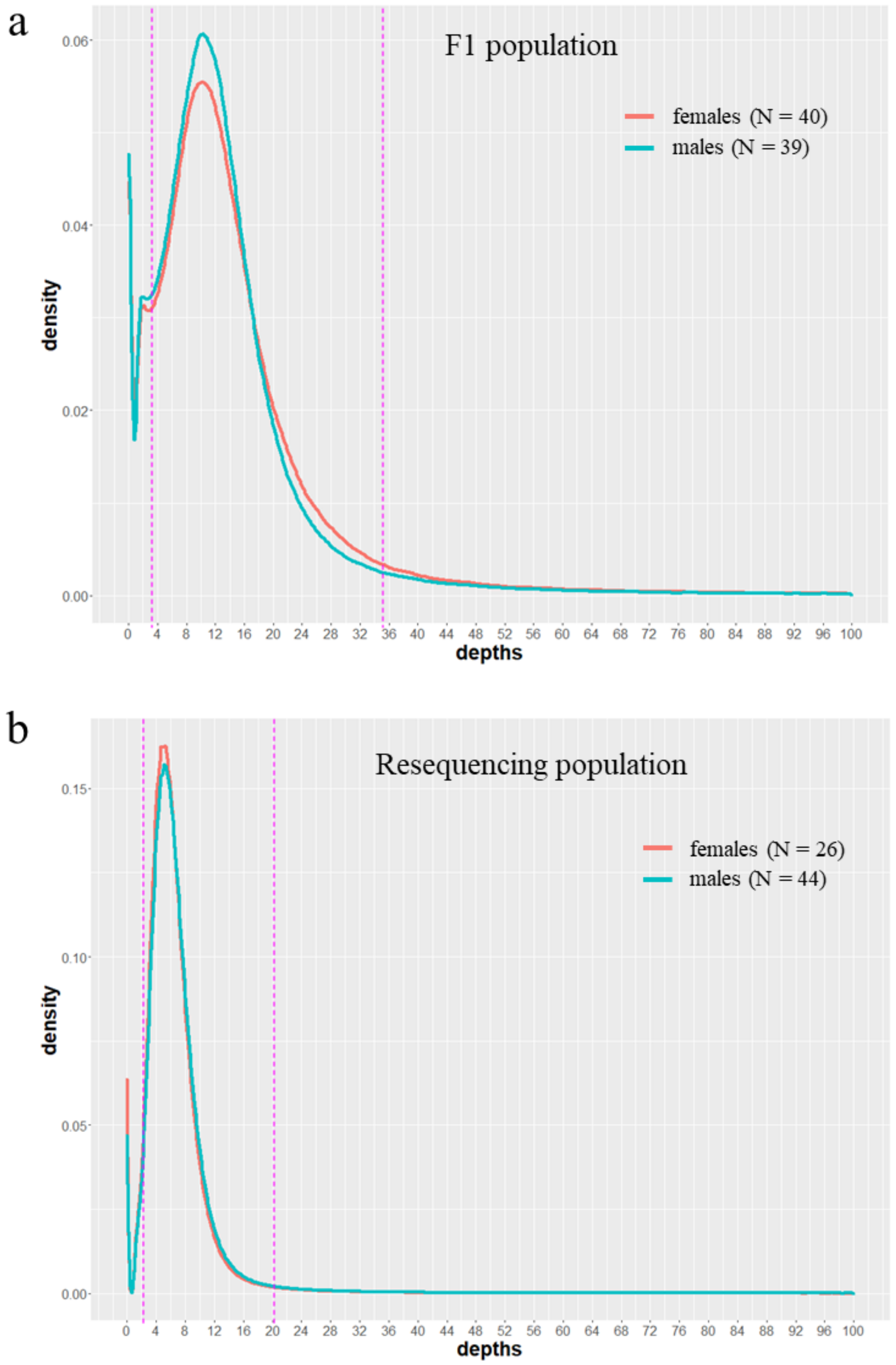

Figure S4. Density distribution of mean read mapping depth (DP) per site of female and male accessions onto repeat-masked YY genome. a, F1 population. The dashed line showing the cutoffs of DP value between 3<=DP<=35 for SNP filtering used in sex co-segregation markers analysis. **b**, Resequencing population. The dashed line showing the cutoffs of DP value between 2<=DP<=20 for SNP filtering used for GWAS mapping, calculation of male-specific SNPs, *Fst* statistics, and Tajima's D value between females and males.

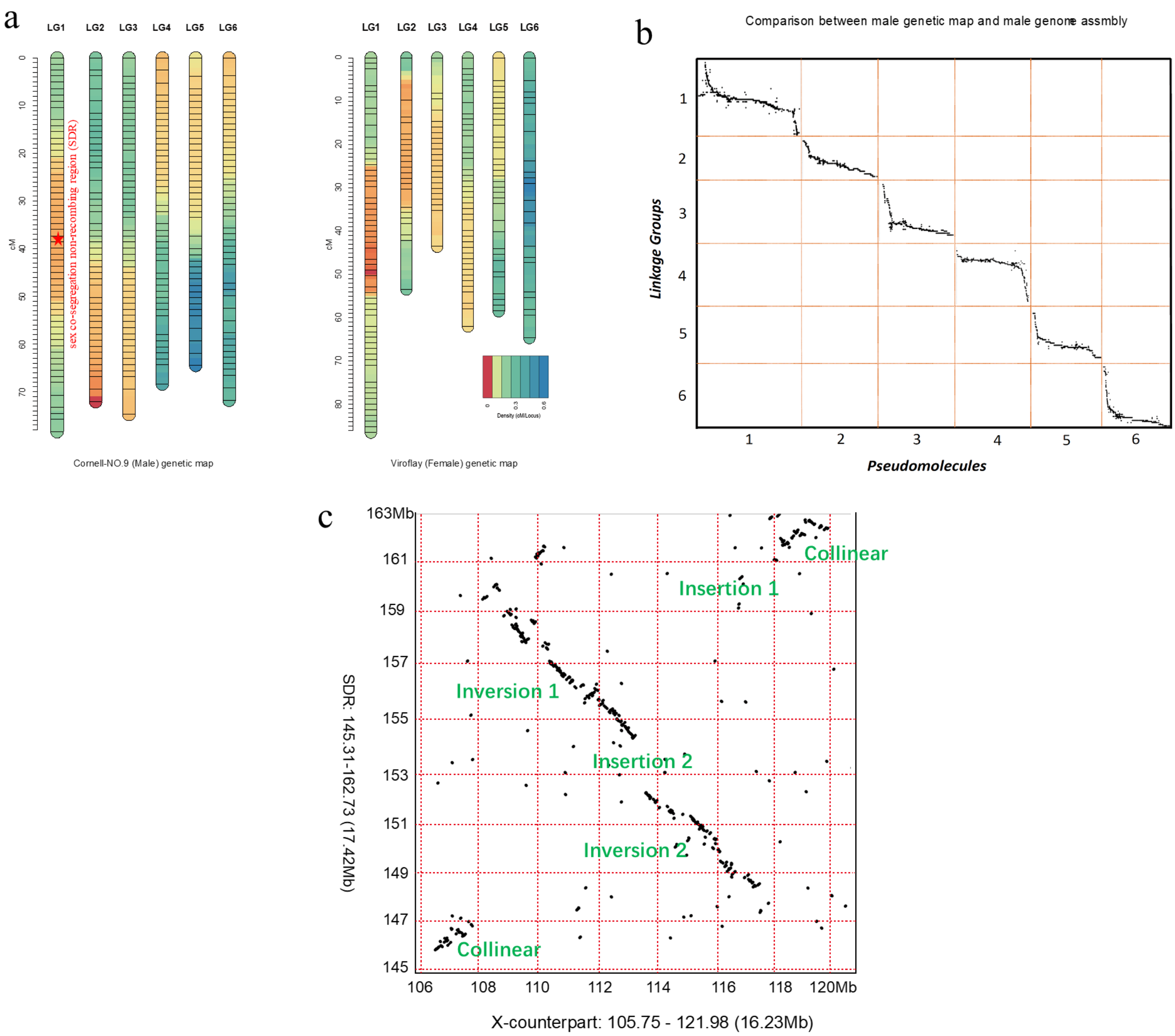

Figure S5. Genetic Maps and Identification of sex-determination region (SDR). a, Bin map of Cornell-NO.9 genetic map and Viroflay (female) genetic map, with reduced bin markers density surrounding SDR and its X counterpart. **b,** Matrix plot of "Cornell-NO.9" genetic map and YY genome assembly. **c,** Pairwise comparison of sequences between SDR (YY-Chr1: 145.31-162.73 Mb, Size=17.42 Mb) and X counterpart (XX-Chr1: 105.75-121.98 Mb, Size=16.23 Mb) detected by mummer, with two inversions: Inversion1 and Inversion2; and two insertions: Insertion1 and Insertion2.

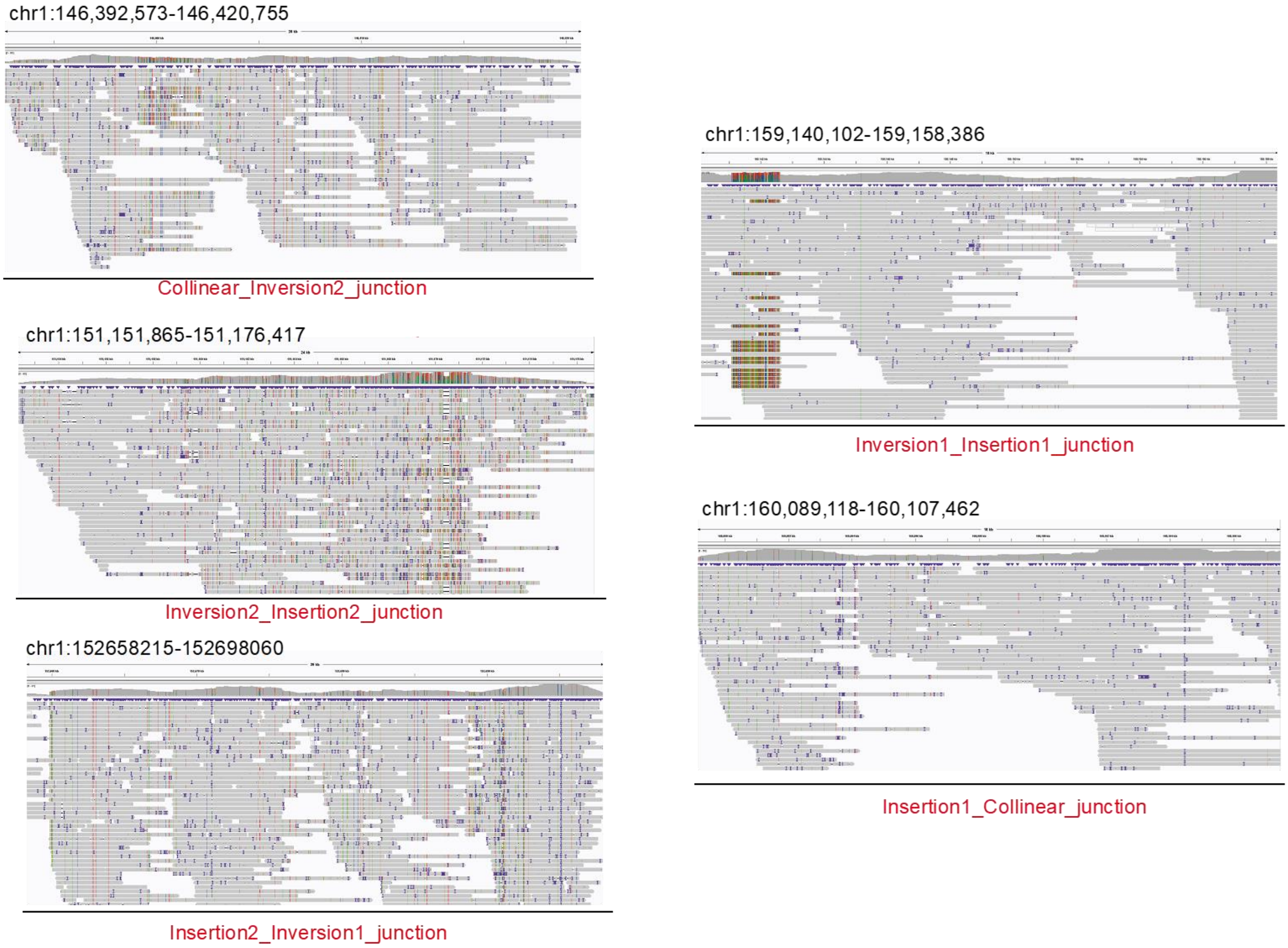

Figure S6. Pacbio reads mapping graphs onto YY genome for junction regions of structure variations within SDR. The junctions are adjoined regions between structure variations, including Inversion 1, Inversion 2, Insertion 1, Insertion 2, and collinear regions of SDR.

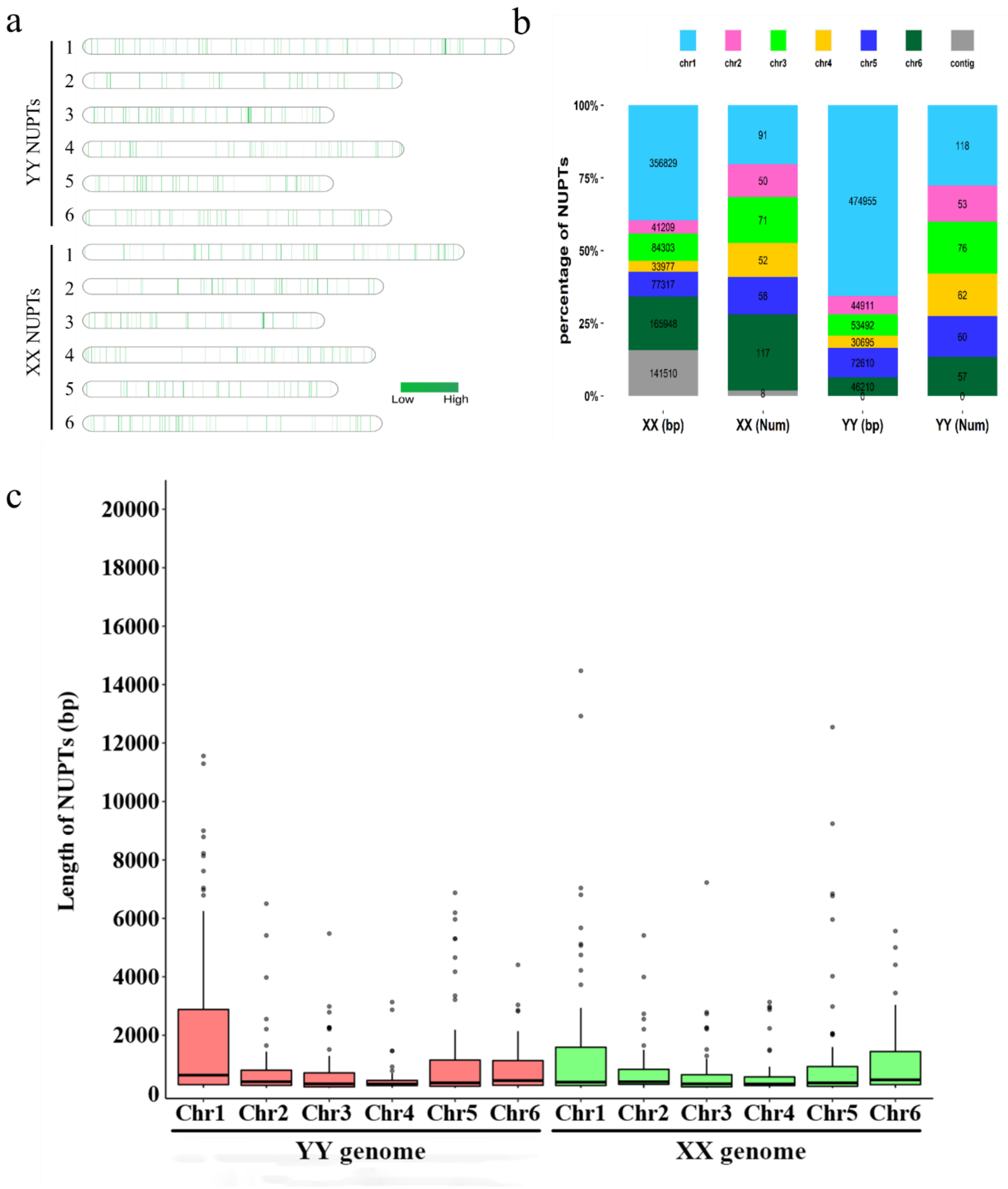

Figure S7. Comparison of NUPTs insertions from YY and XX genomes. a, The distribution of nuclear integrants of plastid DNAs (NUPTs) in YY and XX genomes. **b,** Percentages of NUPTs in YY and XX genomes. Comparisons revealed Y chromosome accumulated the highest density of NUPTs in terms of number (num) and length (bp). **c,** The largest NUPTs insertions were observed from the X chromosome (129-kb) and the Y chromosome (112-kb) from two genomes respectively.

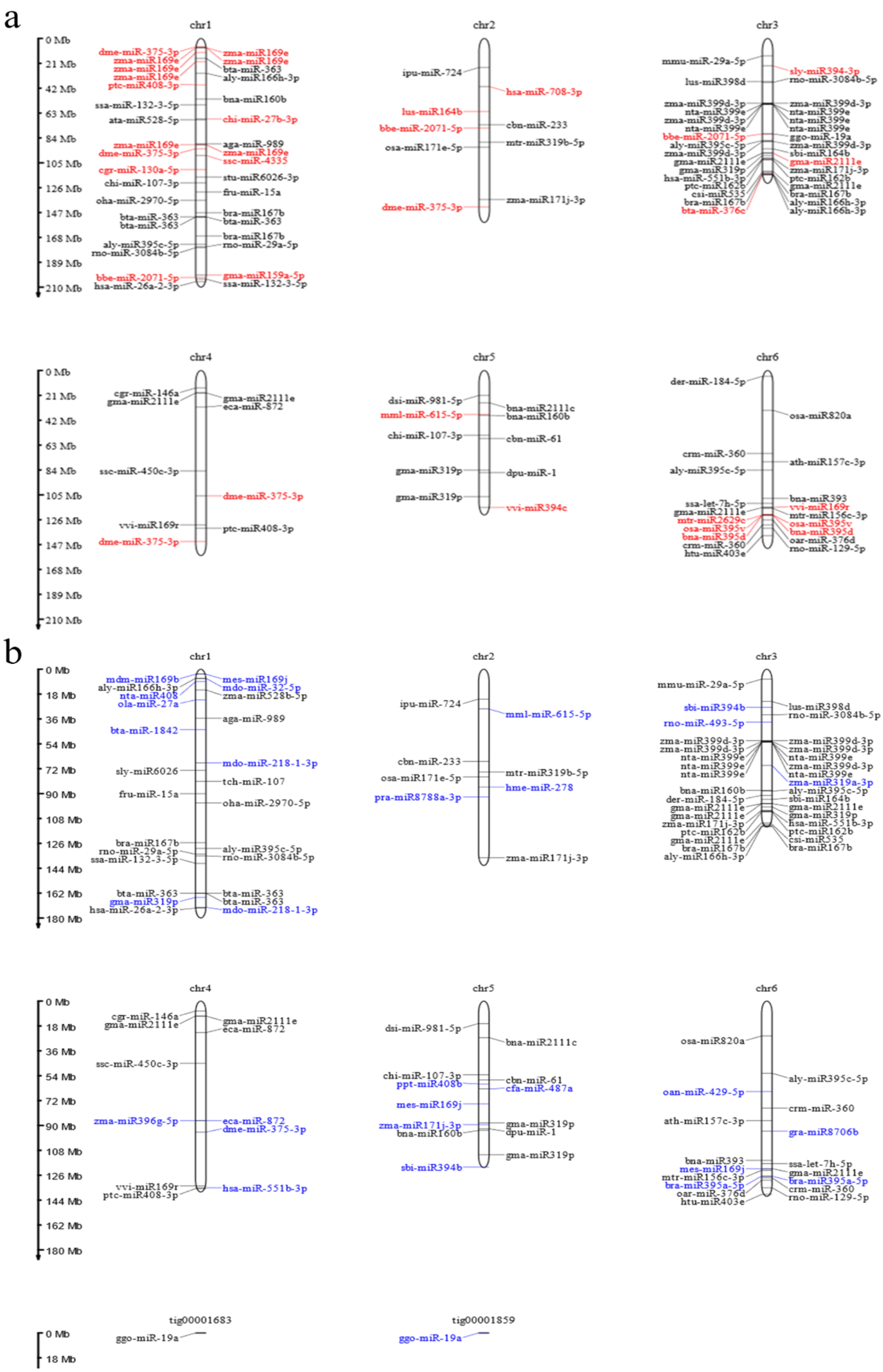

Figure S8. Predicted miRNA in YY(a) and XX(b) genome. The red color highlights the YY-specific miRNAs, while blue color highlights the XX-specific miRNAs.

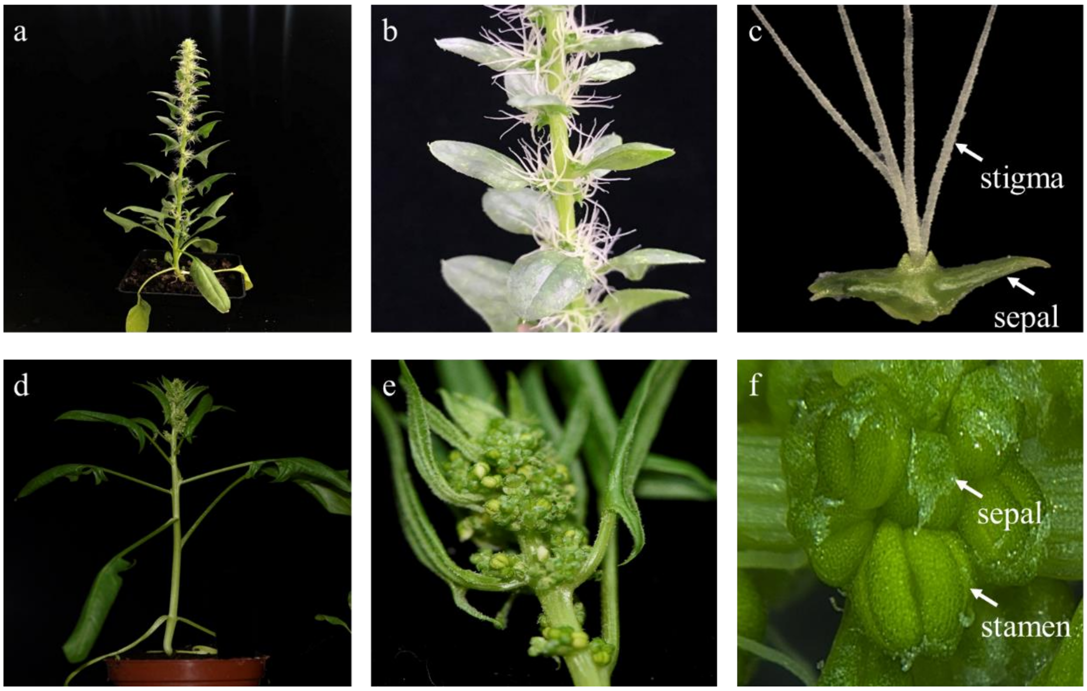

Figure S9. Characteristics of female (XX) and male (XY) flowers used in RNA sequencing. **a**, **b** and **c** indicate female flower of II9A0012 accession, while **d**, **e** and **f** indicate male flower of II9A0075 accession. White arrow marked stigma, stamen, and sepal.

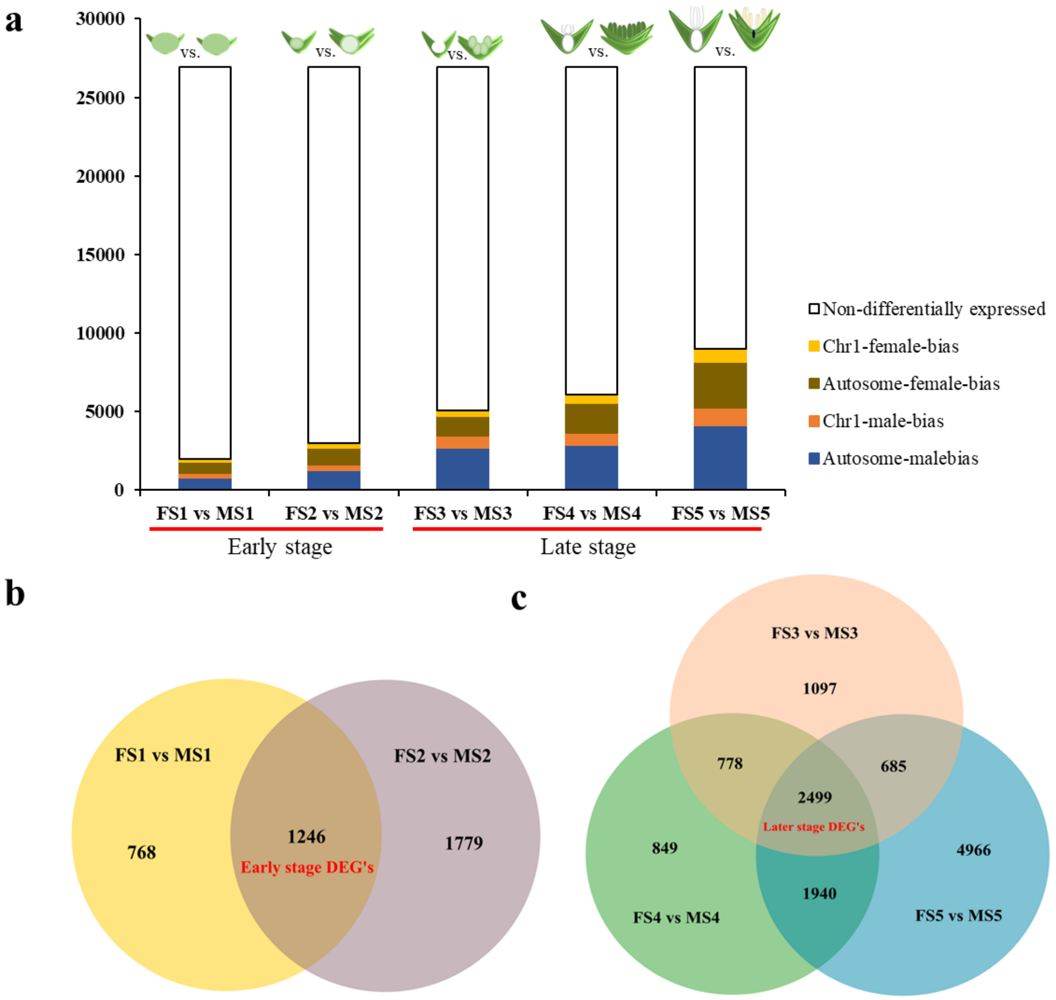

Figure S10. a, Summary of sexual-biased genes from female and male flower at different developmental stages. **b,** DEGs of Early stage. **c,** DEGs of Later stage

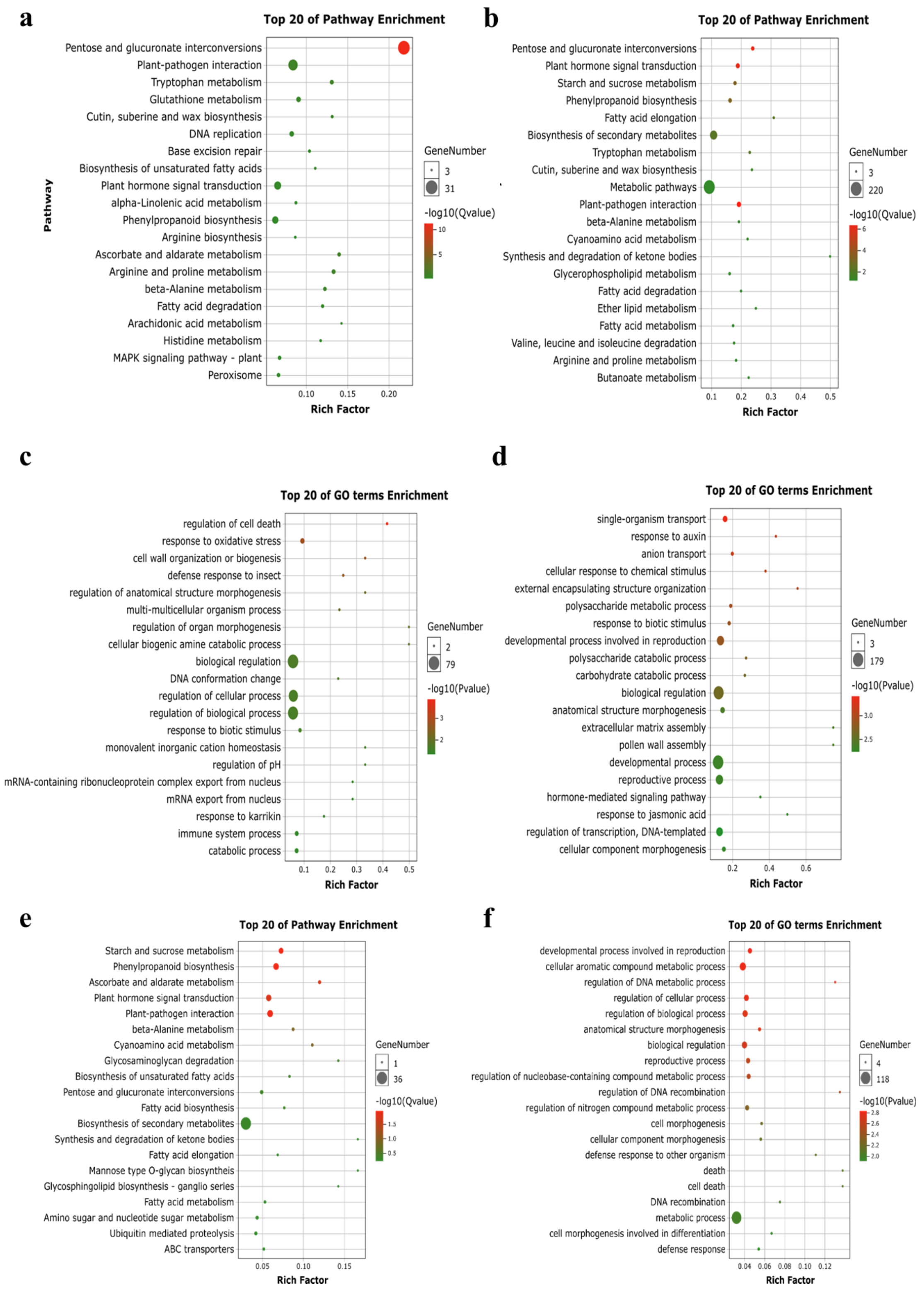

Figure S11. Top KEGG and GO enrichment pathway terms for DEG's at early (a & c) and late (b & d) stages, as well as DEGs on sex-chromosome (e & f).

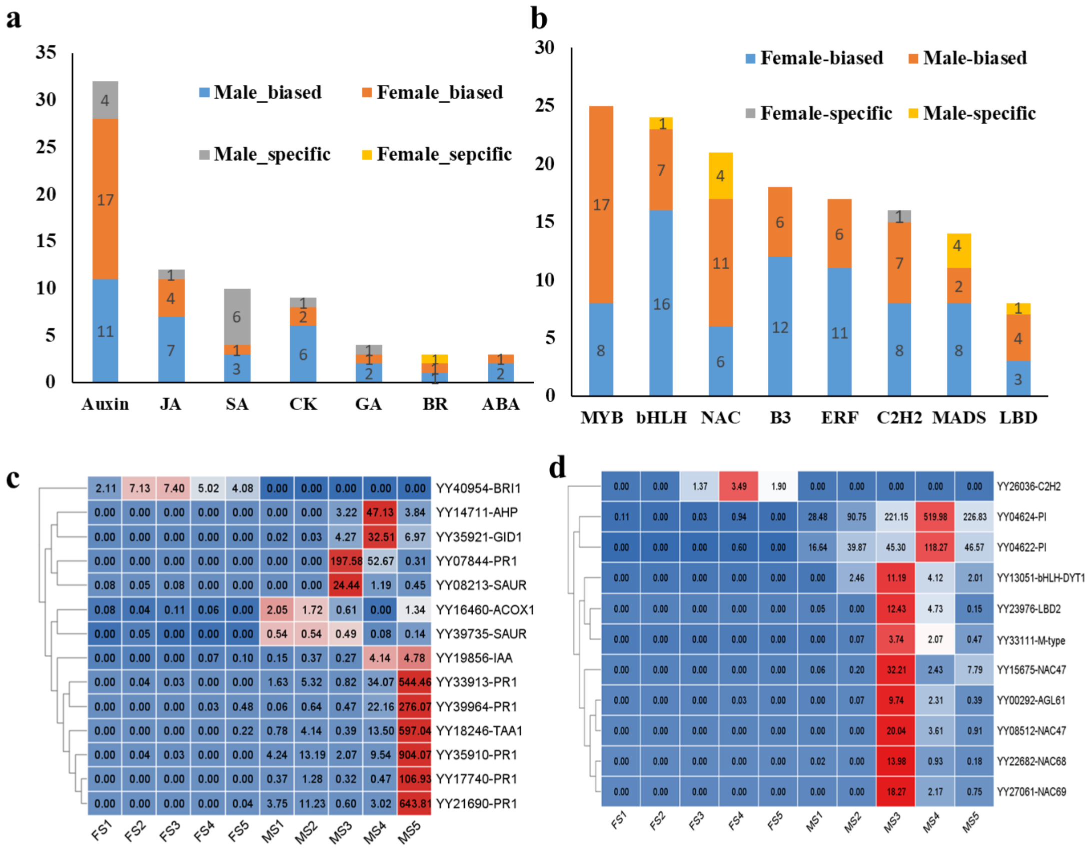

Figure S12. a. Enriched hormone-related genes. **b.** Enriched TF-families at different stages. **c.** Sex-specific expressed hormone-related genes. **d.** Sex-specific expressed TF genes.

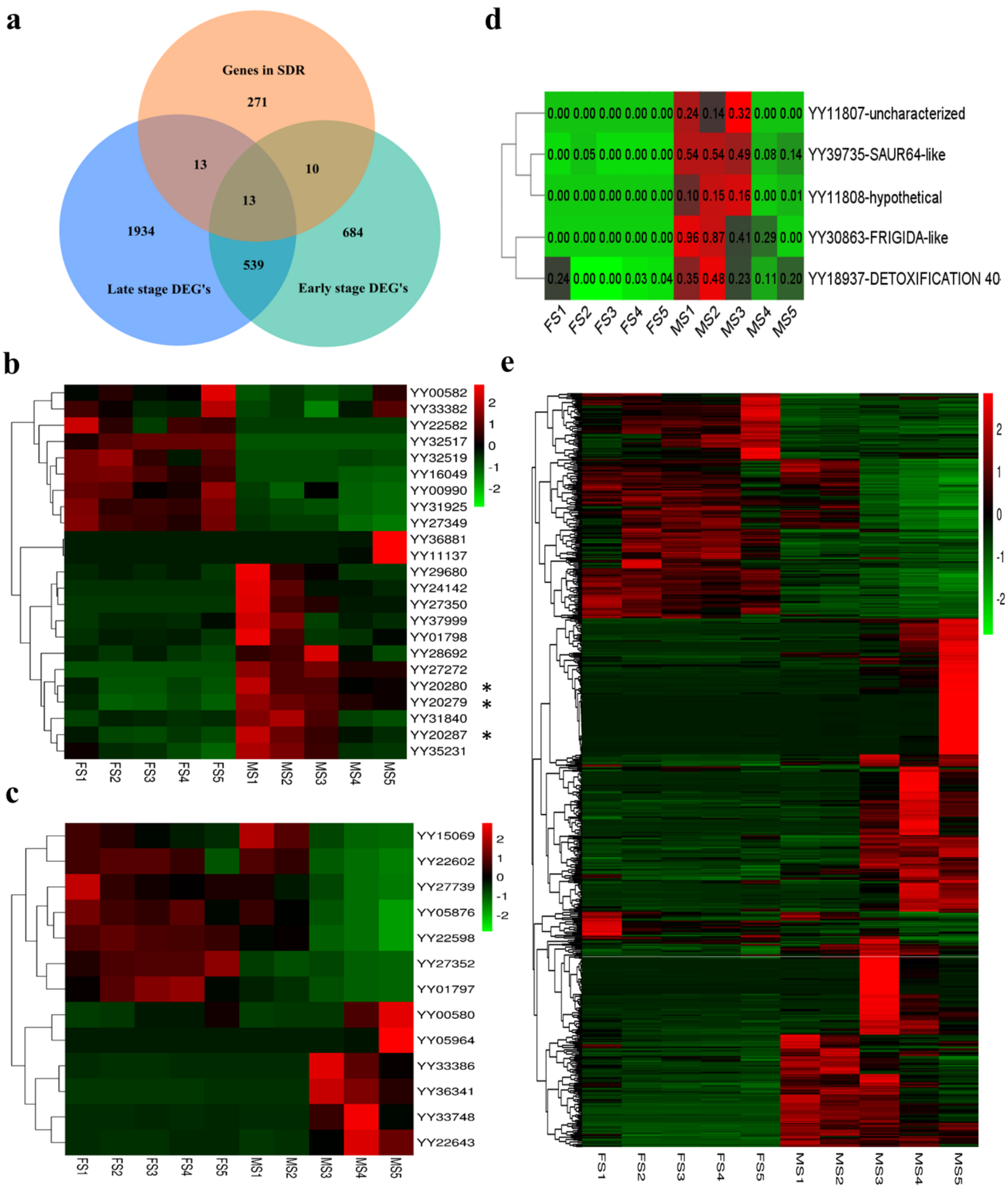

Figure S13. a, Venn diagram of differentially expressed genes (DEGs) in SDR at early, late, and throughout flower development. **b**, Expression pattern of potential 23 DEGs in SDR at early or all stages flower development, * represents Y-specific genes **c,** The 13 DEGs in SDR at later stage. **d,** Y-specific genes on pseudo-autosome region (PAR) of the Y chromosome with male-specific expression. **e,** Expression pattern of DEGs in PAR of the Y chromosome.

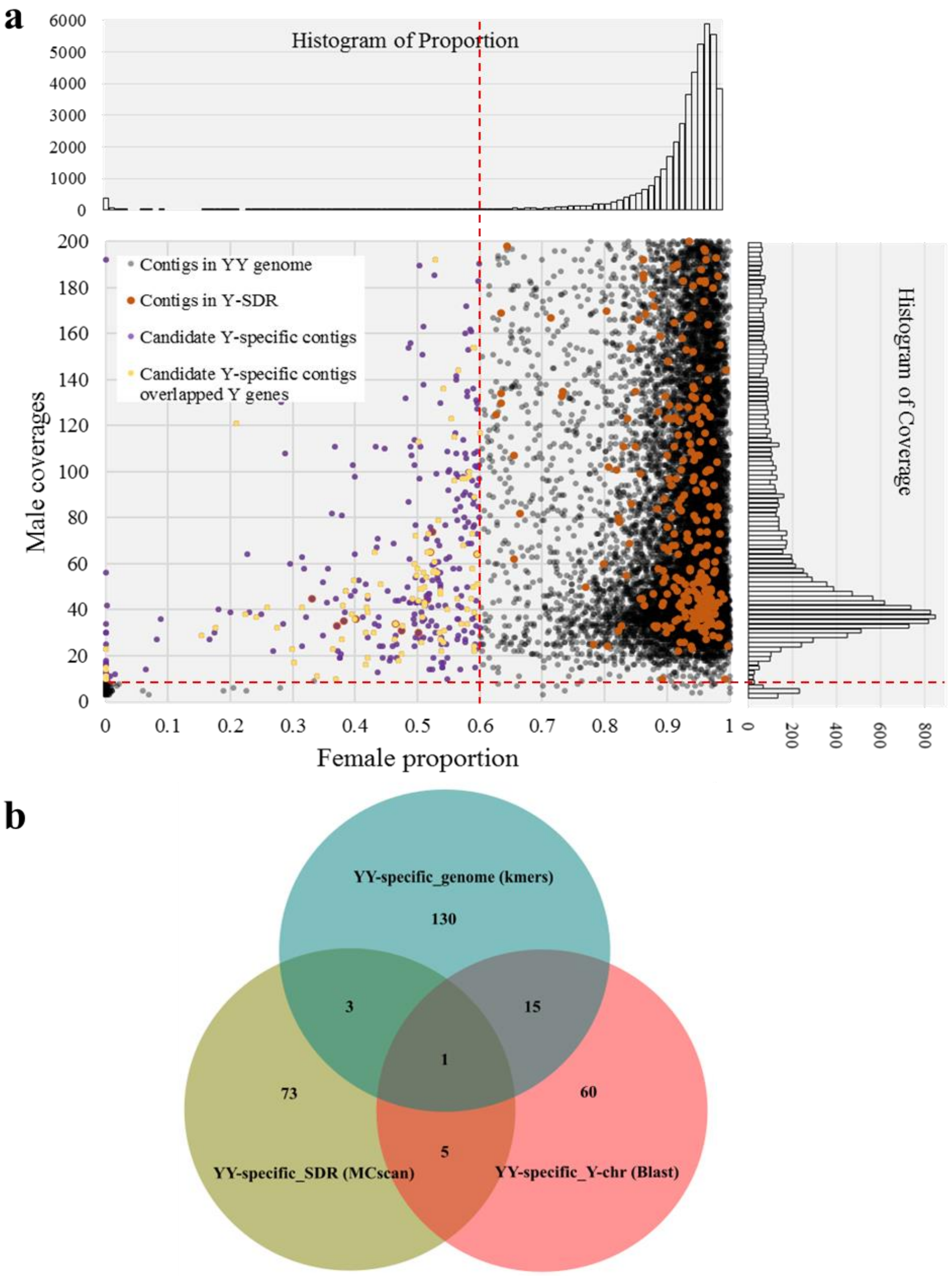

Figure S14. Identification of YY-specific candidate genes. a, Identification of Y-specific contigs by *k-mers* method via retrieving the male coverage for each Illumina read from male genome against its overlapped portions with female reference. Each point stands for a single contig, with different colors corresponding to certain regions. Distribution of the female proportion and male depth of coverage values for reads from male genome was represented by stacked histograms, respectively. The cut-off is shown as a dashed red line, with contigs to the left above (with female proportion < 40%, 10 < Male coverage < 200) of the line classified as candidate Y-specific contigs (purple dots). We also highlighted the contigs anchored to SDR of the Y chromosome (brown dots) and candidate Y-specific contigs overlapped Y genes (yellow dots). **b,** Overlapped YY-specific candidate genes identified using three methods: i) 82 YY-specific genes were identified in SDR using MCscan as previously revealed in sex-chromosome part; ii) 81 genes in Y-chromosome using Blast searches and iii) 149 genes in the whole YY genome identified using *k-mers* method. YY20280 (*NRT1/PTR 6.4*) was detected in all three methods.

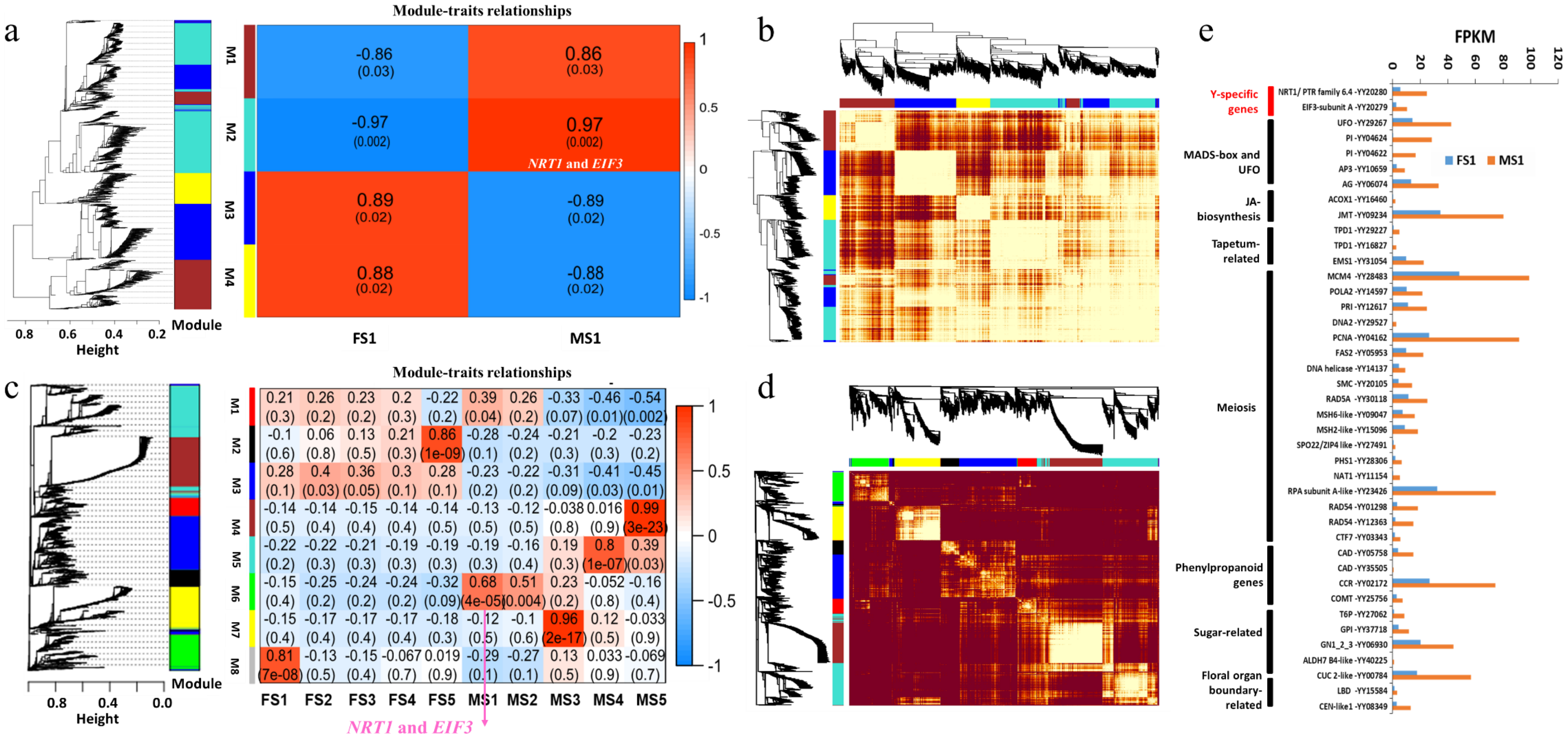

Figure S15. Co-expression network for DEGs between female (F) and male (M) flowers from first stages (S1) and all five stages (S1-S5). a. DEGs in Stage1 (S1) were clustered in 4 modules, M2 (male-module) is highly correlated to male stage 1 (MS1), Y-specific genes (*NRT1/PTR* and *EIF3*) are nested in this module. Heatmap representing associations between modules and traits revealed a distinct negative correlation between male and female modules, suggesting the modules are functionally connected. **b.** Heatmap of the gene co-expression network in Stage 1 (S1) DEGs. The heatmap descripted adjacencies among genes in the analysis. A single gene corresponds to each row and column of the heatmap. **c.** DEGs in Stage1-5 (S1-S5) were clustered in 8 modules. M6 (male-module) is highly correlated to male stage 1 (MS1). *NRT1/PTR* and *EIF3* are nested in this module. **d**. Heatmap of the gene co-expression network in Stage 1-5 (S1-S5) DEGs. **e.** Transcript expression of first-degree genes co-expressed with Y-specific*NRT1* (YY20280) and *EIF3* (YY20279) genes in Figure 3c.

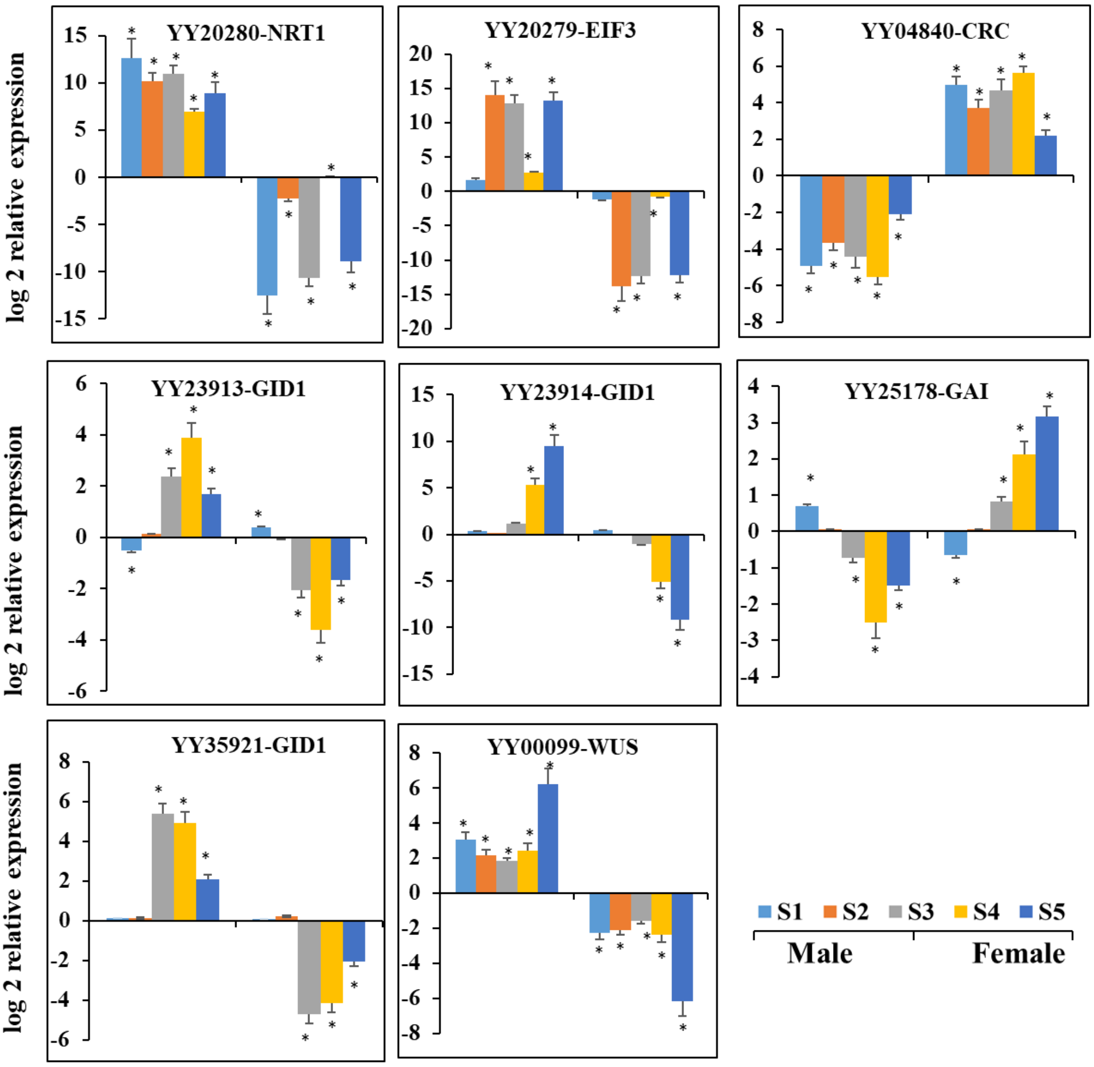

Figure S16. Log2-transformed relative expression of differentially expressed genes at different developmental stages between male and female flowers. Error bars depict standard deviation of the mean. *Denotes delta Ct values that were significantly different between spinach types ($P < 0.05$).

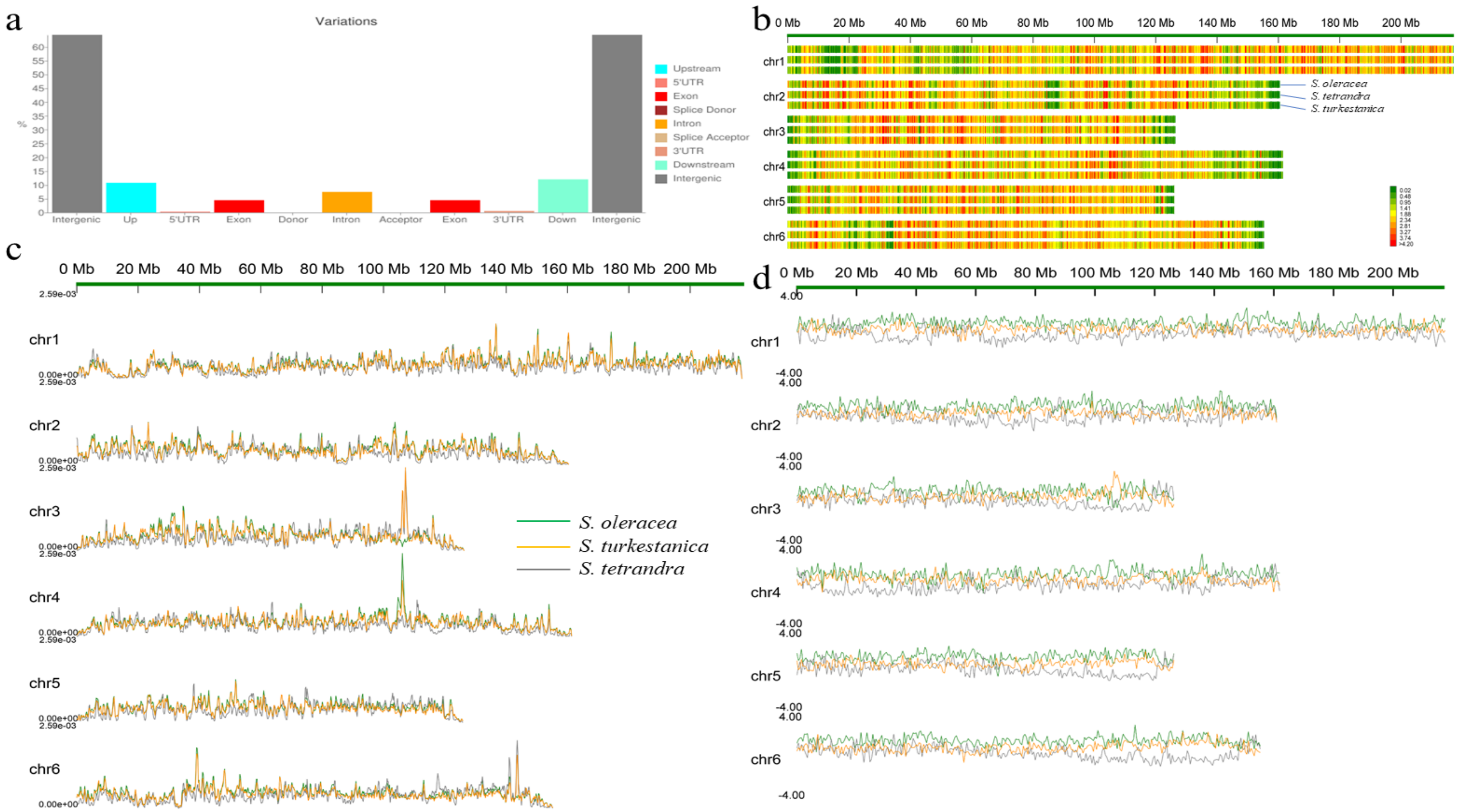

Figure S17. **Summary of genomic diversity of *Spinacia* accessions. a,** The fraction of effects by type and region for all the variations of 112 accessions. Heatmap showing SNP density (**b**), and Line plot showing Nucleotide diversity (**c**), and Tajima's D (**d**) for cultivated species *Spinacia oleracea*, as well as wild relatives *S. turkestanica* and *S. tetrandra*. The nucleotide diversity (π) of *S. oleracea* ($0.48\pm0.32\times10^{-3}$) was close to that of wild *S. turkestanica* ($0.43\pm0.29\times10^{-3}$) but much higher than that of *S. tetrandra* ($0.12\pm0.10\times10^{-3}$). However, the Tajima's D value (1.52±0.77) of *S. oleracea* was the highest comparing with *S. turkestanica* (0.93±0.64) and *S. tetrandra* (0.58±0.91).

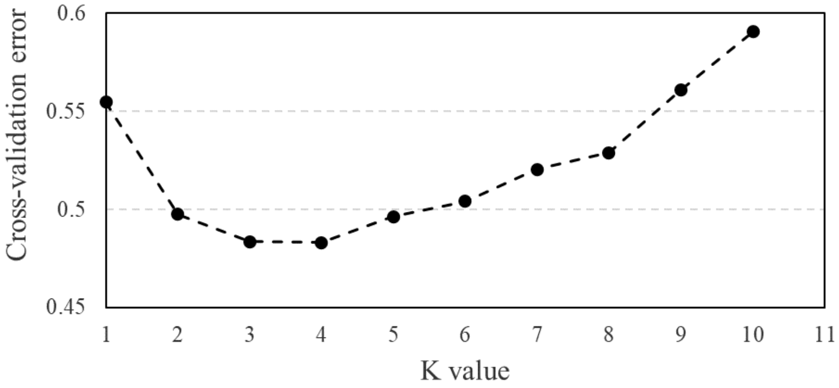

Figure S18. **The cross-validation error for admixture analysis, showing the optimal clustering is K=4.**

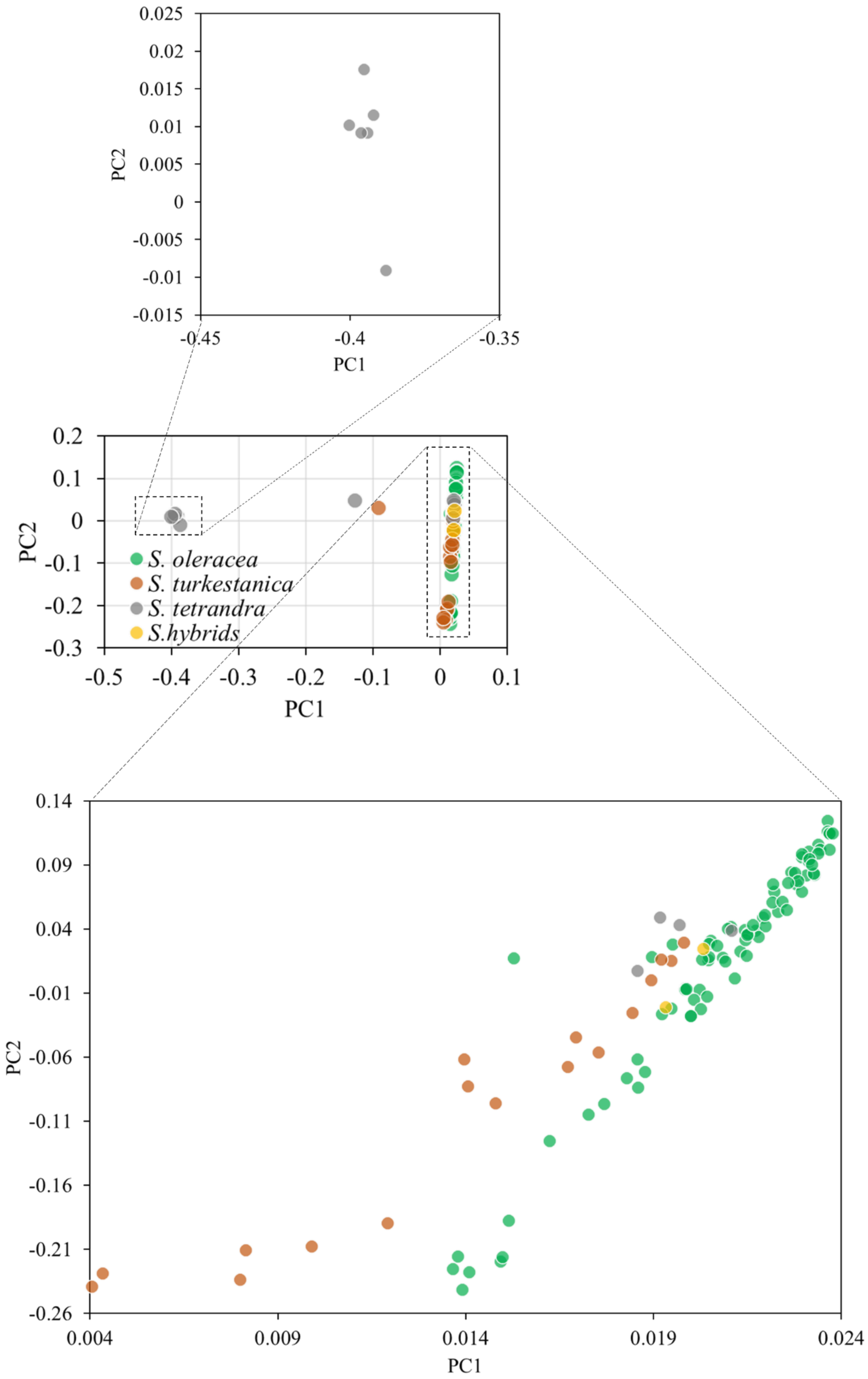

Figure S19. PCA analysis shows separation of *S. oleracea* population from populations of wild species *S. turkestanica* and *S. tetrandra*.

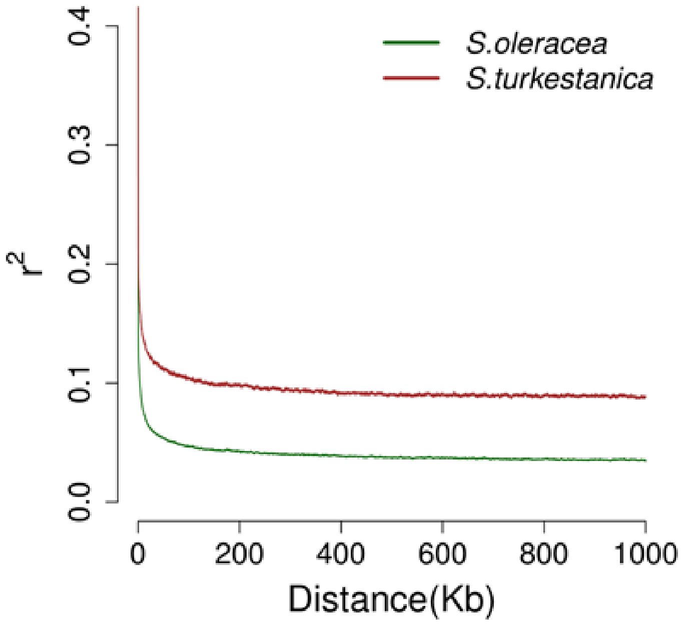

Figure S20. **LD decay of cultivated *S. oleracea* and wild *S. turkestanica*, with *S. oleracea* reaching the baseline at ~ 5.7 kb, while *S. turkestanica* at ~ 3.3 kb.**

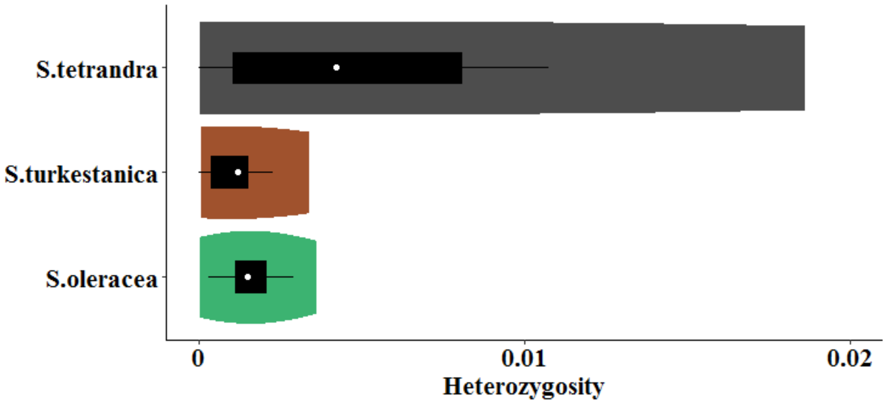

Figure S21. Heterozygosity rates of three *Spinacia* species. The averaged median (0.16±0.06%) of heterozygosity rate for *S. oleracea* was higher than that of *S. turkestanica* (0.10%±0.07%), but lower than *S. tetrandra* (0.47±0.43%).

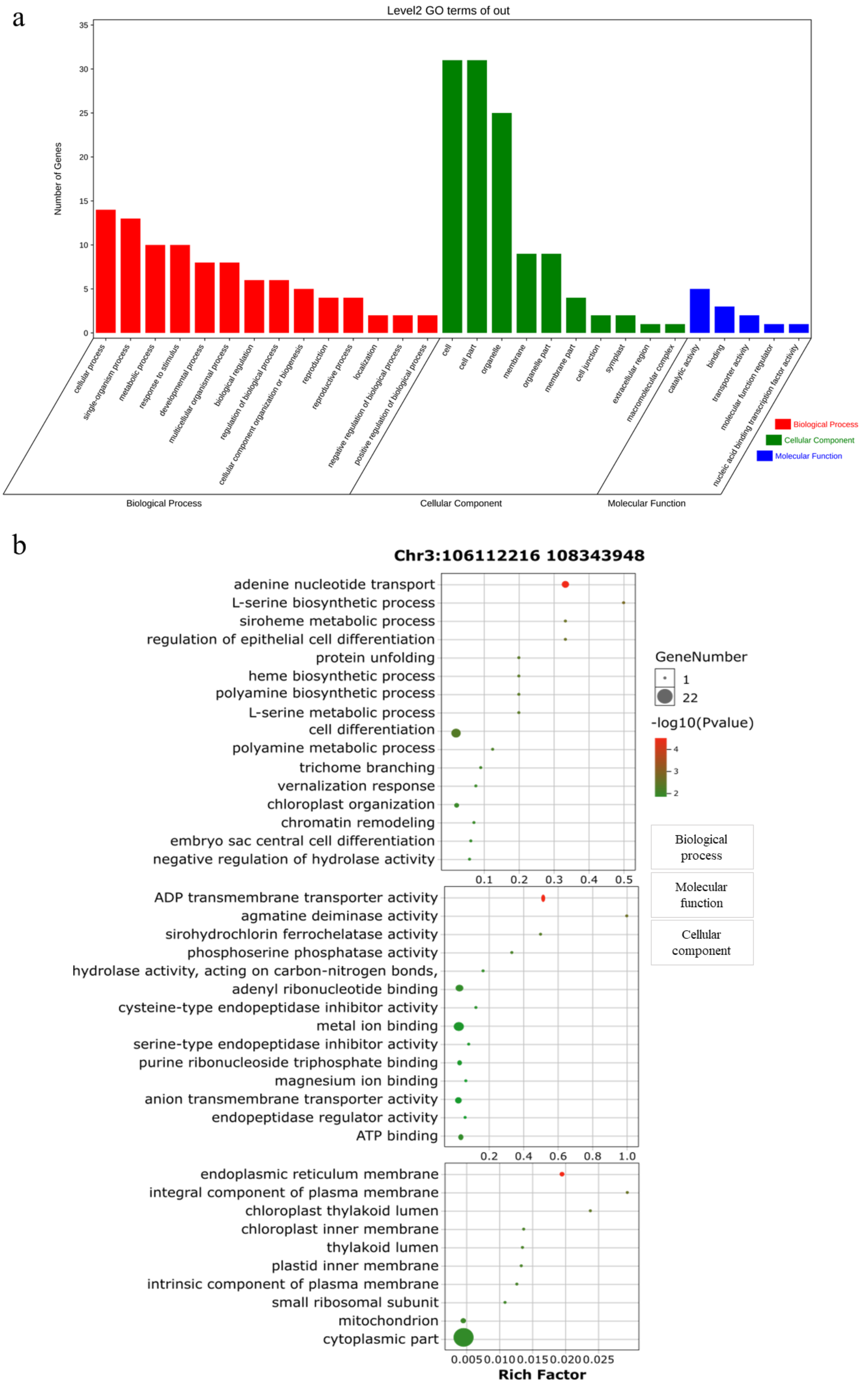

Figure S22. GO enrichment of genes in selective swept region (a) and in region Chr3:106112216-108343948 (b) with strong selection signal in YY genome

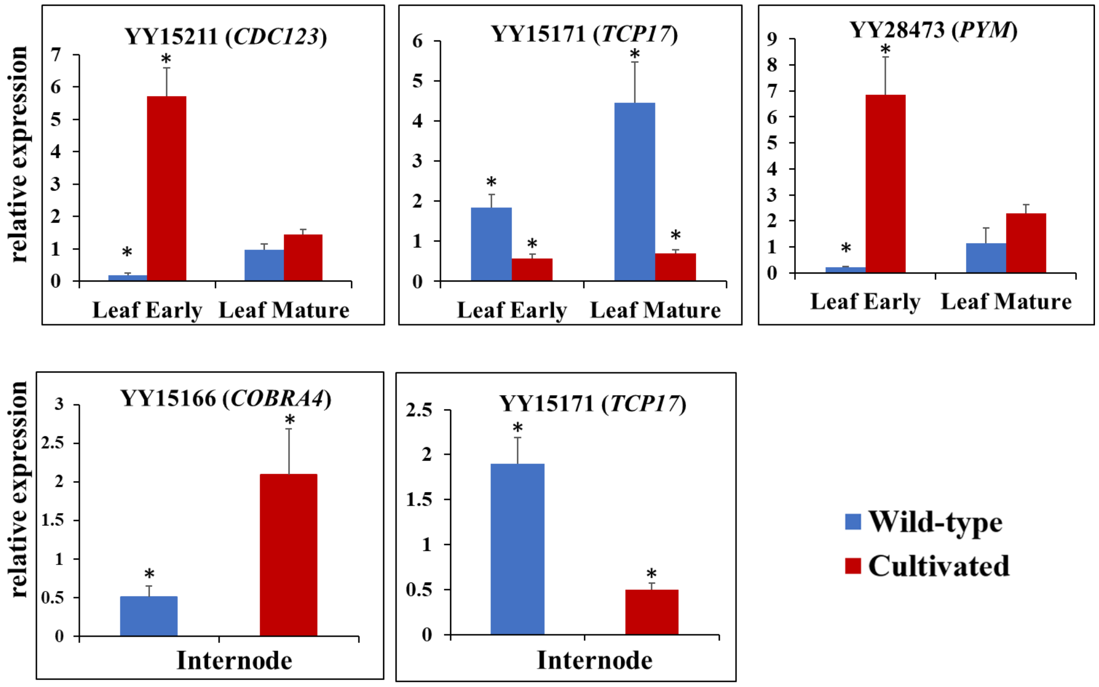

Figure S23. Relative expression of genes differentially expressed from different tissues (leaves, internode) between wild and cultivated spinach by qRT-PCR. Error bars depict standard deviation of the mean. *Denotes delta Ct values that were significantly different between spinach types ($P < 0.05$).

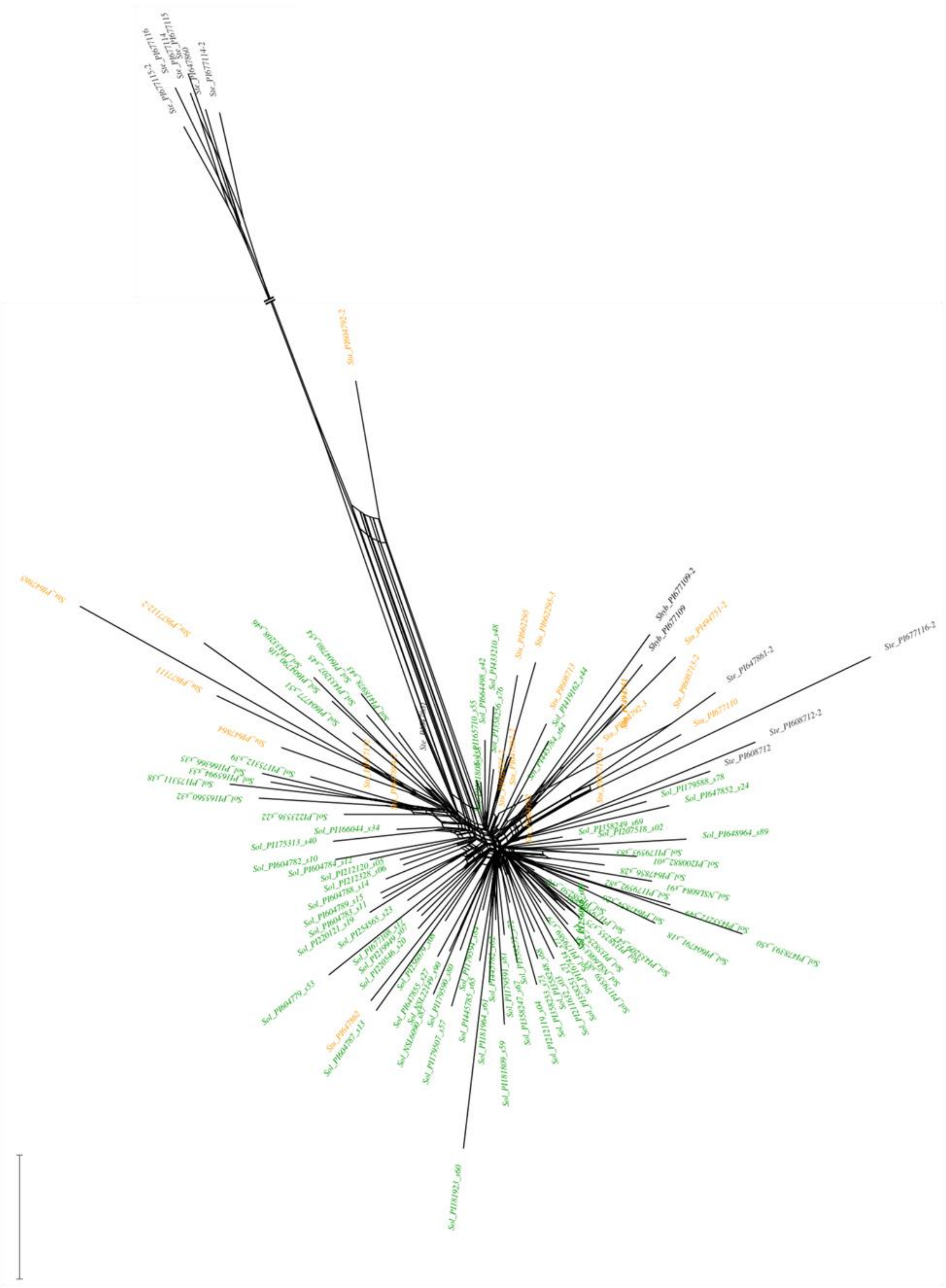

Figure S24. SplitsTree analysis of 112 *Spinacia* accessions. It includes 81 *S. oleracea* accessions (with two hybrids, green color), 20 *S. turkestanica* (brown color), and 11 *S. tetrandra* accessions (grey color)

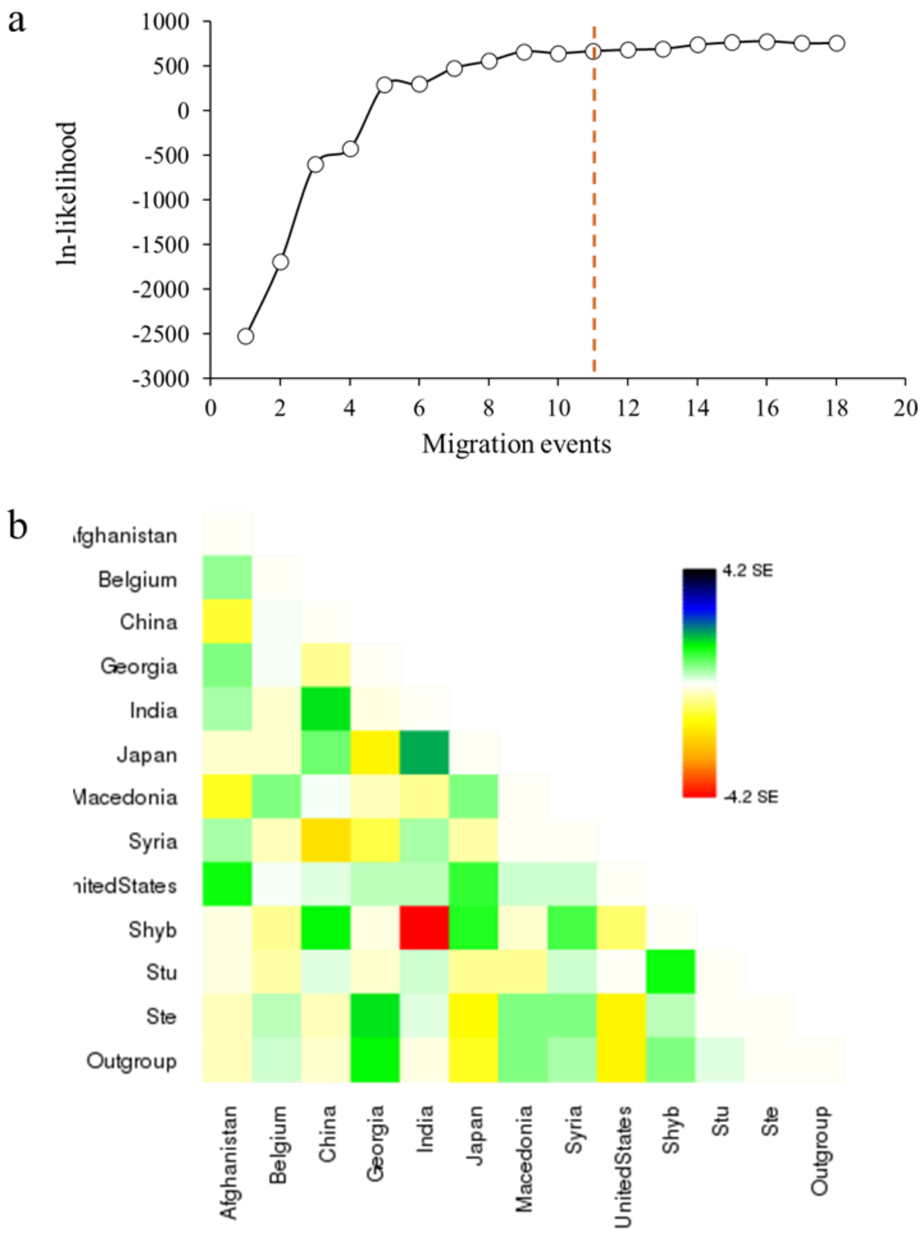

Figure S25. **a,** Ln-likelihood of for treemix analysis, showing the optimal migration events is m=11. **b,** Heatmap of covariance matrix of treemix analysis.

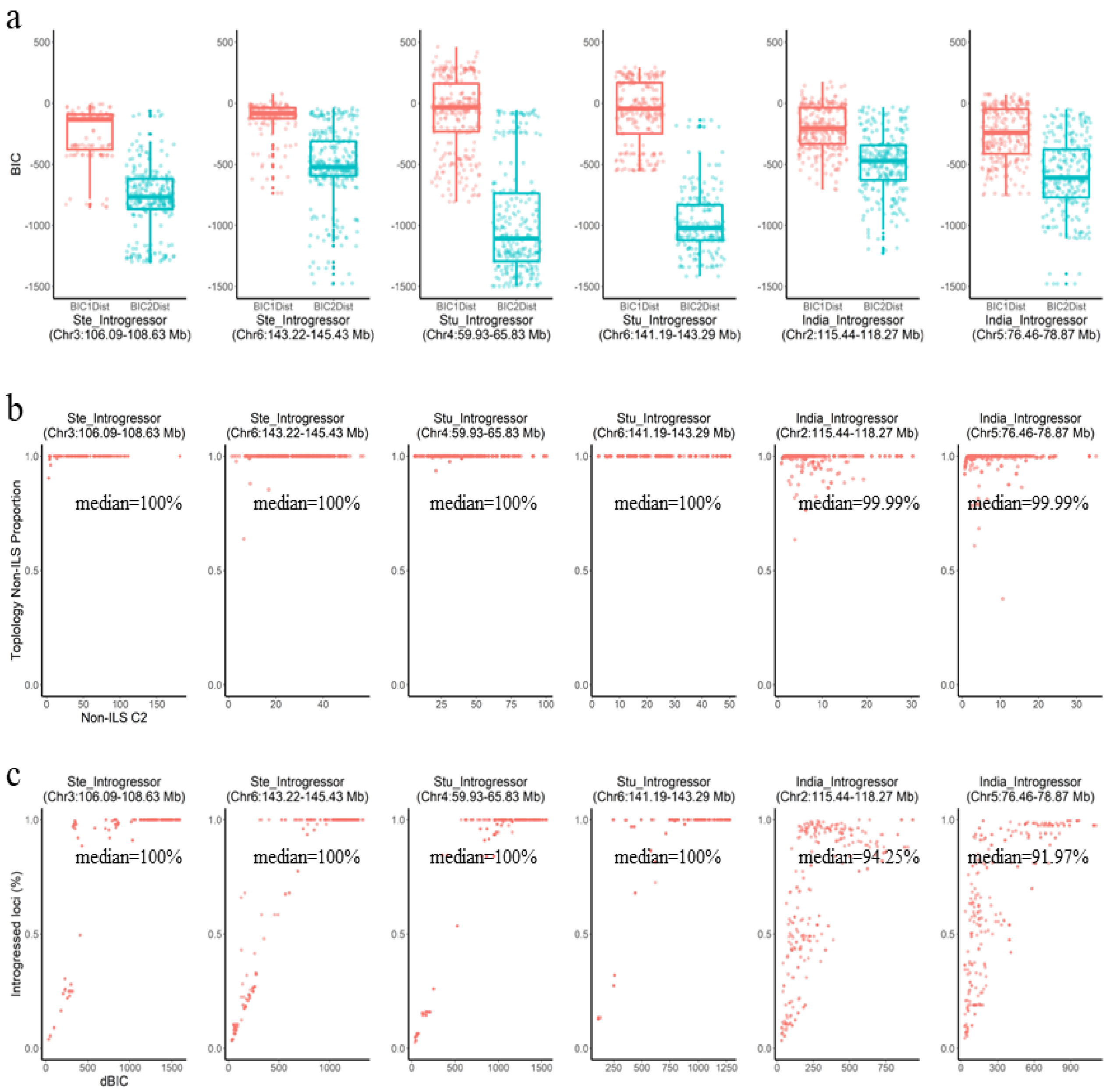

Figure S26. QuIBL output of quantifying introgression from ILS (incomplete lineage sorting) for six regions showing strong signals of introgression in *fd_M* statistics (Main text Fig 6. c-k). **a,** The BIC (a Bayesian information criterion) tests for the model of non-ILS topologies has significant lower BIC scores (with a strict cutoff of delta BIC, dBIC = BIC1st-BIC2st >30) than the model of ILS topologies for each of six introgressed regions (**Main text Fig 6. c-k**: Ste_Introgressor (Chr3:106.09-108.63 Mb); Ste_Introgressor (Chr6:143.22-145.43 Mb); Stu_Introgressor (Chr4:59.93-65.83 Mb); Stu_Introgressor (Chr6:141.19-143.29 Mb); India_Introgressor (Chr2:115.44-118.27 Mb); India_Introgressor (Chr5:76.46-78.87 Mb)). **b,** Distribution of non-ILS topologies probability showing the proportion of belonging to the non-ILS topologies is enriched in nearly 100% proportion and their positive correlation with non-ILS internal branch length (non-ILS C2). **c,** The distribution of inferred introgressed loci account for discordant loci of each triplet topologies of six regions, showing they all reach >90% (median value) proportions, and positively correlated with dBIC scores.

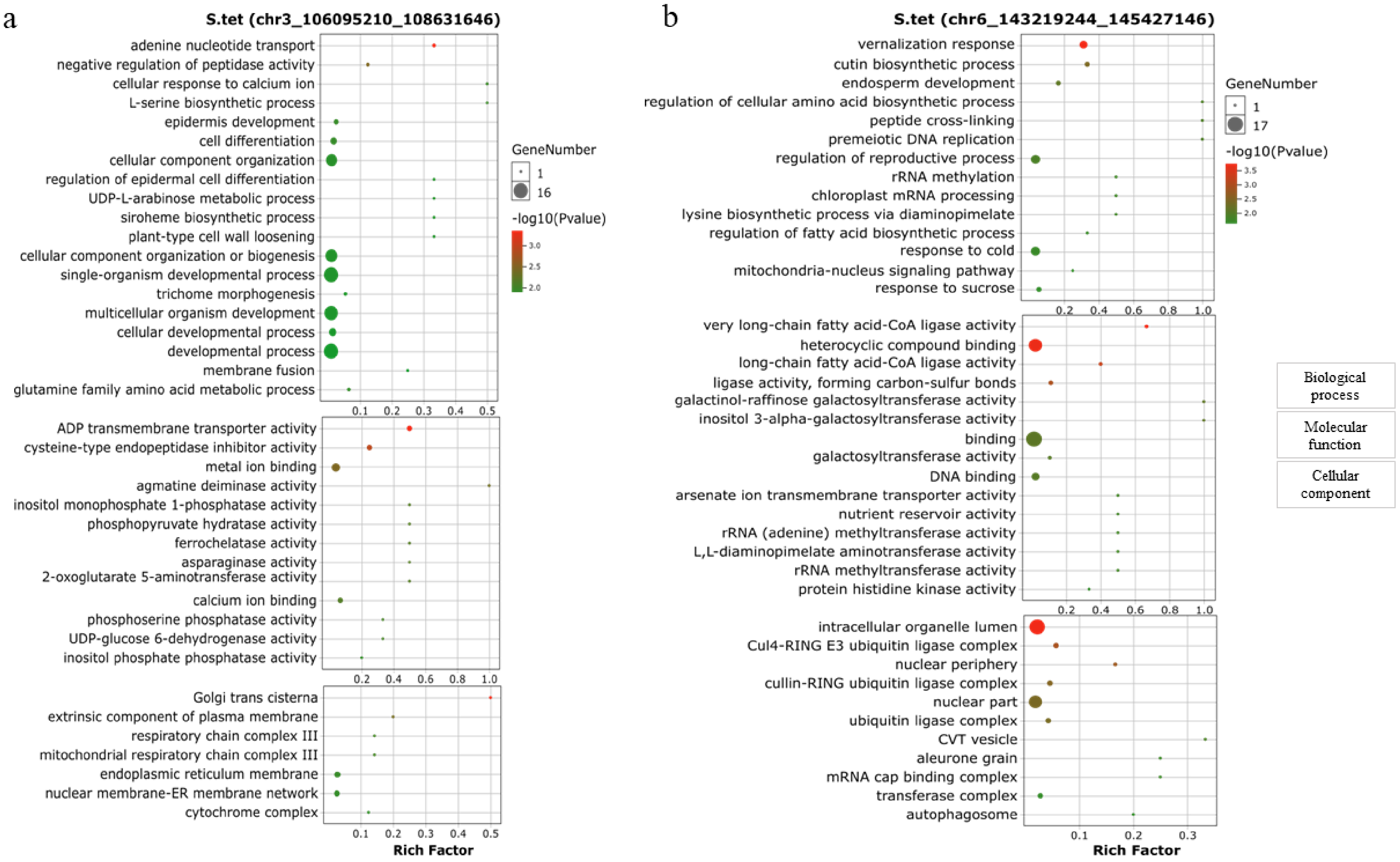

Figure S27. GO enrichments of introgressed loci in several regions with strong signals from wild species *S. tetrandra* (S.tet), *S.turkestanica* (S.tur) and India cultivars. a. S.tet (chr3_106095210_108631646). **b.** S.tet (chr6_143219244_145427146).

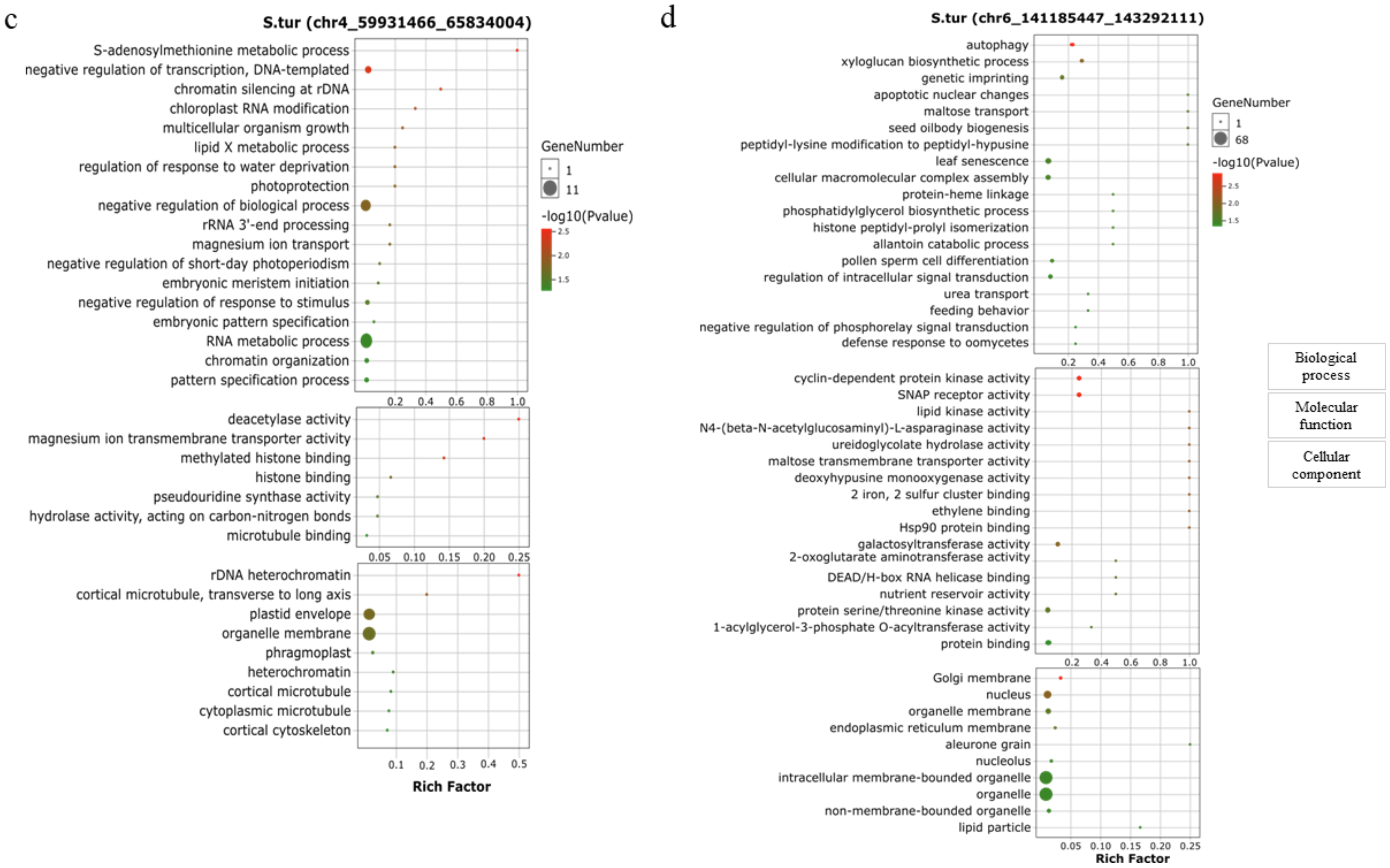

Figure S27. GO enrichments of introgressed loci in several regions with strong signals from wild species *S. tetrandra* (S.tet), *S.turkestanica* (S.tur), and India cultivars. c. S.tur (chr4_59931466_65834004). **d.** S.tur (chr6_141185447_143292111).

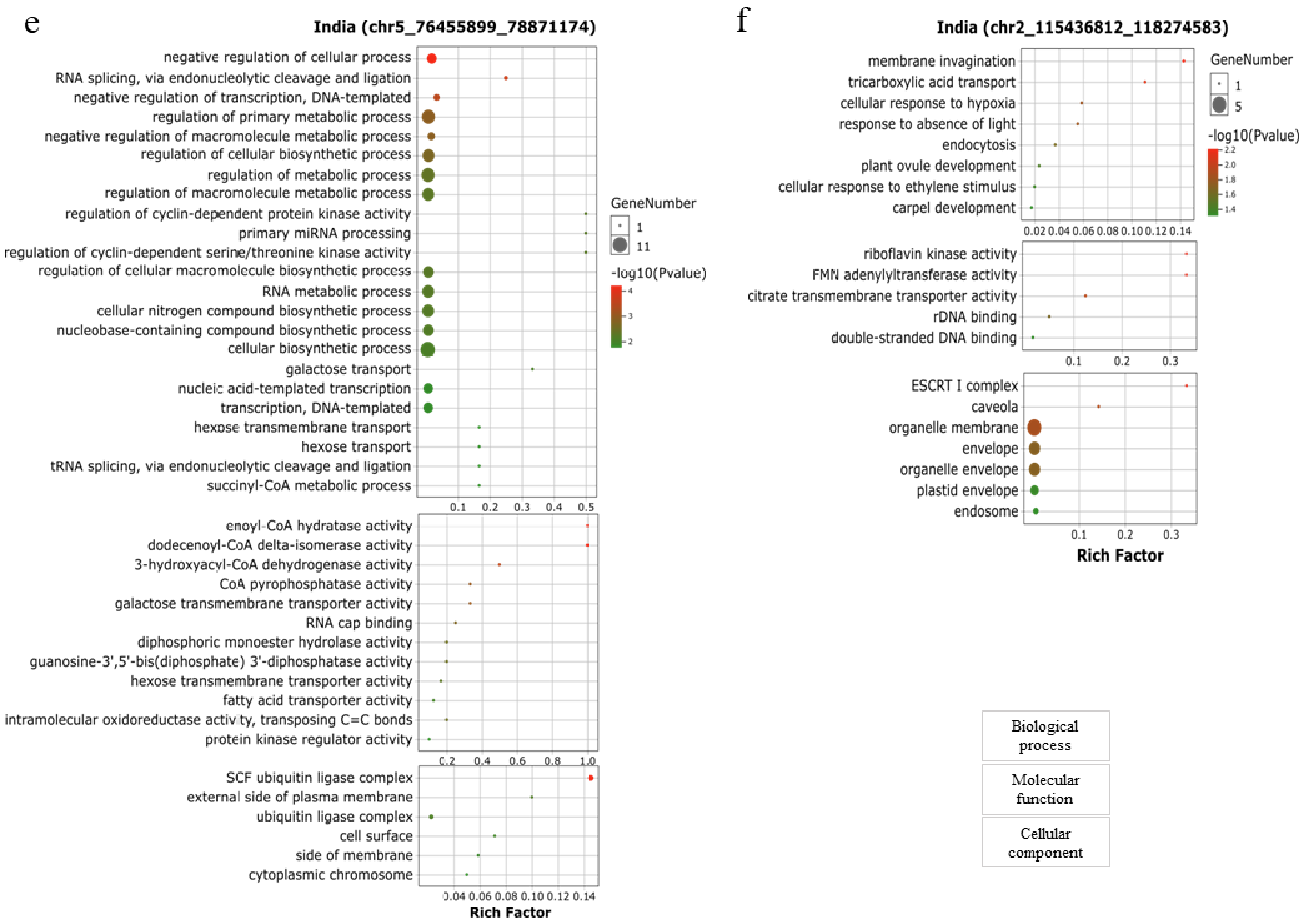

Figure S27. GO enrichments of introgressed loci in several regions with strong signals from wild species *S. tetrandra* (S.tet), *S.turkestanica* (S.tur), and India cultivars. e. India (chr2_115436812_118274583). **f.** India (chr5_76455899_78871174).

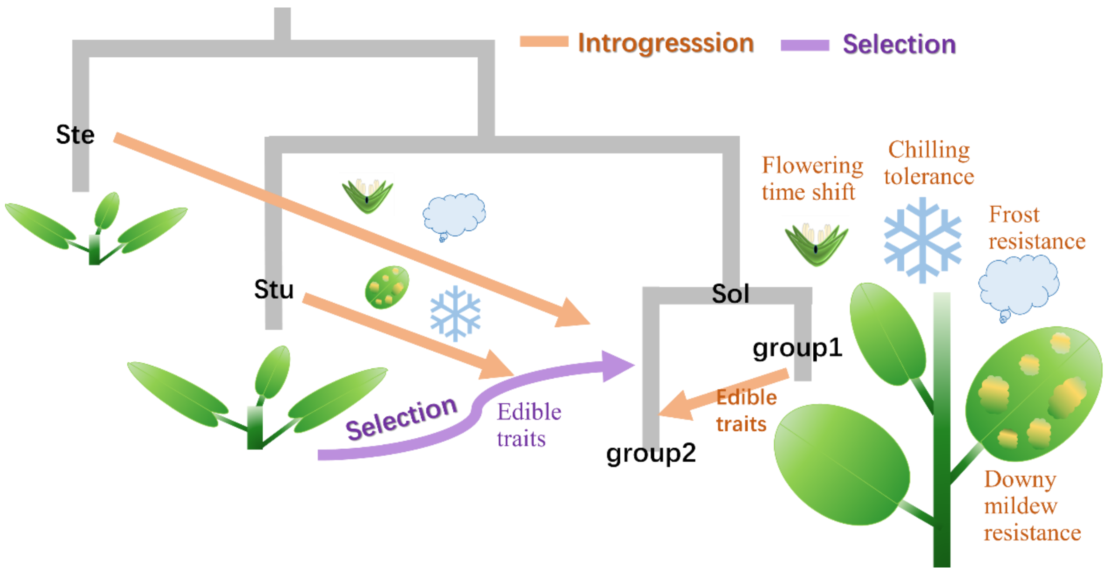

Figure S28. Proposed pathways of spinach domestication and improvement.
