# Supplementary material for: The spinach YY genome reveals sex chromosome evolution, domestication, and introgression history of the species
Source: Genome Biol. 2022 Mar 7;23:75. doi: 10.1186/s13059-022-02633-x (PMC8902716; doi:10.1186/s13059-022-02633-x)
Supplement: Supplementary file 4 — Additional file 4. Review history. [file 13059_2022_2633_MOESM4_ESM.docx]

Review History

**First round of review**

**Reviewer 1**

**Are you able to assess all statistics in the manuscript, including the appropriateness of statistical tests used?**

Yes, and I have assessed the statistics in my report.

**Comments to author:**

General comments:

I appreciate the opportunity to review the manuscript "Sex chromosome evolution, domestication and introgression history revealed by a YY genome in spinach" by Ma et al. The authors describe the sequencing and assembly of a spinach supermale individual using PacBio long-read sequencing and Hi-C data. Using a male genetic map they define the sex-determining region of spinach verifying the previously reported region on chromosome 1. By comparing the YY genome with an XX female assembly the authors further characterize the SDR highlighting Y chromosome-specific inversions and insertions. Differential expression analyses of early and late stages of female and male flowers allow the identification of genes exhibiting sex-biased or sex-specific expression. Together with co-expression network analyses these data highlight NRT1 as a candidate gene for spinach sex determination. Next, the authors set out to study the genetic basis of spinach domestication. To this end, they re-sequence 112 accession generating more than 2 million sequence variants. Selective sweep analyses identify several regions and candidate genes that might have played a role for spinach domestication. Finally, intra- and interspecific introgressions are reported.

While the manuscript contains a lot of interesting data and analyses, I often did not find the information easily accessible. The paper covers many different topics. The description of the methods, which generally appeared appropriate to me, is several times too vague. Also, the relevance of several results is not always easy to see. Finally, the manuscript seemed like two independent papers, one about sex determination and one about domestication. I think two more focused papers might make the data more accessible also considering the amount of supplementary information.

Specific comments:

Lines 77-83: classic views may not be warranted. Additional examples of sequenced and assembled SDRs include Vitis (Y chromosome), Populus alba (W chromosome) and P. trichocarpa (Y chromosome). In all these cases, using long-read sequencing, SDR assembly was straightforward. I would expect many more such examples (new data from strawberry also point to a simple SDR).

Lines 150-168: I do not understand the motivation of comparing NUPTs, miRNAs and MADS-box genes between two genome assemblies. Possible differences between the sexes will be localized in the sex-determining region. For a general comparison the analyses seem too limited.

Lines 173-176: How was the SDR delimited? The text only states that "the 547 out of 859 bins located within a 38.18 Mb (Chr1:130.91-169.09) region was defined as the SDR". How did the authors determine these exact borders? Figure S9c suggests a less clear delineation. The Tajima's D values, mentioned by the authors as a second line of evidence to define the SDR (L179-181), also do not appear to allow a clear definition of the localization of the SDR. Why did the authors not perform a GWAS for sex using the re-sequenced accession? The F1 maps could be biased by family-specific effects. A GWAS with unrelated individuals will give a more robust estimate.

Additionally, the definition of two strata does not appear to make sense to me. In my understanding, strata are defined as regions that became sex-linked at different times during the evolution of sex chromosomes. However, they do not comprise interspersed sequences and are not limited to inversions. They should be defined based on sequence divergence. Fig. 2b does not appear to support the authors definition of the different strata.

Fig. S20: It is impossible to see the differences between the groups with this representation (there is not even a Y-axis). I find it unexpected to find lower nucleotide diversity in the wild species (S. tetrandra) compared to the domesticated one.

Fig. S22: It is not possible to see all the individuals in this plot. Could it help to make the colors transparent? In the upper panel there are S. tetrandra individuals (grey points) visible in the selected part on the right. These are not visible anymore in the zoomed-in representation in the lower panel.

In general, the motivation of several analyses did not become clear to me. Just as an example, what are the implications of the relative Y expression and Fig. S15 (L226-228)? I find that the paper contains too many purely descriptive data. It would be good to add some conclusions.

Finally, the language made many sentences of the manuscript really difficult to understand for me. I believe the clarity of the paper could be markedly improved by thorough language editing.

Methodological questions:

Why was RaGOO used to group the contigs if the anchoring to the six pseudomolecules was done using Hi-C data?

How many biological replicates were analyzed in the RNA-seq experiment?

Which accessions exactly were used for the selective sweep analyses? From the phylogenetic analyses it appears clear that some of the S. tetrandra individuals are actually representatives of S. oleracea. In general, the classification of the accessions appears quite messy, which could compromise the according analyses.

In general, while the methods section is exceedingly long, I am not convinced that it actually describes all the important details needed to understand or even reproduce the analyses. For example, I was interested to better understand the delineation of the SDR (see comment above). The main manuscript refers to the Additional file (L520). However, in the according section of the additional file, the exact same paragraph is simply repeated. This only makes the additional file even longer but does not help to understand the methods.

Minor comments:

Line 73: Dioecy occurs in ~6% of angiosperm species

Lines 86-87: perhaps I mis-read the willow paper, but to me it seemed to point to the same single factor system as in poplar.

**Reviewer 2**

**Are you able to assess all statistics in the manuscript, including the appropriateness of statistical tests used?**

Yes, and I have assessed the statistics in my report.

**Comments to author:**

The origin and evolution of sex chromosomes has always been a hot spot in biological research. In this manuscript, the authors assembled a high-quality YY genome and identified the sex determination regions (SDRs) of spinach. They also conducted population analysis to study the domestication history and selection signals associated with several traits during spinach domestication and improvement. The conclusion of this article is novel, but there are many problems in writing of the manuscript. My comments are as follows:

1.The background of the article introduced that sex chromosomes may originate from autosomes by either two mutation or single mutation models, and further degenerate and differentiate (Line 73-79). However, in the results of this article, there is no strong evidence to support which model spinach belongs to. In contrast, the highlight of this article is to avoid the difficulty of assembling SDRs by sequencing an individual with YY genotype. Therefore, I strongly recommend that the author introduce more SDR assembly and comparison in the background of the article, rather than the model of sex determination mechanism.

2.The description of willow SDR in lines 85-88 requires references, and there is no clear report that there are 'two male-specific candidate sex-determination genes' in willow. In addition, the descriptions of whether poplar sex determining genes are one or two contradict each other. As mentioned above, these are misleading to readers and are not the focus of this article. Therefore, the author is recommended to modify these descriptions.

3.The reference to the chart in the article is very confusing, and there are many obvious errors, such as: lines 140-142; line 145; lines 151-157 and so on (too many). The description in lines 156-157 is inaccurate and should be consistent with the description in Fig. S5.

4.Line 171-173, the authors should explain why the genetic map has such a large difference in the number of markers between male and female.

5.Line 173-174, the authors should use a figure to show the distribution of the gender co-segregation bins on the chromosomes, because this distribution is related to the definition of the SDR range.

6.Line 176, the lines and colors in fig2a and 2b are difficult to distinguish.

7.Line 179-181, the author should explain why tajima's D can be used as another evidence to define SDR. In addition, in fig s9d, one region on chr4 also showed a similar situation.

8.Due to the large number of repetitive sequences, it is difficult to assemble SDRs. The accuracy of the assembly of the inverted and the inserted regions in Y chromosome need to be further confirmed by the alignment of pacbio long reads.

9.Line 211 should be group4 and grou8 with both inversion and insertions from the SDR. And the number of the LTR-RT elements in each group should be displayed in fig. 2f.

10.It is recommended to add a scale of expression in Fig 3d. In addition, the expression genes related to GA signaling is low in both males and females in the early stage. This result affects the inference of sex determination pathways. Whether there are reports of NRT1 homologous gene mutations affecting stamen development in Arabidopsis to further support the sex determination function of this gene. Will other genes in the same transcription family as NRT1 compensate for the loss of NRT1?

11.The time described in line 291 does not match Figure 4e.

12.The analysis and results of population genetics are relatively routine. What is curious is how the author distinguishes the introgression from wild populations from the incomplete lineage sorting in the domestication process? I don't think the ABBA test can rule out the influence of ILS, especially for domesticated populations, where the differentiation of the population structure is prone to false positive results. Another question is, since it is mentioned in this article that there are many structural variations and differences in gene content between the YY and XX genomes, whether the use of different genotypes as reference genomes will affect the analysis of population genetics, especially the selected genes and introgressed regions?

**Authors Response**

**Point-by-point responses to the reviewers’ comments:**

Reviewer 1#

General comments:

I appreciate the opportunity to review the manuscript "Sex chromosome evolution, domestication and introgression history revealed by a YY genome in spinach" by Ma et al. The authors describe the sequencing and assembly of a spinach supermale individual using PacBio long-read sequencing and Hi-C data. Using a male genetic map they define the sex-determining region of spinach verifying the previously reported region on chromosome 1. By comparing the YY genome with an XX female assembly the authors further characterize the SDR highlighting Y chromosome-specific inversions and insertions. Differential expression analyses of early and late stages of female and male flowers allow the identification of genes exhibiting sex-biased or sex-specific expression. Together with co-expression network analyses these data highlight NRT1 as a candidate gene for spinach sex determination. Next, the authors set out to study the genetic basis of spinach domestication. To this end, they re-sequence 112 accession generating more than 2 million sequence variants. Selective sweep analyses identify several regions and candidate genes that might have played a role for spinach domestication. Finally, intra- and interspecific introgressions are reported.

While the manuscript contains a lot of interesting data and analyses, I often did not find the information easily accessible. The paper covers many different topics. The description of the methods, which generally appeared appropriate to me, is several times too vague. Also, the relevance of several results is not always easy to see. Finally, the manuscript seemed like two independent papers, one about sex determination and one about domestication. I think two more focused papers might make the data more accessible also considering the amount of supplementary information.

*Response: We appreciate your time and expertise and your comments helped us to improve our manuscript.*

*We have worked on several genome projects, including papaya, pineapple, and sugarcane genome, and each genome manuscript covers two to four topics. Such suggestions have been brough up a few times. We consulted with editors and they want us to include all topics to make the genome manuscript more robust and substantial. For this reason, we are keeping both topics. Thank you for recognizing the large amount of work we have put on this manuscript.*

*In this revised version, we have re-wrote and added details for several methods to make them more accessible and readable. We also rearranged the results section to make it more logical and readable. For example, we have shifted NUPTs and miRNA analyses to sex-chromosome part, since this is related to sex-chromosomes evolution and sex-differentiation. We have also re-wrote other parts in the main text to improve clarity. Also, some contents that are less related to the main topics were discarded, including dosage compensation and gene family analysis. Two co-authors who are native English speakers polished the English of the manuscript.*

Specific comments:

Lines 77-83: classic views may not be warranted. Additional examples of sequenced and assembled SDRs include Vitis (Y chromosome), Populus alba (W chromosome) and P. trichocarpa (Y chromosome). In all these cases, using long-read sequencing, SDR assembly was straightforward. I would expect many more such examples (new data from strawberry also point to a simple SDR).

*Response: We have revised the sentences to make them clear, and have added the description for sex chromosomes assemblies including Vitis (Y chromosome) (Picq et al. 2014), P. trichocarpa (Y chromosome) (Geraldes et al. 2015), and strawberry (Tennessen et al. 2018) along with other species that reported evidence of the early stage of sex chromosome evolution.*

*References Geraldes, A., et al. (2015). Recent Y chromosome divergence despite ancient origin of dioecy in poplars (Populus). Mol Ecol. 24:3243-3256.*

*Picq, S., et al. (2014) A small XY chromosomal region explains sex determination in wild dioecious V. vinifera and the reversal to hermaphroditism in domesticated grapevines. BMC Plant Biol. 14:1-17. Tennessen, JA., et al. (2018) Repeated translocation of a gene cassette drives sexchromosome turnover in strawberries. PLoS Biol. 16:e2006062.*

Lines 150-168: I do not understand the motivation of comparing NUPTs, miRNAs and MADS-box genes between two genome assemblies. Possible differences between the sexes will be localized in the sex-determining region. For a general comparison the analyses seem too limited.

*Response: NUPTs and miRNAs play an important role in sex chromosome evolution in plants (Cheng et al. 2019; Aryal et al. 2014). As suggested by reviewer, we analyzed NUPTs, miRNAs contents in the sex-chromosome (X and Y), sex-determination region (SDR) and its X counterpart. Highest density on NUPTs were detected within a 112 kb on Y and a 129 kb region on X chromosome, but not in SDR region. Besides, miRNAs were enriched at intergenic regions of Y chromosome (15 YY-specific) and X chromosome (9 X-specific) demonstrating their involvement in sex chromosome evolution. Therefore, we moved these contents to Results section “Comparative analysis of SDR and its X counterpart” Line 221-227.*

*References*

*Cheng, G., et al. (2019). Bioinformatics Analysis and Chromosome Location of Nuclear Integrants of Plastid DNA in Asparagus officinalis. Chinese Bulletin of Botany, 54(3): 328.*

*Aryal, R., et al. (2014). Sex specific expression and distribution of small RNAs in papaya. BMC Genomics. 15(1): 1-10.*

Lines 173-176: How was the SDR delimited? The text only states that "the 547 out of 859 bins located within a 38.18 Mb (Chr1:130.91-169.09) region was defined as the SDR". How did the authors determine these exact borders? Figure S9c suggests a less clear delineation. The Tajima's D values, mentioned by the authors as a second line of evidence to define the SDR (L179-181), also do not appear to allow a clear definition of the localization of the SDR. Why did the authors not perform a GWAS for sex using the re-sequenced accession? The F1 maps could be biased by family-specific effects. A GWAS with unrelated individuals will give a more robust estimate.

*Response: Thanks for proposing using GWAS as an alternative approach to map and define sex-determining region. In the current version, we used re-sequencing data of 70 accessions (26 females and 44 males) along with sex phenotype to perform genome-wide association studies (GWAS). (Fig. 1c & 1d; Methods Line 532-538, and Results Line 178-180). We also performed other analyses and integrated all the evidence for defining the sex determination region (SDR). i) The density distribution of sex co-segregating bins (100-kb window). ii) GWAS Manhattan plot of two sexual phenotypes (26 females and 44 males re-sequencing data); iii) male-specific SNPs along chromosome; iv) genetic differentiation (Fst) between females and males; v) Tajima’s D of males and females. All these analyses together overlapped in a region of Y chromosome Chr1: 130.91-169.09Mb (~38.18Mb), which was clearly defined as the SDR. Please see the new results at Line 177-186 and Fig.1c & 1d.*

*SDR was comprised of contigs containing sex co-segregating markers (heterozygous marker genotypes in all 41 males and homozygous in all 39 females). The SDR was genetically mapped by screening sex co-segregating markers and then physically defined by retrieving contig IDs of these markers. These contigs were further matched to the Y chromosome assembly and two contigs located at two ends were proposed as the boarder of SDR.*

*We found that 859 sex co-segregating bins were distributed among six chromosomes, but 547 of these bins were mapped in Y chromosomes with a higher density compared with the rest of sex co-segregating contigs that were anchored to autosomes or two ends of the Y chromosome (Fig. 1c & 1d). This inconsistency might be attributed to artifact introduced by maximum likelihood mapping algorithm.*

*These 547 contigs overlapped with genomic region defined by GWAS, male-specific SNPs, genetic differentiation (Fst) between females and males, as well as Tajima’s D score between females and males (Fig. 1c & 1d). These 547 contigs were selected to define the physical location of SDR in the Y chromosome.*

*The final boundaries of SDR (Chr1:130.91-169.09Mb) was determined based on position of the sex co-segregating contig “tig0015356” (130,902,560-130,946,157 bp) as the left boarder of SDR and contig “tig0016281” (169,055,186-169,092,663) bp as the right boarder of SDR from the anchored Y chromosome (Fig. 1c & 1d) . We revised content in manuscript accordingly at Line 168-186 in the revised version.*

Additionally, the definition of two strata does not appear to make sense to me. In my understanding, strata are defined as regions that became sex-linked at different times during the evolution of sex chromosomes. However, they do not comprise interspersed sequences and are not limited to inversions. They should be defined based on sequence divergence. Fig. 2b does not appear to support the authors definition of the different strata.

*Response: We agree that evolutionary strata are formed at different time when recombination ceased and the regions become sex co-segregatting (Ray Ming discussed with Deborah Charlesworth about the term sex-linked that was often used in publications of sex chromosome research. By definition of linkage groups, sex-linked regions includes the entire sex chromosomes, not just the SDR. Deborah and Ray agreed that sex co-segregating is more appropriate to describe the non-recombining region of sex chromosomes. Hope you don’t mind that we make this clarification.).*

*Indeed the causes of recombination suppression are not limited to inversions. Other mechanisms include insertions, deletions, and DNA methylation. Inversions, deletions, and insertions have been documented in sex chromosomes as the causes of recombination suppression, but it is questionable whether DNA methylation can independently cause recombination suppression permanently.*

*In spinach SDR, four inversions were detected. The largest inversion is the most ancient, about 1.92 Mya, likely triggering sex chromosome evolution in spinach. The other three small inversions occurred at 1.08, 0.87, and 0.68 Mya, but these estimates of divergence time were not significantly different, which is the reason we initially designated as stratum 2a, 2b, and 2c. You brough up an important point that these three inversions are dispersed and shouldn’t be designated as one stratum. Now we felt it is more reasonable to designated them as three strata: stratum 2, stratum 3, and stratum 4 (Fig. 2b and 2d). We appreciate your input.*

Fig. S20: It is impossible to see the differences between the groups with this representation (there is not even a Y-axis). I find it unexpected to find lower nucleotide diversity in the wild species (S. tetrandra) compared to the domesticated one.

*Response: Sorry for such an unreadable Figure. We have remade the figure and added the Y-axis to ensure it is readable. We have also changed the figure number from previous Figure S20 to the current Figure S16.*

Fig. S22: It is not possible to see all the individuals in this plot. Could it help to make the colors transparent? In the upper panel there are S. tetrandra individuals (grey points) visible in the selected part on the right. These are not visible anymore in the zoomed-in representation in the lower panel.

*Response: Sorry for such an unreadable figure. We have replotted it and make the colors transparent in a zoomed-in panel. After revising the manuscript, figure number is changed to Fig. S18.*

In general, the motivation of several analyses did not become clear to me. Just as an example, what are the implications of the relative Y expression and Fig. S15 (L226-228)?

*Response: Calculating Y-linked gene expression relative to autosomal gene expression enabled the identification of potential dosage compensation. Although the spinach X/Y sex chromosomes diverged recently, the down-regulated Y-linked genes compared with PAR and rest chromosomes indicated the degrading Y chromosome after ceased recombination, as observed in papaya and white campion (Papadopulos et al., 2015; Liu et al., 2021; Muyle et al., 2017). However, considering the content and overall structure for presenting results and discussion of manuscript, we removed this part in current revised manuscript.*

*References: Papadopulos, A.S., et al. (2015). Rapid Y degeneration and dosage compensation in plant sex chromosomes. Proc Natl Acad Sci USA. 112: 13021–13026. Liu, J., et al. (2021). Transcriptional regulation of dosage compensation in Carica papaya. Sci. Rep. 11: 1–9.*

*Muyle, A., Shearn, R., and Marais, G.A. (2017). The Evolution of Sex Chromosomes and Dosage Compensation in Plants. Genome Biol Evol. 9: 627–645.*

I find that the paper contains too many purely descriptive data. It would be good to add some conclusions.

*Response: We have add conclusions in relevant parts of the manuscripts.*

Finally, the language made many sentences of the manuscript really difficult to understand for me. I believe the clarity of the paper could be markedly improved by thorough language editing.

*Response: The language has been edited and polished by two co-authors, Dr. Will Wadlington and Dr. Amanda M. Hulse-Kemp, who are native English speakers.*

Methodological questions:

Why was RaGOO used to group the contigs if the anchoring to the six pseudomolecules was done using Hi-C data?

*Response: There are two considerations for using RaGOO for grouping. 1. Directly using HiC grouping may generate chimeric scaffolds due to noisy Hi-C signals. Even though Hi-C technology is capable of scaffolding a wide range of genomes, it has limited power to solve complex sequences because short Hi-C sequencing reads are prone to unambiguous mapping against repetitive sequences, leading to mis-joined scaffolds or chimeric chromosomes. 2. RaGOO enabled the assembly of contigs into scaffolds based on a well-assembled reference genome. The reference-guided assembly approach largely reduces false positive as shown in our first scenario. It could assign contigs according to the reference genome if the target genome and reference genome are in the same species, as used by many studies (Alonge et al. 2019). Therefore, the strategy of initial grouping the contigs using RaGOO and then ordering using HiC data will be the best way to anchoring YY contigs into six pseudomolecules.*

*Reference:*

*Alonge, M., et al. (2019). RaGOO: fast and accurate reference-guided scaffolding of draft genomes. Genome Biol. 20(1): 1-17.*

How many biological replicates were analyzed in the RNA-seq experiment?

*Response: In RNA-seq experiment, for identification of sex determination/differentiation genes, three biological replications of each type of selected samples (five different stages of flower development S1-S5) were collected with a sufficient sample size of 5- 15 flowers.*

Which accessions exactly were used for the selective sweep analyses? From the phylogenetic analyses it appears clear that some of the S. tetrandra individuals are actually representatives of S. oleracea. In general, the classification of the accessions appears quite messy, which could compromise the according analyses.

*Response: Sorry for such confusion. Before selective sweep analysis, we have purged accessions showing potentially misclassified identities in population structure and phylogenetic analysis. We have added a new column in Table S12 to show the accessions used in selective sweep analysis.*

In general, while the methods section is exceedingly long, I am not convinced that it actually describes all the important details needed to understand or even reproduce the analyses. For example, I was interested to better understand the delineation of the SDR (see comment above). The main manuscript refers to the Additional file (L520). However, in the according section of the additional file, the exact same paragraph is simply repeated. This only makes the additional file even longer but does not help to understand the methods.

*Response: Sorry for such confusion. We have added more details in the method section to make it clear and removed redundant and unnecessary content in the revised manuscript. We have addressed how SDR was defined by integrating evidence from genetic maps, GWAS mapping, Fst and Tajima’s D analyses,* and *profiling of distribution malespecific SNPs. See detailed in response above We also apologize for the redundant information in the additional file and have removed them in the current version.*

Minor comments:

Line 73: Dioecy occurs in ~6% of angiosperm species.

*Response: Corrected.*

Lines 86-87: perhaps I mis-read the willow paper, but to me it seemed to point to the same single factor system as in poplar.

*Response: Yes, you are right. We have corrected the mistakes.*

Reviewer 2#:

The origin and evolution of sex chromosomes has always been a hot spot in biological research. In this manuscript, the authors assembled a high-quality YY genome and identified the sex determination regions (SDRs) of spinach. They also conducted population analysis to study the domestication history and selection signals associated with several traits during spinach domestication and improvement. The conclusion of this article is novel, but there are many problems in writing of the manuscript. My comments are as follows:

*Response: We appreciate your time and expertise and your comments helped us to improve our manuscript.*

1.The background of the article introduced that sex chromosomes may originate from autosomes by either two mutation or single mutation models, and further degenerate and differentiate (Line 73-79). However, in the results of this article, there is no strong evidence to support which model spinach belongs to. In contrast, the highlight of this article is to avoid the difficulty of assembling SDRs by sequencing an individual with YY genotype. Therefore, I strongly recommend that the author introduce more SDR assembly and comparison in the background of the article, rather than the model of sex determination mechanism.

*Response: We agree with the reviewer’s point and have changed the description. We have introduced more SDR assemblies of published species and have emphasized how YY genotype viability enables the study of early-stage sex chromosome evolution. Meanwhile, we have also reduced the descriptions of sex-determination by two- and one- gene mutation models and make background more relavent to the manuscript content as suggested.*

2.The description of willow SDR in lines 85-88 requires references, and there is no clear report that there are 'two male-specific candidate sex-determination genes' in willow. In addition, the descriptions of whether poplar sex determining genes are one or two contradict each other. As mentioned above, these are misleading to readers and are not the focus of this article. Therefore, the author is recommended to modify these descriptions.

*Response: Yes, you are right. We have corrected this mistake. Populus genus has featured one-factot in Populus tremula in and two- factor model in P. tremula for sex determination suggest that dioecy may have evolved independently in different poplar subgenera (Xue et al., 2020; Muller et al., 2020). We listed respective subgenera names to prevent confusion in the current version. In several willow species (Salix: Zhou et al., 2020) the same basic sex-determining system appears to be used as in P. deltoides (discussed in Müller et al., 2020). Further we have changed the description according to the suggestion in previous comment.*

*References*

*Xue, L., et al. (2020). Evidences for a role of two Y-specific genes in sex determination in Populus deltoides. Nat. Commun. 11: 1–12.*

*Muller, N.A., et al. (2020). A single gene underlies the dynamic evolution of poplar sex determination. Nat Plants. 6: 630–637.*

*Zhou,. R, et al. (2020). A willow sex chromosome reveals convergent evolution of complex palindromic repeats. Genome Biol. 2020, 21:1-19.*

3.The reference to the chart in the article is very confusing, and there are many obvious errors, such as: lines 140-142; line 145; lines 151-157 and so on (too many). The description in lines 156-157 is inaccurate and should be consistent with the description in Fig. S5.

*Response: Sorry for such confusion. We have corrected the citation numbers for Figures and Tables.*

4.Line 171-173, the authors should explain why the genetic map has such a large difference in the number of markers between male and female.

*Response: For F1 genetic mapping, only SNPs with homozygous alleles from the female parent and heterozygous alleles for male parent were filtered out to build male genetic map, vice versa for female genetic map. Thus, the numbers of markers for two maps are determined by homozygosity of two parents. In this study, a total of 369,524 SNPs were generated for male genetic map while only 41,876 are kept for female map construction after removal of loci with lower quality (quality scores <1500). This result indicated that the female parent (Viroflay) is much more homozygous compared with the male parent (Cornell. NO-9). It matches the expectation that ‘Viroflay’ female parents were generated by self pollination of the monoecious phenotype (plants with both male and female flowers in ‘XX’ genotype) (Janick and Stevenson, 1955b), but the ‘Cornell. NO-9’ male parents were generated by out-crossing between plants with pure female and male flowers (‘XX’ and ‘XY’ genotypes respectively). We explained this difference from Line 168-176.*

5.Line 173-174, the authors should use a figure to show the distribution of the gender co-segregation bins on the chromosomes, because this distribution is related to the definition of the SDR range.

*Response: The distribution of sex co-segregating bins in six pseudomolecules were presented in FigS9C of previous version. Now to make the evidence clear, we move and integrate this figure into Fig 1c & 1d as a Main Figure in the revised version. In new Fig1c and 1d, except for showing the gender co-segregation bins, we also have integrated other evidence of defining SDR region, for example, GWAS Manhattan plot of two sexual phenotypes; distribution of male-specific SNPs; Fst between females and males; Tajima’s D of males (red line) and females (blue line).*

6.Line 176, the lines and colors in fig2a and 2b are difficult to distinguish.

*Response: We have improved the resolution of lines and switch colors with better contrast in the current Fig 2a and Fig 2b.*

7.Line 179-181, the author should explain why tajima's D can be used as another evidence to define SDR. In addition, in fig s9d, one region on chr4 also showed a similar situation.

*Response: The Tajima’s D can be used as another evidence to define SDR because of the divergence of females and males allele sites in SDR regions. In SDR region, Males (XY) with two heteromorphic homologous chromosomes have more diversified sites (more heterozygous haplotypic sites) than females (XX), which have two homomorphic homologous chromosomes. So, the Tajima’s D of males will be positively higher than that of females. There is another region on chr4 with similar situation that maybe caused by high heterozygous sites of this region that diverged between females and males. However, our definition of SDR region not only depends on Tajima’s D, but also using new evidence: i) The density distribution of sex-linked contigs (e.g. sex cosegregating bins) (100-kb window). ii) GWAS Manhattan plot of two sexual phenotypes (26 females and 44 males re-sequencing genomes); iii) male-specific SNPs along chromosome; iv) genetic differentiation (Fst) between females and males; v) Tajima’s D of males and females. All these analyses together overlapped a region at Y chromosome Chr1: 130.91-169.09Mb (~38.18Mb) which was finally clear definition as the SDR region. Please refer to Fig 1c & 1d.*

8.Due to the large number of repetitive sequences, it is difficult to assemble SDRs. The accuracy of the assembly of the inverted and the inserted regions in Y chromosome need to be further confirmed by the alignment of pacbio long reads.

*Response: We have performed the analysis to address this concern as follows: Initially, PacBio reads were aligned to six pseudomolecules of YY genome. We further calculated coverage of mapped reads across the SDR in a 6Kb window size. Particularly, we defined edges of inversions (insertions) to collinear regions based on terminal genes from respective regions. The minimum depth, maximum depth, average reads depth, and median depth across five insertions, four inversions, and edges of these regions which flank to neighboring collinear regions were statistically characterized ( refer to Methods section “Genomic analysis between SDR and X counterpart” ) (Table S9). Also, we visualized mapping depth of edges of inversions and edges of insertions by IGV browser (Fig S5). From each 6Kb window, we found that edges scanned above were covered by an average of 56.24 reads, and inversions (insertions) were covered by an average of 86.04 reads (Results: Line 206-212). Thus, the definition of inverted and inserted regions from SDR should be less prone to artifacts from assembly.*

9.Line 211 should be group4 and grou8 with both inversion and insertions from the SDR. And the number of the LTR-RT elements in each group should be displayed in fig. 2f.

*Response: Thanks for correcting the errors. We have updated the figure panel by labeling numbers of LTR-RTs from each group and make pairwise comparisons from each group of the X and Y chromosome.*

10.It is recommended to add a scale of expression in Fig 3d. In addition, the expression genes related to GA signaling is low in both males and females in the early stage. This result affects the inference of sex determination pathways. Whether there are reports of NRT1 homologous gene mutations affecting stamen development in Arabidopsis to further support the sex determination function of this gene. Will other genes in the same transcription family as NRT1 compensate for the loss of NRT1?

*Response: We have added the scale of expression in Fig 3d. Gibberellin (GA) and jasmonates (Jas) are two important hormones that play important role in various physiological events including flower induction and development especially stamen development. Gibberellin (GA) induces sex conversion in spinach. Recently, Nicholas et al. 2017 has reported that a DELLA family gene, GIBBERELLIC ACID INSENSITIVE (SpGAI) modulates GA level and further control the sex differentiation of spinach by regulating B-class genes (stamen identity). In our study, YY25178 (SpGAI-DELLA) showed female-biased expression at stage 1 can be seen in Fig 3d while other GA-signaling GID1 genes showed sex-biased expression from stage 2-3. It might possible that GA functions later to sex determination but the sex reversal of spinach female SpGAI mutant (same gene YY25178) as reported by (West et al. 2017) suggest that GA genes can’t be ignored at this point. It might possible that both JA and GA synergically or through independent pathways regulate sex determination. Further study needs to be conducted to confirm the functions of both hormones. NRT1/PTR FAMILY (NPF) proteins were originally identified as nitrate or di/tri-peptide transporters. Recent studies revealed that this transporter family also transports the plant hormones auxin (indole-3-acetic acid), abscisic acid (ABA), and gibberellin (GA). NRT1.1/NPF6.3, AIT3/NPF4.1, AtGTR1/NPF2.10 from same transporter family members that have reported their role in auxin, ABA, ABA_GA, GA_JA transport, respectively. While another member of this family jasmonate-responsive GTR1 transporter is required for gibberellin-mediated stamen development in Arabidopsis and its knockdown mutant appeared with defective stamen due to lack of jasmonic acid (JA) and gibberellin (GA) transport, and none of the other genes have been reported to compensate this response. In our study, SDR YY-specific NRT1/PTR 6.4 (YY20280) belongs to the same family proteins and was found to have very strong male-specific expression confirmed by qPCR.*

*Further, we mine the NRT1 genes in spinach genome and identify 45 genes, with four genes on sex chromosome (following Figure provided), Most of the NRT1 family genes showed male-biased at late stage (S4-5) of flower development that further suggest this family role in stamen maturation. NRT1/PTR 6.4 is the only Y-specific gene in SDR region from this family with significant male-biased expression throughout male flower development. While few genes (YY07950, YY04223, YY10540) showed female-biased expression but none of these genes were on sex-chromosome, however, no evidence has been reported for their function in gynoecium development. According to expression profiling of NRT1 family genes, non-significant male-biased expression of genes at early-stage of flower development indicates that other genes in the same family might not compensate for the loss of Y-specific sex determinant candidate NRT1/PTR 6.4 (YY20280, red asterisk in following Figure). But it is too early to say at this stage and further transgenic studies need to be conducted to confirm this hypothesis.*

Figure. Row scale expression pattern (FPKM) of genome-wide NRT1 genes in male (M) and female (F) flower at five development stages (S1-S5). * indicate genes present on sex chromosome.

*Reference*

*West, NW., and Golenberg, EM. (2018). Gender-specific expression of GIBBERELLIC ACID INSENSITIVE is critical for unisexual organ initiation in dioecious Spinacia oleracea. New Phytol. 3: 1322–34.*

*Saito, H., et al. (2015). The jasmonate-responsive GTR1 transporter is required for gibberellin-mediated stamen development in Arabidopsis. Nat Commun. 6: 1-11.*

11.The time described in line 291 does not match Figure 4e.

*Response: Sorry for such confusion. We have corrected the value in the revised version as: ~6.5 Kya and ~4.8 Kya at Line 306. They match Fig. 4e now.*

12.The analysis and results of population genetics are relatively routine. What is curious is how the author distinguishes the introgression from wild populations from the incomplete lineage sorting in the domestication process? I don't think the ABBA test can rule out the influence of ILS, especially for domesticated populations, where the differentiation of the population structure is prone to false positive results. Another question is, since it is mentioned in this article that there are many structural variations and differences in gene content between the YY and XX genomes, whether the use of different genotypes as reference genomes will affect the analysis of population genetics, especially the selected genes and introgressed regions?

*Response: Yes, it was difficult to distinguish the introgression from incomplete lineage sorting (ILS) since these issues were firstly found in phylogenetics studies. However, we searched the literature and found a new method published by Edelman et al. (2019), which using branch length to quantify introgression and distinguish true introgression from ILS in butterfly radiation. The method is called “quantifying introgression via branch lengths (QuIBL)”. In this method, if the absence of introgression, it expects internal branch lengths of triplet topologies discordant with the species tree (due to ILS) to be exponentially distributed. However, if introgression has occurred, then their distribution should have that same exponential component but also include an additional component with a non-zero mode corresponding to the time between the introgression event and the most recent common ancestor of all three species. QuIBL use BIC (a Bayesian information criterion) score to evaluate the probability of the model of non-ILS topologies with the model of ILS topologies for each of triplet trees. If BIC score for model of non-ILS topologies is significant lower than that of ILS topologies, it indicates the introgession.*

*We used QuIBL to quantify six potential introgressed regions detected by fd_M statistics (Fig 6c-k: Ste_Introgressor (Chr3:106.09-108.63 Mb); Ste_Introgressor (Chr6:143.22-145.43 Mb); Stu_Introgressor (Chr4:59.93-65.83 Mb); Stu_Introgressor (Chr6:141.19-143.29 Mb); India_Introgressor (Chr2:115.44-118.27 Mb); India_Introgressor (Chr5:76.46-78.87 Mb)). The results showed the model of non-ILS topologies has significantly lower BIC scores (with a strict cutoff of delta BIC, dBIC = BIC1st-BIC2st >30, indicates an extremely difference) than the model of ILS topologies for each of six introgressed regions (Fig. S25a). It indicated the six regions detected by fd_M statistics were caused by introgression rather than ILS. The proportion of belonging to the non-ILS topologies is significantly positive correlated with internal branch length, and enriched in nearly 100% proportion (Fig. S25b). Moreover, we inferred that > 90% (median value) of discordant loci are introgressed for each of six introgressed regions (Fig S25c). Overall, the tests using the QuIBL methods verify the six potential introgressed regions detected by fd_M statistics as the true introgression. Please see the analyses at Methods: Line 687-698, Results: Line 343-354; and Fig. S25.*

*Reference: Edelman, N. B., et al. (2019). Genomic architecture and introgression shape a butterfly radiation. Science. 366(6465): 594-599.*

*Thanks for your concern about using different reference genomes for population analyses. Basically, the choice of different reference genomes affects analyses such as the detection of single nucleotide polymorphisms (SNPs) and structure variations (SVs). As clear now from pan-genome analyses those large variations exist among genomes within a species, and some authors suggested that "plant pan-genomes are the new reference" (Bayer et al. 2020). However, for population genomic analyses, one reference genome, not multiple reference genomes, should be used to generate data from a common reference for various analyses. For this reason, there shouldn't be an issue that we used the supermale YY genome as a reference genome for population genomic analysis, despite the differences with XX genome.*

*Further, the focus of our manuscript is supermale YY, and not XX genome, since the used XX genome has already been published. Meanwhile, we did not call the variants based on XX genome, so the comparison of selection and introgression signals between two reference genomes was not conducted. Therefore, the SVs or gene content that influences the regions of selection and introgression can not be ruled out currently here, and we intend to study this in a future paper.*

*Reference: Bayer, P.E., et al. (2020). Plant pan-genomes are the new reference. Nature plants. 6: 914-920.*

**Second round of review**

**Reviewer 1**

The authors have addressed most of my previous comments. Still, there are some remaining issues.

When discussing the use of young plant sex chromosomes to study initial stages of sex chromosome evolution, I think the authors should take more care to not confuse recently evolved systems with systems simply not evolving suppressed recombination and thus none of the secondary characteristics classically associated with plant sex chromosomes (such as repetitive sequences, genetic differentiation and ultimately degeneration). Recent papers suggest that recombination suppression may or may not evolve (Renner & Müller 2021: https://doi.org/10.1038/s41477-021-00884-3, Charlesworth 2021: https://doi.org/10.1111/evo.14196). The authors write that the SDRs of Vitis and Populus (balsamifera) are smaller and thus more recently evolved (L88-90). I do not agree with this statement. The SDR of Vitis appears to be shared with that of Muscadinia (Massonnet et al. 2020: https://doi.org/10.1038/s41467-020-16700-z) and thus predates the split of those taxa. This indicates that the Vitis SDR may be quite ancient despite its small size. Similarly, the SDR of Populus balsamifera was shown to predate poplar speciation (Müller et al. 2020: https://doi.org/10.1038/s41477-020-0672-9, Zhou et al. 2020: https://doi.org/10.3390/genes11080843) and can thus not be considered recently evolved.

Identification of sex chromosome and SDR (L167-186): I appreciate the addition of the GWAS and the clarification on the definition of the SDR boundaries. Even though the authors now specify the contigs on which the SDR borders are based, I still do not see the formal cutoffs used. The values for each measure are very precise, for example "high density of male-specific SNPs distribution (131.64-163.44 Mb)", but what was the specific cutoff for "high density"? The cutoffs are unclear to me for all measures, that is Fst, Tajima's D and GWAS SNPs.

More importantly, I now agree even less with the "consensus" sex-determining region defined by the authors. The GWAS peak, the male-specific SNPs and the Tajima's D differences all point to an SDR starting at around 145 Mb, not 131 (by the way, it would be nice to use a more common bin size for the x axis, for example 20 not 22 Mb - the axis labels of 21, 43, 65 etc. are completely unintuitive). The segregation pattern in the F1 family appears to erroneously extend the SDR to the left. It is not uncommon to overestimate the size of SDRs based on genetic mapping within families. The GWAS peak and male-specific SNPs should be much more representative of the actual SDR as they account for rare recombination events. I do therefore not consider the conclusions in lines 183-186 and 398-401 adequate.

The GWAS results, but also the male-specific SNPs and sex co-segregating bins, are surprisingly noisy. This a common issue when not supplying both sex chromosomes for mapping (e.g. Zhou et al., 2020: https://doi.org/10.1186/s13059-020-1952-4 or Zhou et al., 2020: https://doi.org/10.3390/genes11080843). A clean delineation of sex-linked markers could probably be achieved by mapping the re-sequencing data against an XY reference genome. As analyzed now, all the X-specific genes, which are even shown in Table S8, can only map to their next closest homolog in the YY assembly. This might partially be solved by using more stringent filtering of the SNPs based on mean coverage values in males and females (- I could not find the information on the expected sequencing coverage of the re-sequenced accessions anywhere in the manuscript, but the filtering of 2<DP<60 appears exceedingly relaxed -) but most reliably by re-mapping.

Figure 5: in the legend for panel e it reads "expression of candidate genes between wild and cultivars". The table for internode tissue includes two wild accessions, the table for leaf tissue four. I did not find any explanation which genotypes are used for this analysis (not even whether these data represent S. tetranda or S. turkestanica) or why different numbers were chosen for the different tissues. For panel f, it is also not clear for me from which genotype those pictures were taken and how that genotypes were chosen.

Figure 6: in the legend only "f_dM" is shortly explained, "d" and "f_d" are not mentioned.

Regarding the language, I agree that the text has been improved. While I found the science mostly clear now, I could unfortunately still not enjoy reading the manuscript. To me the text still seemed to contain way too many wrong sentences. This is even true for the abstract (with several missing words and wrong tenses) and continues throughout the manuscript. Perhaps this will be fixed during copyediting?

Minor comments:

L 46 (abstract) "A high-quality YY genome was assembled into a draft assembly" does not sound right. The high-quality corresponds to the assembly not the genome. Should it read "A high-quality draft assembly was generated from a YY genotype"? But then "high-quality" and "draft" seem to contradict each other.

I think the evolution of dioecy should not be called a "mystery" (L72). There are several theoretical models and accumulating empirical data.

Figure S18: The transparent colors improved the figure. However, the S. tetrandra individuals (grey points) from the overview PCA are still not visible in the zoomed-in lower panel. What happened to those individuals?

The authors did not comment on my previous question regarding the lower genetic diversity within the wild S. tetrandra population. I only know examples where the domestication bottleneck reduced the genetic diversity of crop plants. How do the authors explain their opposite finding?

Lines 317-328: this part reads as if all those genes were functionally characterized in spinach. I think it is important to explicitly mention that those genes have only been functionally characterized in Arabidopsis and write that they "may" or "might be involved in/regulate/affect/confer" the discussed phenotypes in spinach. The same is true for the section on introgressions. In several cases, the statements seem to be too strong (e.g. "ZAT10-like (YY37988) in Chr4 introgressed from S. turkestanica enhance tolerance to osmotic stress" followed by an Arabidopsis paper citation). I would tone down those statements or add citations to the relevant spinach literature, which in some cases is done.

L393: the supermale is not actually a doubled haploid line, but simply the offspring of a cosexual individual. I would therefore avoid this potentially ambiguous terminology.

**Reviewer 2**

The authors have addressed my concerns, they have provided the evidence and revised the manuscript. Just some small comments:

Line 142-149: "The resulting YY contig assembly yielded 948 Mb of sequences…Finally, 950 Mb (99.79% anchored) of the YY genomes were anchored into six pseudo-chromosomes". Why the sequence anchored to the chromosome is larger than the assembled genome?

Fig. 2f: "red asterisks indicate Y-specific insertions". But it's not marked in the figure.

**Authors Response**

**Point-by-point responses to the reviewers’ comments:**

Reviewer 1#:

The authors have addressed most of my previous comments. Still, there are some remaining issues.

When discussing the use of young plant sex chromosomes to study initial stages of sex chromosome evolution, I think the authors should take more care to not confuse recently evolved systems with systems simply not evolving suppressed recombination and thus none of the secondary characteristics classically associated with plant sex chromosomes (such as repetitive sequences, genetic differentiation and ultimately degeneration). Recent papers suggest that recombination suppression may or may not evolve (Renner & Müller 2021: https://doi.org/10.1038/s41477-021-00884-3, Charlesworth 2021: https://doi.org/10.1111/evo.14196). The authors write that the SDRs of Vitis and Populus (balsamifera) are smaller and thus more recently evolved (L88-90). I do not agree with this statement. The SDR of Vitis appears to be shared with that of Muscadinia (Massonnet et al. 2020: https://doi.org/10.1038/s41467-020-16700-z) and thus predates the split of those taxa. This indicates that the Vitis SDR may be quite ancient despite its small size. Similarly, the SDR of Populus balsamifera was shown to predate poplar speciation (Müller et al. 2020: https://doi.org/10.1038/s41477-020-0672-9, Zhou et al. 2020: https://doi.org/10.3390/genes11080843) and can thus not be considered recently evolved.

*Reply: Thanks very much for correcting errors. It is true that not all sex determination system evolved an SDR with suppressed recombination. Sex-linked regions could potentially remain as small as the initial sex-determining mutations, or could subsequently evolve into large non-recombining regions at later stages of sex chromosome evolution as supported by more recent studies (Renner & Müller 2021; Charlesworth 2021). We re-phrased our statement for the introduction of sex chromosome evolution in current version to prevent confusion and cited two articles (Renner & Müller 2021; Charlesworth 2021) to support our revised statements (please see Introduction).*

*We made a mistake by saying that “small SDR of Vitis and Populus balsamifera as recently evolved” in the previous version of our manuscript. We agree with reviewer’s point of view that “SDRs of Vitis and Populus balsamifera are smaller but quite ancient”. We have corrected the mistake by rephrasing the sentence to make it clear. (Please see Introduction).*

*References*

*Renner SS, Müller NA. Plant sex chromosomes defy evolutionary models of expanding recombination suppression and genetic degeneration. Nat Plants 2021, 7:392-402.*

*Charlesworth D. When and how do sex‐linked regions become sex chromosomes? Evolution 2021, 75:569-581.*

Identification of sex chromosome and SDR (L167-186): I appreciate the addition of the GWAS and the clarification on the definition of the SDR boundaries. Even though the authors now specify the contigs on which the SDR borders are based, I still do not see the formal cutoffs used. The values for each measure are very precise, for example "high density of male-specific SNPs distribution (131.64-163.44 Mb)", but what was the specific cutoff for "high density"? The cutoffs are unclear to me for all measures, that is Fst, Tajima's D and GWAS SNPs.

More importantly, I now agree even less with the "consensus" sex-determining region defined by the authors. The GWAS peak, the male-specific SNPs and the Tajima's D differences all point to an SDR starting at around 145 Mb, not 131 (by the way, it would be nice to use a more common bin size for the x axis, for example 20 not 22 Mb - the axis labels of 21, 43, 65 etc. are completely unintuitive). The segregation pattern in the F1 family appears to erroneously extend the SDR to the left. It is not uncommon to overestimate the size of SDRs based on genetic mapping within families. The GWAS peak and male-specific SNPs should be much more representative of the actual SDR as they account for rare recombination events. I do therefore not consider the conclusions in lines 183-186 and 398-401 adequate.

*Reply: Thanks for your suggestion to further improve our definition of clear boundaries of SDR. We now agree with reviewers’ point that the segregation pattern in the genetic mapping using an F_1_ segregating population appears to erroneously extend the SDR to the left, therefore overestimating the size of SDRs.*

*We have changed new definition of SDR boundaries as Chr1:145.31-162.73 Mb (Size = 17.42 Mb). Several lines of evidence supported these new SDR boundaries, especially the GWAS mapping, genetic differentiation (Fst statistics) and Tajima’s D statistics between females and males re-sequenced data, as well as male-specific SNPs. Please see the new results in Main-text Results: at Line 155-181 and Fig 1c&d (see following).*

*We added the formal cutoffs for the density of sex co-segregating contigs, GWAS mapping SNPs, Fst statistics, and Tajima's D value and updated the definition of the SDR boundaries. Please see Main-text Results: at Line 155-181; and Methods: Line 546-584; and Fig 1c&d (see following).*

*To adapt to the new SDR region, we updated the contents related to SDR analysis. Please see updated results at Line 183-223 and Line 224-263. We also remade the corresponding figures and tables which were related to the SDR analysis: (Fig 1c&d, Fig 2, Fig 3a; FigS4, Fig S5c, Fig S6, FigS12, Fig S13, and FigS14 as well as Table S6, Table S7, Table S8, Table S9, Table S10, Table S11, and Table S12).*

*Besides, we also changed the bin size for the x-axis of Fig 1d. It became intuitive.*

The GWAS results, but also the male-specific SNPs and sex co-segregating bins, are surprisingly noisy. This a common issue when not supplying both sex chromosomes for mapping (e.g. Zhou et al., 2020: https://doi.org/10.1186/s13059-020-1952-4 or Zhou et al., 2020: https://doi.org/10.3390/genes11080843). A clean delineation of sex-linked markers could probably be achieved by mapping the re-sequencing data against an XY reference genome. As analyzed now, all the X-specific genes, which are even shown in Table S8, can only map to their next closest homolog in the YY assembly. This might partially be solved by using more stringent filtering of the SNPs based on mean coverage values in males and females (- I could not find the information on the expected sequencing coverage of the re-sequenced accessions anywhere in the manuscript, but the filtering of 2<DP<60 appears exceedingly relaxed -) but most reliably by re-mapping.

*Reply: It is true that using a reference genome from an XY genotype and filtering SNPs with high stringency would eliminate noises from background. Unfortunately, there’s no XY genome assembly available now. However, to improve the quality of reads mapping, we re-mapped re-sequenced genomes and filtered variants with more strict parameters as suggested by the reviewer.*

*To obtain high-quality SNPs for defining the sex determination region (SDR), we applied repeat-masked YY genome for mapping sequence reads, and retained unique mapped reads for variants calling.*

*The genomes of the F_1_ population used for SDR definition (40 female and 39 male accessions not including patents) were sequenced at depth ranged from 8.5× – 16.1× with an average of 12.4× (Additional file: Part2. Sex-chromosome analysis; Table S6). The genomes of the natural population used for SDR definition (26 female and 44 male accessions) were sequenced at depth ranged from 4.1× - 7.7× with an average of 6.1× (Additional file: Part2. Sex-chromosome analysis; Table S13). The density distribution of mean read mapping depth (DP) per site of female and male accessions onto repeat-masked YY genome for F_1_ population and natural population were shown in Fig S4a, b. We filtered depths of variants based on these density distribution plots (Fig S4 following).*

*In the F_1_ mapping population, only sites with missing data < 20%, minor allele frequency (MAF) > 0.05, mean mapping depth (DP) per site in between 3-35, depth by quality (DQ) > 2, genotype quality (GQ) > 10 (the sites with less than 10% chances of genotype calling error), and mapping quality (minQ) > 10 (the sites with less than 10% chances of wrong absence/presence of SNPs) were kept to screen sex co-segregating bins (Additional file: Part2. Sex-chromosome analysis).*

*In natural population, we applied only sites with missing data < 25%, minor allele frequency (MAF) > 0.05, mean mapping depth (DP) per site in between 2-20, minimum alleles = 1, maximum alleles = 2, and mapping quality (minQ) > 20 as cutoffs to remove low quality SNPs for GWAS mapping, as well as calculation of Fst statistics, density of male-specific SNPs, and Tajima’s D values (Additional file: Part2. Sex-chromosome analysis). We also summarized this part in the current method (Maintext Methods: Line 546-584)*

*After filtering SNPs based on criteria described above from the F_1_ mapping population, 177,414 SNPs with nn x np pattern (homozygous in the female parent and heterozygous in the male parent) were kept for screening sex co-segregating bins, resulting in 322 sex co-segregating contigs (2,690 SNPs). We further calculated the density of sex co-segregating contigs in a 100-Kb sliding window. The top 1% density of sex co-segregating contigs (cutoff value =1.29) were identified as regions with sex co-segregation (Chr1: 141.1-165.7 Mb). We found the noises of sex co-segregating bins were reduced compared with the relaxed parameters and cutoffs in previous version.*

*For the natural population, 4,844,193 high-quality SNPs after stringent filtering were used for GWAS mapping with -log10(P) = 6 as the cutoff to identify sites with significant sexual phenotype-genotype associations which generated peaks from Chr1: 145.0-162.7 Mb. The top 1% density of male-specific SNPs in 20-Kb sliding windows was selected to characterize the distribution of male-specific SNPs across sex chromosomes. This enabled the identification of Chr1: 143.6-163.5 Mb as the region featured by aggregative presence of male-specific SNPs. In addition, the Tajima’s D values and Fst statistics were also calculated to define the SDR region. The top 5% Fst statistics (in 1000-Kb sliding window) identified the region from Chr1: 145.3-167.0 Mb as the region diverged between female and male genomes. The top 5% male/female ratio of Tajima’s D values (in 200-Kb sliding window) showed the region from Chr1:141.4-163.6 Mb bearing diverged Tajima’s D values between the two sexes.*

*Finally, Chr1:145.31-162.73 Mb (Size = 17.42 Mb) is the overlapped region across these multiple lines of evidence described above and thus was defined as SDR of the Y chromosome in spinach.*

*Please see new results in Main-text Results: at Line 155-181; Methods: Line 546-584. As well as Additional file: Part2. Sex-chromosome analysis.*

Figure 5: in the legend for panel e it reads "expression of candidate genes between wild and cultivars". The table for internode tissue includes two wild accessions, the table for leaf tissue four. I did not find any explanation which genotypes are used for this analysis (not even whether these data represent S. tetranda or S. turkestanica) or why different numbers were chosen for the different tissues. For panel f, it is also not clear for me from which genotype those pictures were taken and how that genotypes were chosen.

*Reply: Figure 5: for panel e, we displayed the expression of candidate genes for two wild accessions (S. tetranda: PI677114 and S. turkestanica: PI677111) and two S. oleracea cultivars (Sp75 and Cornell-NO. 9) for internode (top) and leaf tissues (bottom).*

*Two replications of each sample from all accessions were used in differential expression analysis. In the previous version, we mistakenly described the data of two replications from each wild type accession for leaf samples. We have corrected this mistake by describing the average value of two replications for leaf samples of each wild accession (2 columns, now same as for internode). The 1^st^ internode (bottom to top of plant) and leaf from middle section of plant were chosen for both wild (S. tetranda: PI677114 and S. turkestanica: PI677111) and cultivated (Sp75 and Cornell-NO. 9) genotypes. (Plant materials and methods are described in Additional file: Part4. Resequencing and Population genomics)*

*For genotype selection, at the beginning of this experiment, we screened many wild accessions and chose the ones having extreme phenotypic differences comparing with the phenotypes of the cultivars. So wild accessions S. tetranda: PI677114 and S. turkestanica: PI677111 were selected for RNA-sequencing and phenotyping as showed in Fig. 5-panel f. For cultivated accessions, we screened USDA and Chinese cultivars, and most of them showed similar phenotype, so Chinese cultivar (Sp75), USDA accession (Cornell-NO. 9) were selected for RNA-seq analysis. As Sp75 and Cornell-NO. 9 phenotypes (f1-13) were similar, we showed images from Sp75. For wild accessions, both S. tetranda: PI677114 and S. turkestanica: PI677111 are shown in Figure 5 f6-f15. Please refer to Figure 5f, Figure 5f legend, and Additional file: Part4. Resequencing and Population genomics.*

Figure 6: in the legend only "f_dM" is shortly explained, "d" and "f_d" are not mentioned.

*Reply: Sorry, we made a mistake for the Figure plot. In previous version, we have deleted the “d” and “f_d” in main text, and we forgot to remove them in Figure 6. To make it clear, we now removed the “d” and “f_d” in new Figure 6 as well.*

*The “d” and “f_d” statistic is closely related to "f_dM" statistic. "f_dM" is more stringent than the “d” and “f_d” for detecting genome-wide introgression (Martin et al. 2015; Malinsky et al. 2015). Therefore, we finally only use "f_dM" to show the results and final figure. Please see new Figure 6.*

*References*

*Martin SH, Davey JW, Jiggins CD: Evaluating the use of ABBA–BABA statistics to locate introgressed loci. Molecular Biology and Evolution 2015, 32:244-257.*

*Malinsky, M., Challis, R. J., Tyers, A. M., Schiffels, S., Terai, Y., Ngatunga, B. P., ... & Turner, G. F. (2015). Genomic islands of speciation separate cichlid ecomorphs in an East African crater lake. Science, 350(6267), 1493-1498.*

Regarding the language, I agree that the text has been improved. While I found the science mostly clear now, I could unfortunately still not enjoy reading the manuscript. To me the text still seemed to contain way too many wrong sentences. This is even true for the abstract (with several missing words and wrong tenses) and continues throughout the manuscript. Perhaps this will be fixed during copyediting?

*Reply: We have used a professional editing service Alum Canyon Editing, LLC provided by Mary Ann Cushman, a native English speaker. The English of the manuscript was corrected and polished.*

Minor comments:

L 46 (abstract) "A high-quality YY genome was assembled into a draft assembly" does not sound right. The high-quality corresponds to the assembly not the genome. Should it read "A high-quality draft assembly was generated from a YY genotype"? But then "high-quality" and "draft" seem to contradict each other.

*Reply: Agree. We have revised the sentence as “A high-quality genome of a spinach YY genotype was assembled into 952 Mb in six pseudo-chromosomes, containing 26,910 annotated genes.”.*

I think the evolution of dioecy should not be called a "mystery" (L72). There are several theoretical models and accumulating empirical data.

*Reply: We have revised the sentence as “Sequencing male and female genomes and defining sex determination regions (SDRs) in dioecious angiosperm species will contribute to uncover the evolutionary process leading to dioecy.”.*

Figure S18: The transparent colors improved the figure. However, the S. tetrandra individuals (grey points) from the overview PCA are still not visible in the zoomed-in lower panel. What happened to those individuals?

*Reply: Sorry again for this unreadable figure. We have replotted it and made the colors transparent in a zoomed-in panel and highlighted the samples of S. tetrandra. Now they are clear and shown in new Figure S19.*

The authors did not comment on my previous question regarding the lower genetic diversity within the wild S. tetrandra population. I only know examples where the domestication bottleneck reduced the genetic diversity of crop plants. How do the authors explain their opposite finding?

*Reply: Sorry again for being unclear about this issue. The lower genetic diversity within the wild S. tetrandra population is an artifact of a small sample size, not representing the entire scope and extent of genetic diversity in this species. Although the genetic diversity within wild S. tetrandra population is lower, the genome-wide diversity is not always lower at different sliding windows (see Fig S17c). Especially, in the key selective sweep region (Chr3: 106,112,216 - 108,343,948) including flanking regions, the genetic diversity of both wild S. turkestanica and S. tetrandra is higher than that of cultivar species S. oleracea. The key swept regions were supported by the genetic diversity comparison of cultivars with both wild species (Fig 5a-c; Fig S17c). We have revised the relevant part as follows:*

*“The nucleotide diversities (π) of S. oleracea (0.48 ± 0.32×10^-3^) and S. turkestanica (0.43 ± 0.29×10^-3^) were closer to each other but twice that of S. tetrandra (0.12 ± 0.10×10^-3^), which could be an artifact of a small sample size, not representing the scope and extent of genetic diversity in S. tetrandra.”*

Lines 317-328: this part reads as if all those genes were functionally characterized in spinach. I think it is important to explicitly mention that those genes have only been functionally characterized in Arabidopsis and write that they "may" or "might be involved in/regulate/affect/confer" the discussed phenotypes in spinach. The same is true for the section on introgressions. In several cases, the statements seem to be too strong (e.g. "ZAT10-like (YY37988) in Chr4 introgressed from S. turkestanica enhance tolerance to osmotic stress" followed by an Arabidopsis paper citation). I would tone down those statements or add citations to the relevant spinach literature, which in some cases is done.

*Reply: We revised this part as suggested for discussing candidate genes found in domestication and introgression, like following:*

*“Plant height and leaf size are key edibility traits that have been improved during spinach domestication [31]. A strong sweep signal from Chr3:106,112,216–108,343,948 was enriched for annotations of genes involved in cell differentiation and polyamine metabolism whose homologs have been functionally characterized in Arabidopsis (Fig. 5a-c; Additional file: Fig. S22b). Three genes whose Arabidopsis homologs might be participating in either stem cell elongation via polyamine synthesis (AIH) [40] or cell expansion (COBRA4) [41] and cell division (CDC123) [42] showed cultivar-biased expression in the stem internode, while the TCP17, whose Arabidopsis homolog may negatively affect shoot morphogenesis [43], exhibited wild-biased expression in stem internode (Fig. 5d,e; Additional file: Fig. S23; Table S14). PYM, whose Arabidopsis homolog may affect leaf primordium cell enlargement, showed cultivar-biased expression, while TCP17, which may negatively regulate leaf development, exhibited wild-biased expression in leaves [44, 45] (Fig. 5e; Additional file: Fig. S23). Moreover, morphological, and cytological comparisons of S. oleracea cultivars to wild species showed relatively elongated internodes exhibiting longitudinal cell elongation and enlarged leaf area due to cell number proliferation, both of which might be regulated by these candidate genes (Additional file: Fig. 5f).”*

L393: the supermale is not actually a doubled haploid line, but simply the offspring of a cosexual individual. I would therefore avoid this potentially ambiguous terminology.

*Reply: True. The supermale YY genotype is the offspring of an androdioecious plant, not an actual “double haploid” line. We have removed “doubled haploid” in the text.*

Reviewer 2#:

The authors have addressed my concerns, they have provided the evidence and revised the manuscript. Just some small comments:

Line 142-149: "The resulting YY contig assembly yielded 948 Mb of sequences…Finally, 950 Mb (99.79% anchored) of the YY genomes were anchored into six pseudo-chromosomes". Why the sequence anchored to the chromosome is larger than the assembled genome?

*Reply: Sorry for such confusion. This is because the anchored chromosome-level genome assembly was scaffolded from the original contig-level genome assembly and linked adjacent contigs by adding the gaps with 100bp Ns. A total 4478100 bp (~4.48 Mb) gaps was added to form six pseudo-chromosomes with 950323615 bp (~950 Mb) size. The unanchored contigs are 1982396 bp (~1.98 Mb) in total. The total genome size is 950323615 bp + 1982396 bp = 952306011 bp (~952 Mb). We have revised the description to make it clearer.*

Fig. 2f: "red asterisks indicate Y-specific insertions". But it's not marked in the figure.

*Reply: We re-plotted the insertion time of LTR-RTs from collinear regions and inversions for both SDR and the X counterpart, and revised Figure 2 legend accordingly.*

**Third Round of review**

**Reviewer 1**

I thank the authors for addressing all of my previous comments.

**Reviewer 2**

The authors have addressed my major concerns from the previous submission, However, I found that in the current draft of the manuscript, the conclusion that "The nascent sex chromosomes of spinach … SDR and the highly repetitive genome (74%) of spinach." in lines 24-27 of the abstract is not well supported. It is theorized that sex chromosomes originated from autosomes, and recent studies have shown frequent turnover of sex chromosomes in some plant lineages. Therefore, the results in the manuscript cannot determine whether the sex chromosomes of spinach evolved from autosomes or ancient sex chromosomes, and outgroup species are needed as a reference. In addition, 1.98 Mya is the estimated time of inversion 1, not the origin time of the insertion containing the sex determining gene. In fact, the origin time of insertion is not given in the manuscript, which may occur earlier than the inversion. The contribution of the SDR repetitive sequence to the high repetitive sequence of the whole genome is not specifically reflected in the manuscript, and the conclusion is too exaggerated. In addition, for Fig2e, the possible reasons for the difference ka/ks between inversion 1 and inversion 2 should also be explained.

**Authors Response**

**Point-by-point responses to the reviewers’ comments:**

Reviewer 2#:

The authors have addressed my major concerns from the previous submission, However, I found that in the current draft of the manuscript, the conclusion that "The nascent sex chromosomes of spinach … SDR and the highly repetitive genome (74%) of spinach." in lines 24-27 of the abstract is not well supported. It is theorized that sex chromosomes originated from autosomes, and recent studies have shown frequent turnover of sex chromosomes in some plant lineages. Therefore, the results in the manuscript cannot determine whether the sex chromosomes of spinach evolved from autosomes or ancient sex chromosomes, and outgroup species are needed as a reference. In addition, 1.98 Mya is the estimated time of inversion 1, not the origin time of the insertion containing the sex determining gene. In fact, the origin time of insertion is not given in the manuscript, which may occur earlier than the inversion. The contribution of the SDR repetitive sequence to the high repetitive sequence of the whole genome is not specifically reflected in the manuscript, and the conclusion is too exaggerated. In addition, for Fig2e, the possible reasons for the difference ka/ks between inversion 1 and inversion 2 should also be explained.

*Reply: We agree that there is no direct evidence to designate spinach sex chromosomes as the product of evolution from autosomes and we removed both statements of “nascent sex chromosome” and “evolved from autosomes”. Also, it is true that the 1.98 Mya is not related to insertion time. Because this estimated time is based on calculation of paired X and Y genes within inversion, thus the time of Insertion cannot be ruled out now. We corrected the statement in current Abstract.*

*We agree that fraction and insertion time of intact LTR-RTs within SDR is not corresponded to whole genome-wide repetitive sequences. Therefore, these regional retrotransposons only explained the SDR size but not the 74.4% highly repetitive spinach genome. We rephrased sentence accordingly in current Abstract.*

In addition, for Fig2e, the possible reasons for the difference ka/ks between inversion 1 and inversion 2 should also be explained.

*Reply: In SDR of spinach, Inversion 1 and 2 were dated at different time points (1.98 and 1.63MYA) and assigned into two strata. Genes with various functions from these two regions may under different selection pressures. This possible reason was summarized in Discussion part of the revised manuscript.*
